# Global profiling of co- and post-translationally *N*-myristoylated proteomes in human cells

**DOI:** 10.1038/ncomms5919

**Published:** 2014-09-26

**Authors:** Emmanuelle Thinon, Remigiusz A. Serwa, Malgorzata Broncel, James A. Brannigan, Ute Brassat, Megan H. Wright, William P. Heal, Anthony J. Wilkinson, David J. Mann, Edward W. Tate

**Affiliations:** 1 Department of Chemistry, Imperial College London, Exhibition Road, London SW7 2AZ, UK; 2 Department of Life Sciences, Imperial College London, Exhibition Road, London SW7 2AZ, UK; 3 York Structural Biology Laboratory, Department of Chemistry, University of York, York YO10 5DD, UK; 4 Department of Chemistry, Institute of Chemical Biology, Imperial College London, Exhibition Road, London SW7 2AZ, UK

## Abstract

Protein *N*-myristoylation is a ubiquitous co- and post-translational modification that has been implicated in the development and progression of a range of human diseases. Here, we report the global *N*-myristoylated proteome in human cells determined using quantitative chemical proteomics combined with potent and specific human *N*-myristoyltransferase (NMT) inhibition. Global quantification of *N*-myristoylation during normal growth or apoptosis allowed the identification of >100 *N*-myristoylated proteins, >95% of which are identified for the first time at endogenous levels. Furthermore, quantitative dose response for inhibition of *N*-myristoylation is determined for >70 substrates simultaneously across the proteome. Small-molecule inhibition through a conserved substrate-binding pocket is also demonstrated by solving the crystal structures of inhibitor-bound NMT1 and NMT2. The presented data substantially expand the known repertoire of co- and post-translational *N*-myristoylation in addition to validating tools for the pharmacological inhibition of NMT in living cells.

## Abstract (web summary)

Protein *N*-myristoylation is a ubiquitous modification implicated in the regulation of multiple cellular processes. Here, Thinon *et al.* report the development of a general method to identify *N*-myristoylated proteins in human cells and identify over 100 endogenous post- and co-translational substrates of *N*-myristoyltransferase.

## Graphical abstract

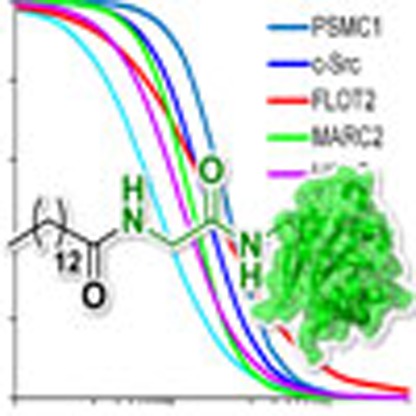

Protein *N*-myristoylation is a co- and post-translational modification in eukaryotes catalysed by the enzyme *N*-myristoyltransferase (NMT), which transfers myristate from myristoyl coenzyme A (Myr-CoA) to the N-terminal glycine of a wide range of substrate proteins. Both human NMT isozymes, NMT1 and NMT2, are expressed in most tissues and have been implicated in the development and progression of diseases including cancer^1^, epilepsy^2^, Alzheimer’s disease^3^, Noonan-like syndrome^4^ and viral and bacterial infections^5^. *N*-myristoylation is necessarily preceded by proteolysis to reveal an N-terminal glycine, the only completely conserved motif across all known NMT substrates. This may occur either co-translationally when methionine aminopeptidase (MetAP) acts on an N-terminal ‘MG’ motif at the ribosome, or post-translationally at an internal site (Fig. 1a)^6^,^7^. Well-characterized substrates include oncogene products, such as the tyrosine kinases pp60c-src and pp60c-yes, and BID, a key mediator of apoptosis^1^. The potential scope of NMT substrates is substantial; N-terminal ‘MG’ is present in ca. 7% of all gene products and is an excellent substrate for MetAP, while site-specific proteolysis is ubiquitous in cells, an example being caspase activity during apoptosis^8^.

Despite its importance in health and disease, direct identification of the *N*-myristoylated proteome in cells has been limited to non-native low-throughput systems (for example, overexpression of artificial protein constructs), and the vast majority of potential co- and post-translational NMT substrates have not been characterized in a native cellular context. We describe here a chemistry-based approach to profile selectively the co- and post-translationally *N*-myristoylated proteome in human cells during normal cell function and during apoptosis, which combines quantitative chemical proteomics with a potent and specific human NMT inhibitor. We present X-ray crystal structures of human NMT1 and NMT2 providing structural evidence for a highly conserved protein substrate-binding pocket, and demonstrate selective target engagement and pharmacological knockdown of *N*-myristoylation by an NMT inhibitor in live cells. We also introduce a novel reagent that facilitates analysis of the N-terminally modified peptide across a wide range of substrates, including direct identification of sites of post-translational myristoylation. Global analysis of *N*-myristoylation results in the identification of >100 *N*-myristoylated human proteins, the majority of which are identified for the first time in a native cellular context. This global experimental strategy to define the *N*-myristoylated proteome greatly expands the known repertoire of co- and post-translational *N*-myristoylation (PTMyr), and provides an approach to quantify inhibition of NMT across dozens of substrate proteins simultaneously in human cells.

## Results

### The targets of myristate analogue YnMyr in human cells

**Figure 1:**
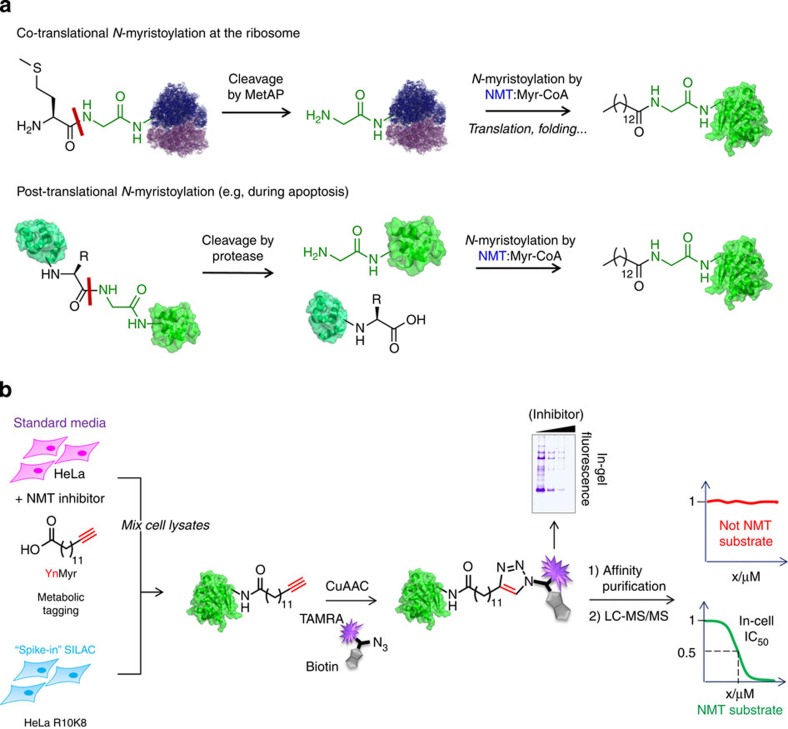
Quantitative chemical proteomic strategy for global profiling of NMT substrates in mammalian cells. (**a**) N-terminal *N*-myristoylation occurs co-translationally following action of methionine aminopeptidase (MetAP) at the ribosome, or post-translationally following cleavage by a protease. An N-terminal glycine residue is the only fully conserved element of the substrate. (**b**) Chemical proteomic workflow. Cells cultured in normal media with YnMyr and NMT inhibitor were lysed and spiked with a fixed quantity of lysate from cells cultured in SILAC media (R10K8 labelled) with YnMyr. Labelled proteins were captured by CuAAC with AzTB, affinity enriched and classified as substrates (or non-substrates) according to the response of enrichment to inhibition determined by quantitative proteomics (nanoLC-MS/MS, MaxQuant, Perseus and GraFit 7.0).

Lipids tagged with an alkyne or azide moiety have emerged as powerful tools to study protein lipidation in cells by virtue of their capacity for metabolic incorporation and selective ligation to a range of reporter labels^9^. One such example is YnMyr (tetradec-13-ynoic acid), a tagged myristate analogue that has been shown to label a variety of proteins in cells (Fig. 1b)^10^,^11^,^12^, and that was recently used to profile the myristoylated proteome in a protozoan parasite^13^. While these studies demonstrate the potential of YnMyr as a probe, it has yet to be applied to comprehensive identification of substrates and non-substrates in the complex proteome of human cells. To address this challenge, we sought to develop robust quantitative methods that can provide objective measures to identify NMT substrates across the proteome in living cells.

Initial studies using a fluorogenic assay for NMT^14^ showed that YnMyr in its CoA-ester form (YnMyr-CoA, see Supplementary Methods for synthetic details) is transferred *in vitro* by both NMT1 and NMT2 to the N-terminal glycine of a model substrate peptide with comparable efficiency to the natural lipid (Supplementary Fig. 1). Optimal metabolic incorporation of YnMyr into proteins in a human cervical cancer cell line (HeLa) was achieved after 24 h incubation at 20 μM YnMyr, as judged by in-gel fluorescence analysis following labelling of the alkyne through copper-catalysed azide-alkyne cycloaddition (CuAAC) with azido-TAMRA-PEG-Biotin (AzTB^15^, Supplementary Fig. 1). There was no adverse effect on cell viability up to 3 days with 100 μM YnMyr, and incorporation was effectively outcompeted by myristate (Supplementary Fig. 1). Selective enrichment of labelled proteins including known NMT substrates (for example, PSMC1 (ref. 16)^16^, PRKACA^17^) could be achieved through pull-down on streptavidin beads, exploiting the biotin label on AzTB (Supplementary Fig. 1). Quantitative proteomic analysis (label-free quantification (LFQ), *n*=4, false-discovery rate (FDR)=0.001) revealed that out of >250 proteins enriched more than fourfold with YnMyr (relative to a myristate control), 60% did not carry an N-terminal MG motif; some of these proteins are *S*-palmitoylated or carry a glycosylphosphatidylinositol (GPI) anchor, which may account for enrichment of these proteins in a manner unrelated to *N*-myristoylation by NMT (Supplementary Fig. 2b; Supplementary Data 1). Proteins (108) carried an annotated N-terminal ‘MG’ motif, but these correlated only moderately with NMT substrates predicted by bioinformatic tools (the Myristoylator^18^, Myr Predictor^19^). Given the significant limitations of this simple enrichment approach, we envisaged that knockdown of human NMT activity in cells in combination with quantitative chemical proteomics would provide a robust and novel method for proteome-wide experimental determination of the *N*-myristoylated proteome in human cells.

### A selective inhibitor of human NMT activity in cells

Co-translationally *N*-myristoylated proteins are generated continuously through *de novo* protein synthesis (Supplementary Fig. 1), and NMT is known to make direct interactions with the ribosome and several other proteins, in addition to its cognate substrates^2^. We considered that prompt and specific inhibition of the catalytic activity of both NMT isozymes would provide a precise and minimally disruptive tool to assist direct analysis through quantitative chemical proteomics^20^,^21^. While genetic approaches (RNA interference, gene knockout) can offer specificity, they present some drawbacks as tools to assist global quantitative chemical proteomic analysis of NMT substrates. These include loss of non-catalytic protein interactions of NMT, lethality of NMT knockouts^22^ and slow and incomplete induction of knockdown^23^, leading to lack of control over degree and timeframe of inhibition, and limited applicability to myristoylation events occurring on short timescales (for example, post-translational myristoylation during apoptosis). These considerations prompted us to seek a reagent for selective pharmacological knockdown of NMT activity.

**Figure 2:**
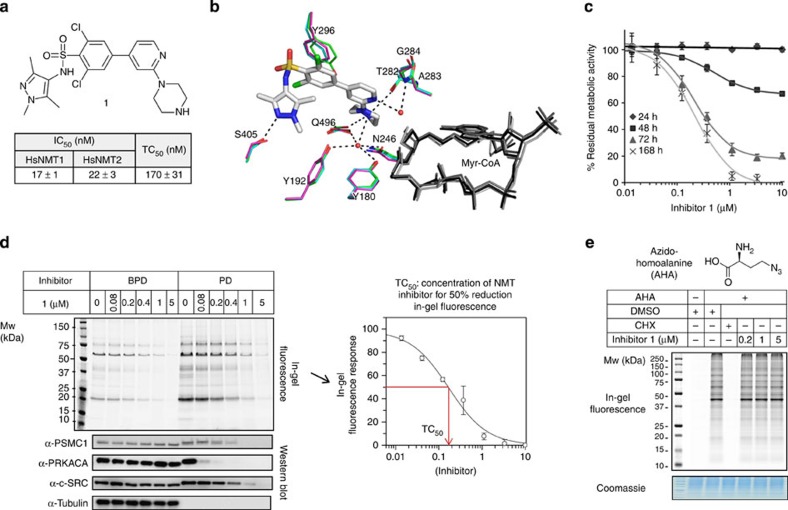
Characterization of potent and selective NMT inhibitors. (**a**) Structure and dual NMT1/NMT2 inhibitory potency of compound **1**. (**b**) Crystal structure of **1** (grey) bound to NMT1 in the presence of Myr-CoA showing key water molecules (red spheres) and polar interactions (dashed lines). Key residues (NMT1 numbering) are shown for NMT1 (blue, PDB 4C2Y) and NMT2 (pink, PDB 4C2X), and for NMT1 with **1** bound (green, PDB 4C2Z). Myr-CoA or the Myr-CoA analogue (NHM) is shown in black/grey. Image generated in PyMOL (0.99rc6, DeLano Scientific LLC, http://pymol.sourceforge.net/). (**c**) Viability (MTS assay) of HeLa cells exposed to compound **1** at concentrations and time points indicated; error bars, s.d. (*n*=6). (**d**) Compound **1** inhibits NMT activity dose-dependently in HeLa cells (BPD=before pull-down; PD=after pull-down on streptavidin-coated beads). In-gel fluorescence after YnMyr tagging was quantified (ImageJ) and the response (% relative intensity; error bars, s.d. (*n*=2)) plotted using GraFit 7.0 to determine TC_50_ (see Fig. 2a for TC_50_ determined for compound **1**). Western blots against protein substrates of NMT show dose-dependent reduction in enrichment following inhibition (tubulin: non-substrate loading control). (**e**) Cells treated with azidohomoalanine (AHA), cycloheximide (CHX) or inhibitor **1** (or DMSO vehicle), were lysed and ligated (CuAAC) to an alkyne-TAMRA reagent^67^. In-gel fluorescence demonstrates inhibition of protein synthesis by CHX, but not by **1**.

An ideal reagent for this purpose would be a dual NMT inhibitor that acts selectively in mammalian cells, with minimal disruption to normal cell function over the period of YnMyr labelling. Compound **1** (Fig. 2a)^24^ is a potent dual NMT1/NMT2 inhibitor (half-maximal inhibitory concentration (IC_50_)=17 and 22 nM, respectively)^14^ and inhibits myristoylation of an overexpressed protein construct in-cell-based microscopy assays^25^,^26^; we previously validated **1** as a highly selective tool for knockdown of NMT activity in protozoan parasites^13^. To validate this chemotype as a pharmacological tool in human cells, we determined the binding mode of **1** in a human NMT1/myristoyl-CoA complex through X-ray crystallography to 2.1 Å resolution, and compared this structure with those of binary complexes of NMT1 and NMT2 solved to 1.7 and 2.3 Å resolution, respectively (Fig. 2b; Table 1; Supplementary Fig. 3, PDB 4C2Z, 4C2Y, 4C2X). These structures provide comparative atomic resolution insight into the human NMT enzymes, showing the very similar arrangement of residues in the human NMT1 and NMT2 active sites. **1** binds at the protein substrate-binding pocket in the presence of Myr-CoA, and interacts with minimal disturbance of side-chain geometry, suggesting that it binds tightly to a pocket pre-organized by binding of myristoyl-CoA^27^, and confirming its conserved mode of action as a dual NMT1/2 inhibitor. **1** had no effect on HeLa cell viability, as measured by the metabolic activity assay (MTS), after 24 h incubation at concentrations up to 10 μM (Fig. 2c). This notable lack of acute toxicity suggests that **1** is a non-disruptive tool to assist analysis of NMT substrates in cells and is consistent with selective NMT inhibition, since the pre-existing pool of *N*-myristoylated proteins would be expected to maintain cell viability until depleted. In line with this hypothesis, a gradual and dose-dependent decrease in cell viability occurred over time in the presence of **1**. A plateau of metabolic activity was observed up to 10 μM **1**, and the level of this plateau diminished with extended duration of inhibition, with a consistent effector concentration for half-maximum response (EC_50_) of 210±15 nM. We hypothesize that this time-dependent response is characteristic of on-target, selective NMT inhibition in mammalian cells.

Treatment of HeLa cells with YnMyr (20 μM) and **1** (0–5 μM) for 24 h resulted in a robust dose-dependent decrease in labelling intensity for the major TAMRA-labelled bands, with maximal inhibition occurring by 5 μM **1** (Fig. 2d). Direct quantification of fluorescence intensity revealed an apparent in-cell ‘tagging IC_50_’ (hereafter referred to as TC_50_) of 170±31 nM (Fig. 2a), suggesting good uptake of this inhibitor in mammalian cells. Furthermore, enrichment and western blotting (WB) of YnMyr-tagged proteins revealed potent dose-dependent inhibition of *N*-myristoylation for several known NMT substrates, including c-Src tyrosine kinase (Fig. 2d). The TC_50_ value is closely similar to the EC_50_ noted above, and the plateau effect observed in the viability assay corresponds to inhibitor concentrations above which NMT is fully inhibited, providing additional evidence for on-target mode of action in cells. NMT-catalysed myristoylation generally occurs co-translationally during normal cell function, and so we further confirmed that **1** did not significantly affect protein synthesis in cells, which could in principle lead to an observed reduction in levels of myristoylated proteins. Protein labelling by azidohomoalanine, an established probe for protein synthesis^28^, was quantitatively blocked by the protein synthesis inhibitor cycloheximide, but was unaffected by 24 h treatment with **1** at a concentration >30-fold higher than TC_50_ (Fig. 2e). Finally, quantitative whole-proteome analysis of a total of 1,069 proteins following 24 h treatment with **1** confirmed that >96% show no significant (*P*<0.05) change in abundance, and >99.9% remained within twofold of the untreated control (Supplementary Fig. 4).

Taken together, these data demonstrate that **1** is a potent, protein substrate-competitive, dual NMT1/NMT2 inhibitor, and a highly selective pharmacological tool for inhibition of NMT activity in cells.

### Co-translational substrates and non-substrates of NMT

So-called ‘spike-in’ SILAC (stable isotope labelling by amino acids in cell culture) quantitative proteomics has recently emerged as a straightforward method to incorporate heavy isotope-labelled standards in a quantitative proteomics workflow^29^, and we considered that a combination of spike-in SILAC with YnMyr tagging and chemical NMT inhibition would provide a powerful platform for comprehensive analysis of NMT substrates in cells (Fig. 1b). This approach normalizes all samples across all sample-handling steps (CuAAC ligation, pull-down, sample preparation and nanoliquid chromatography-mass spectrometry (LC-MS)/MS), and does not require heavy isotope labelling of experimental samples, thus eliminating any potential disruption to the system under study. Accordingly, protein lysate from HeLa cells labelled with ^15^N_4_^13^C_6_-arginine and ^15^N_2_^13^C_6_-lysine (‘R10K8’) and tagged with YnMyr was used as an internal spike-in SILAC standard for proteins labelled in HeLa cells cultured in normal media in the presence of YnMyr (20 μM) and an increasing concentration of compound **1** (0–5 μM). Pull-down and on-bead tryptic digestion enabled quantification of proteome-wide changes in *N*-myristoylation in response to inhibition, determined in fivefold replicate experiments for each of six concentrations of **1** on a nanoLC-MS/MS platform (see below and Supplementary Methods for details of proteomic analyses).

**Figure 3:**
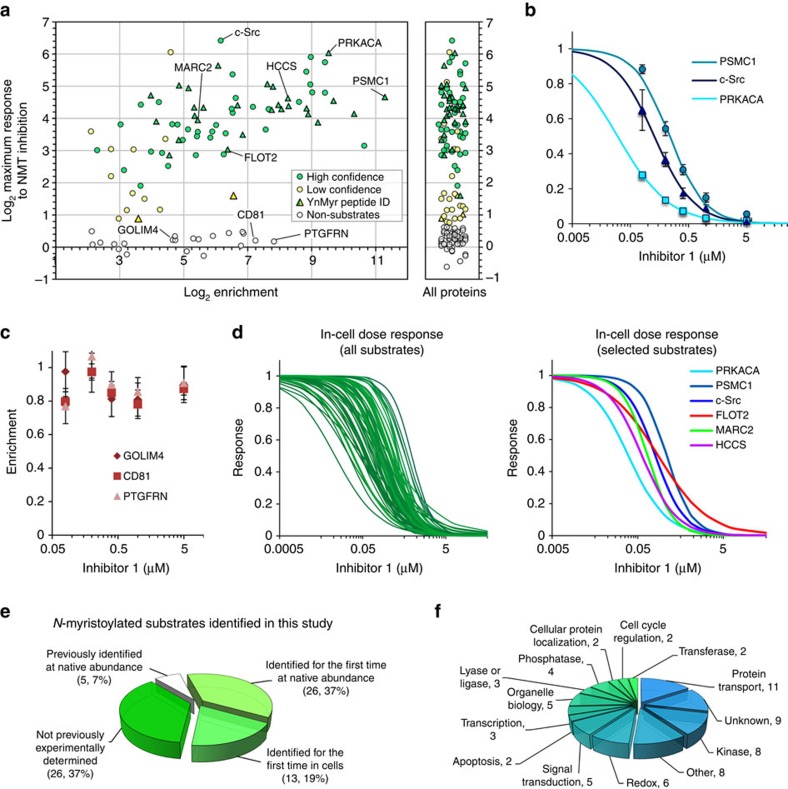
Identification of co-translational NMT substrates in HeLa cells. (**a**) Log_2_-fold change for maximal response of YnMyr enrichment of MG-initiated proteins to NMT inhibition (compound **1**, six concentrations, 0–5 μM, *n*=5) quantified by spike-in SILAC, plotted against enrichment relative to myristic acid fed control (by LFQ, see Supplementary Fig. 2). Circles plot substrates (green), substrates identified with lower confidence/fewer unique peptides (yellow) and non-substrates (grey circle); triangles indicate substrates for which a YnMyr-modified peptide was identified directly by MS/MS using AzRTB and/or AzKTB. Vertical scatter plot (at right) shows responses for all proteins identified, including those lacking quantifiable peptides in the myristate control. (**b**) Response of enrichment to NMT inhibition quantified by spike-in SILAC for the three known NMT substrates in Fig. 2d; error bars, s.d. (*n*=5). (**c**) Enrichment for three non-substrates shows no significant response up to the highest concentration of **1** tested; error bars, s.d. (*n*=5). (**d**) Dose-response curves for NMT substrates identified by quantitative chemical proteomics. Plots were generated by non-linear regression of IC_50_ values and slope factors to a standard dose-response equation (GraFit 7.0). Three known (PRKACA, c-Src and PSMC1) and three novel (FLOT2, MARC2 and HCCS) substrates are highlighted at the right. (**e**) Proportion of substrates identified here for the first time at their endogenous (native) expression level in live mammalian cells (green slices) vs those previously identified under the same conditions (white slice). (**f**) Biological functions for 70 NMT substrates identified in this study (Gene Ontology annotations).

More than 400 proteins without an N-terminal MG motif identified in these experiments showed no response to inhibition, as expected for non-substrates of NMT. For 169 enriched proteins carrying an N-terminal MG motif, 17,401 unique protein-derived peptide sequences were identified and quantified with high (>99.9%) confidence, resulting in 20,084 spike-in SILAC ratios for which the response to inhibition was analysed (Supplementary Data 2). These proteins partitioned into distinct populations of responding and non-responding proteins (Fig. 3a). Seventy proteins showed a robust response to inhibition and could thus be assigned with high confidence as substrates of NMT in cells, while a further 27 substrate proteins were assigned with lower confidence since analysis was based on a smaller set of unique peptides, due to the lower abundance of these proteins (Supplementary Data 2). Enrichment of substrate proteins exhibited a sigmoidal response to NMT inhibition (shown for three known NMT substrates in Fig. 3b) enabling an in-cell IC_50_ to be calculated per substrate, whereas that of non-substrates remained unaffected (Fig. 3c). The enrichment of 56 proteins bearing an N-terminal MG did not respond to NMT inhibition up to the highest concentration tested, and these proteins were therefore classified as non-substrates. The mean in-cell IC_50_ across NMT substrates was found to be 168±38 nM, closely comparable to the TC_50_ value from in-gel fluorescence, with an excellent fit to a standard dose-response curve for the large majority of substrates (mean slope factor=1.21±0.3) (Fig. 3d). The dose response for three examples of newly discovered substrates (HCCS, FLOT2 and MARC2) was probed by WB following pull-down, and all were found to follow the same trend determined by quantitative proteomic analyses (Supplementary Fig. 5). The inhibitor sensitivity of myristoylation of the large majority of substrates lies within 10-fold of the average in-cell IC_50_, with an overall range of sensitivity to inhibition of about 100-fold. We hypothesize that numerous factors may contribute to this interesting and subtle variation in substrate sensitivity observed in a cellular system, including subcellular localization, dynamic rate of synthesis in the cell, affinity for the enzyme-binding pocket, rate of leader Met removal by MetAP and complex interactions with the ribosome and potentially other proteins.

To provide further cross-validation of NMT substrates identified through this novel quantitative approach, we undertook profiling of the N-terminally modified peptides identified during chemical proteomic analysis using multifunctional reagents for capture of YnMyr-tagged proteins. Here we applied our previously reported reagent AzKTB^13^ and a novel reagent AzRTB, each of which incorporates a trypsin-cleavable site that enables release of the modified peptide with a hydrophilic Lys or Arg tag during protein digest for subsequent proteomic analysis (Supplementary Fig. 6). Applying these tools to the HeLa system above, we directly identified a total of 38 N-terminally YnMyr-modified peptides by MS/MS (Fig. 3a; Supplementary Fig. 7). The majority of peptides (27) were common to both reagents, with AzRTB providing identification of an additional 9 substrates over AzKTB (Supplementary Data 2); the improved performance of AzRTB may arise from enhanced on-bead proteolytic digestion, or superior chromatographic and mass spectral properties. Incomplete coverage of the tagged proteome through this approach is expected due to low detectability of certain modified peptides, for example, from sequence homology in the N-terminal tryptic peptide, the presence of multiple Lys or Arg residues proximal to the modification site, poor chromatographic or ionization profiles or abundance below the detection limit. Nevertheless, over 50% of substrates identified through quantitative proteomics (above) were directly validated by MS/MS sequencing of the N-terminally tagged peptide. Importantly, all 38 tagged sequences correspond to the N-terminal peptide of NMT substrates identified here, and there was no evidence for peptide modification within the non-substrate set (Fig. 3a; Supplementary Data 2), providing self-consistent validation of the approach.

Of the substrates of NMT identified here, <25% have previously been reported in experiments with chemically tagged myristate analogues (Supplementary Data 2 and Supplementary Table 1)^12^,^30^. We attribute this marked improvement to our new approach incorporating quantitative proteomics and selective inhibition, coupled to high-sensitivity mass spectrometry. Only five of the substrates identified here have previously been shown to be N-terminally myristoylated at an endogenous expression level in mammalian cells, for example, by immunoprecipitation (IP) of a substrate coupled to metabolic radiolabelling or mass spectrometry (Fig. 3e). Considering only the highest confidence assignments, 65 NMT substrates were identified here for the first time at endogenous abundance in live cells (that is, without overexpression of protein constructs), 39 for the first time in a cell-based context and 26 for the first time by any experimental approach. Very few bioinformatic predictions have been validated experimentally at native protein abundance in cells, and the two publicly available predictors disagree or conflict with prior evidence for *N*-myristoylation in several cases, particularly for lower confidence predictions (Supplementary Fig. 8)^18^,^19^,^31^. Classifying our NMT substrates into groups by prediction confidence (see Supplementary Methods), we found that the majority of substrate and non-substrate predictions were supported by experimental evidence (Fig. 3e). As expected, there was excellent (96%) accuracy for ‘high’ confidence predicted substrates, and the accuracy of predictions fell in line with confidence, with 17% ‘medium’ and 33% ‘low’ confidence predictions showing no response to NMT inhibition. A handful of ‘high’ confidence predictions were contradicted by evidence in cells (for example, GOLIM4, Fig. 3c), and 10% of non-substrate predictions were found to be inaccurate (for example, MARC2, FLOT2). These data offer the first comprehensive assessment of the *in vivo* accuracy of *in silico* NMT substrate predictions, and provide strong support for the accuracy of the confidence heuristics built into the most commonly used prediction tools. Previously determined estimates for the total number of distinct protein sequences expressed at a detectable level in HeLa cells (10,255)^32^ and the proportion of the human proteome predicted to be myristoylated (0.8%)^31^ suggest that about 80 proteins may be myristoylated in HeLa cells. This prediction is in good agreement with the total of 70 (at high confidence) to 97 (including lower confidence) substrates found here.

Taken together, these data provide the first proteome-wide determination of the substrates of NMT in a living system, and greatly expand the range of NMT substrates validated *in vivo*. A survey of functions by gene ontology annotation^33^ revealed distribution across a wide range of pathways in the cell (Fig. 3f), and an expected strong bias towards organelle and membrane localization (Supplementary Fig. 9).

### Profiling PTMyr during apoptosis

**Figure 4:**
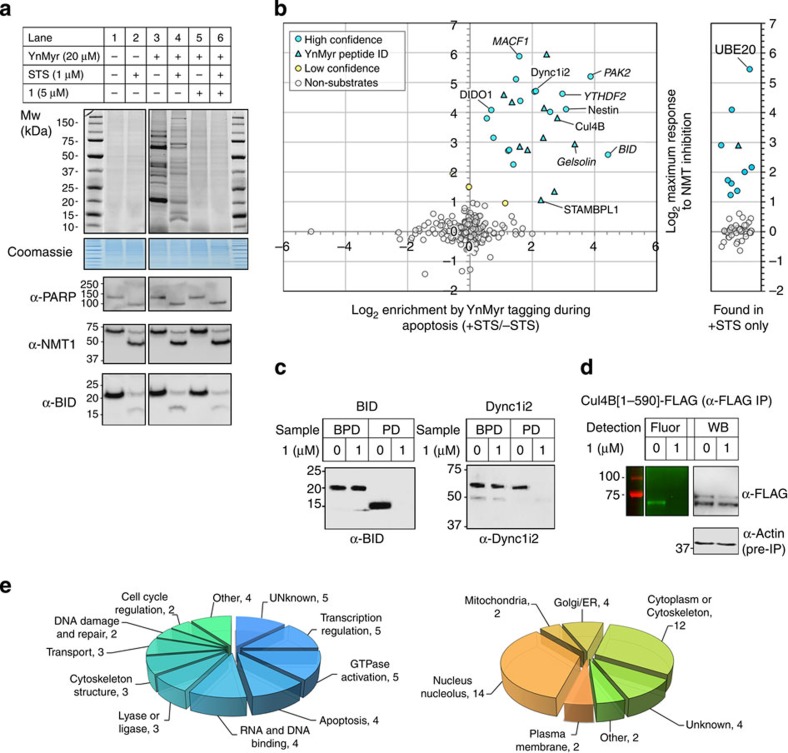
Identification of post-translational NMT substrates in HeLa cells during STS-induced apoptosis. (**a**) Cells were treated with STS, YnMyr and NMT inhibitor **1** (or DMSO vehicle) and proteins ligated (CuAAC) to AzTB and analysed by in-gel fluorescence (top), Coomassie staining (loading control) or western blots with antibodies against three markers of apoptosis, showing cleavage of PARP, NMT1 and BID (lower panels). Lanes marked (−) were treated with DMSO vehicle. STS does not affect background labelling (lanes 1–2), and NMT inhibition knocks down labelling in the presence or absence of STS (lanes 5–6). (**b**) Log_2_-fold change for maximal response of YnMyr enrichment of proteins to NMT inhibition during apoptosis (compound **1**, five concentrations 0–1 μM, each *n*=3) quantified by spike-in SILAC, plotted against median-centred enrichment relative to non-apoptotic control, quantified by SILAC. Circles plot post-translational substrates (blue), borderline substrates (yellow) and non-substrates (white/grey); triangles indicate substrates for which a YnMyr-modified peptide was identified directly by MS/MS. Plot at right shows inhibitor response for proteins found only following treatment with STS (that is, detected only under conditions of apoptosis). Selected protein IDs are indicated, with previously known substrates in italics. (**c**) Cells were treated with STS, YnMyr and NMT inhibitor **1** (or DMSO vehicle) and tagged proteins ligated (CuAAC) to AzTB and analysed by western blot against BID and Dync1i2 (BPD=before pull-down; PD=after pull-down on streptavidin-coated beads). Cleaved BID (ca. 15 kDa) or Dync1i2 (ca. 65 kDa) are enriched in an inhibitor-sensitive manner. (**d**) A C-terminal FLAG-tagged Culin 4B construct (Cul4B[1-590]-FLAG, Addgene) was overexpressed in HeLa cells for 17 h, whereupon the cells were treated with STS, YnMyr and NMT inhibitor **1** (or DMSO vehicle), as above. Proteins were immunoprecipitated (anti-FLAG), ligated (CuAAC) to AzT (azido-TAMRA) and analysed by in-gel fluorescence and by western blot; inhibitor-sensitive labelling of PTMyr Cul4B was observed at ca. 62 kDa; anti-actin western blot of IP supernatant provides loading control. (**e**) Biological functions and localization for the 40 post-translational substrates of NMT identified in this study (GO annotations).

*N*-myristoylation occurs mainly co-translationally during basal cell function; however, during apoptosis the protein BID (which mediates release of cytochrome *c* from mitochondria) has been shown to be post-translationally cleaved and *N*-myristoylated at endogenous expression levels^34^. There is also evidence for a small number of additional proteins under conditions of overexpression^35^ (Supplementary Table 2). The extent of PTMyr during apoptosis has not been systematically determined to date, and no reliable bioinformatic tool exists to predict PTMyr. Interestingly, NMT1 and NMT2 are also cleaved during apoptosis to generate new catalytically active species, which may also induce a change in substrate profile^36^. To determine the potential of our approach for profiling PTMyr during apoptosis, we first demonstrated apoptosis-dependent labelling of new *N*-myristoylated proteins in cells treated with staurosporine (STS), a broad spectrum kinase inhibitor that is a potent inducer of apoptosis^37^. Co-translational myristoylation was strongly downregulated on treatment with STS as judged by gel-based imaging, consistent with rapid inhibition of *de novo* protein synthesis. Furthermore, a new band pattern was observed that was readily inhibited by **1** without affecting robust induction of cleavage of markers of apoptosis (PARP, BID and NMT1) (Fig. 4a). SILAC-based quantification of YnMyr-tagged proteins further confirmed that labelling of most co-translationally *N*-myristoylated substrates is substantially reduced on STS treatment, while a subset of proteins that do not carry an N-terminal MG motif in the full-length protein are enriched (Supplementary Data 3). Combining our methodology for quantitative determination of sensitivity to NMT inhibition with STS-dependent enrichment, we identified 35 high-confidence and 5 low-confidence PTMyr proteins (Fig. 4b; Supplementary Data 4), including BID, four other proteins with prior evidence supporting PTMyr (Gelsolin, MACF1, PAK2 and YTHDF2) and 35 novel substrates. Using the optimized multifunctional reagent AzRTB, a total of 12 YnMyr-tagged peptides were identified from whole-proteome analysis and sequenced by MS/MS (Supplementary Fig. 10; Supplementary Data 4). All 12 modified peptides were found to contain an N-terminal YnMyr-glycine motif, and were derived from proteins in the high-confidence PTMyr data set (Fig. 4b), including numerous novel substrates. For each of these proteins, the site of cleavage and PTMyr was mapped to a specific glycine residue (Supplementary Fig. 11). Interestingly, all 12 modified sites are preceded by aspartate, suggesting apoptosis-induced cleavage by caspases, and the majority of the resulting new N termini are predicted as NMT substrates with medium or high confidence (Supplementary Data 4). BID^34^ and novel substrate cytoplasmic dynein 1 intermediate chain 2 (Dync1i2)^38^ were probed by WB following pull-down (Fig. 4c), confirming inhibitor-sensitive enrichment of an intact PTMyr substrate at native abundance. PTMyr was also confirmed in HeLa cells for another novel substrate, Culin 4B^39^, using a C-terminal FLAG-tagged construct (Cul4B[1-590]-FLAG) and an IP/on-resin ligation protocol^13^,^40^. YnMyr tagging was followed by IP with anti-FLAG, and CuAAC ligation to azido-TAMRA enabled inhibitor-sensitive fluorescence imaging of the cleaved product (Fig. 4d).

This first proteome-wide analysis of PTMyr in the context of apoptosis reveals a remarkable range of functions for novel PTMyr proteins, including ubiquitination, cytoskeleton remodelling and regulation of cell survival; more than half are localized to the nucleus or cytoskeleton prior to apoptosis (Fig. 4e).

## Discussion

Here we have found that simple chemical proteomic analysis based on metabolic labelling with tagged lipids has limited specificity, and results in identification of a large number of non-myristoylated proteins that may be due to a combination of metabolism, the lipid substrate promiscuity of protein *S*-acylation and incorporation into GPI anchors. However, we have shown that augmenting this analysis with sensitive quantitation of the impact of a selective NMT inhibitor allows robust differentiation of substrate and non-substrate proteins. Our data emphasize the importance of experimental determination of co- and post-translational myristoylation, and suggest that bioinformatic predictors of *N*-myristoylation may benefit from further refinement. The capacity to determine inhibition sensitivity of dozens of substrates simultaneously will be of particular interest in future drug discovery efforts aimed at targeting NMT for therapeutic benefit, for example, in cancer, and in assessing potential mechanism-based host toxicity of NMT inhibitors as drugs for infectious disease. The data obtained here suggest that the interplay between NMT inhibition and cell fate is likely to be complex, arising from potential loss of function in dozens of specific NMT substrate proteins, and changes in the function of many more PTMyr proteins on induction of apoptosis. However, our approach provides the first method to analyse and understand this complexity by enabling direct, multiplexed quantification of inhibitor response in live cells. Our studies reveal a range of sensitivity to NMT inhibition between substrates; as noted above, there are multiple possible explanations for this finding. It may be supposed that under normal circumstances NMT activity is not limiting, but on inhibition there may be increased competition between substrates for a substantially reduced pool of active NMT. However, we also note that the situation *in vivo* is complex and dynamic, and thus it is important to consider substrate sensitivity in living cells rather than in a simple enzyme–substrate complex formed *in vitro*. Previous studies suggest that NMT1 and NMT2 possess both distinct and overlapping biological roles. Comparison of the first crystal structure of NMT2 with that of NMT1 reveals a highly conserved active site, consistent with similar kinetic parameters in simple recombinant enzyme assays. We therefore hypothesize that any potential differences in substrate selectivity are driven by NMT–ribosome interactions, subcellular localization and other factors that can only be recapitulated in native, intact human cells. Future studies to identify NMT1- and NMT2-specific substrates could exploit our approach in combination with isozyme-specific knockdown.

Numerous important pathways involve proteins that are shown here for the first time to be co- or post-translationally *N*-myristoylated at their endogenous abundance in cells. A few notable co-translational examples in mitochondria include ligation of haem to cytochrome *c* (HCCS)^41^, activation of hydroxylated prodrugs and regulation of nitric oxide synthesis (mARC1, mARC2)^42^ and NADH dehydrogenation (NDUFAF4, NDUFB7)^43^. Among numerous novel targets involved in essential trafficking pathways, we discovered *N*-myristoylation of LAMTOR1 (ref. 44)^44^, which anchors the scaffolding complex of the mTORC1 and MAPK pathways, and Battenin/CLN3 (ref. 45)^45^, mutations of which can result in Batten disease, a fatal inherited neurodegenerative disorder. Novel substrates also include proteins that mediate nuclear processes; for example, nucleolar protein 3 (NOL3/ARC), which acts to inhibit apoptosis, has attracted much recent attention for its role in promoting tumorigenesis, metastasis and chemoresistance in a variety of cancers^46^, and as a mediator of pulmonary hypertension^47^. PTMyr substrates are equally diverse in function. They include components of the ubiquitination machinery such as STAMBPL1, a metalloprotease that cleaves Lys-63 polyubiquitin^48^, E2 ligase UBE2O^49^ and Cullin 4B^39^, a core member of E3 ligase complexes involved in cell cycle regulation and the response to ultraviolet-induced DNA damage. MACF1 (ref. 50)^50^ facilitates actin–microtubule interactions, while Dync1i2 (ref. 38)^38^ and CLASP2 (ref. 51)^51^ regulate microtubule dynamics during cell division. Among several regulators of cell survival, we identified death inducer-obliterator 1 (DIDO1), which is upregulated during apoptosis and implicated in melanoma progression^52^, and Nestin, an intermediate filament protein and putative cancer stem cell marker^53^. Taken together, these data indicate many potential novel roles for myristoylation that merit future investigation in both basal cell function and apoptosis, with significant implications for basic biology, and for drug development targeting NMT.

We have demonstrated here that a combination of sensitive, quantitative chemical proteomic analyses with a well-characterized, highly selective pharmacological tool can profile the substrates of co- and post-translational modification across the proteome, at native abundance in living cells. Furthermore, we have shown that this toolbox can be expanded using another chemical tool (STS) to profile post-translational modification events during apoptosis. Selective and cell-active chemical inhibitors and proteomic tools are available for an increasing range of enzymes and chemical modifications. Where these tools have been sufficiently well validated, we anticipate that our method will have wide future applicability for understanding modification profiles in living cells under native conditions.

## Methods

Synthetic procedures are reported in Supplementary Information.

### General methods

Culture media and reagents for HeLa cell culture were obtained from Sigma-Aldrich, Gibco (Life Technologies), Iris Biotech, A&E Scientific (PAA).

Fluorescence on the gel was recorded using an ETTAN DIGE Imager (GE Healthcare). Chemiluminescence was recorded using a LAS-3000 Imaging System (Fujifilm). Absorbance in 96-well plates was measured using a SpectraMax M2/M2e Microplate Reader from Molecular Devices.

For quantitative proteomics (spike-in SILAC and SILAC), R10K8 and R0K0 Dulbecco's modified Eagle's medium (DMEM) media were purchased from Dundee Cell Products. Cell dissociation buffer (enzyme free, phosphate-buffered saline (PBS) based), obtained from Gibco (Life technologies) was used instead of trypsin to detach the cells before passaging. Dialysed fetal bovine serum (FBS) was obtained from Sigma-Aldrich. For proteomics, all buffers were filtered using a 0.2-μM filter. Low-binding tubes (Protein LoBind tubes, Eppendorf) were used to carry out the enrichment of NMT substrates for MS-based proteomics and to freeze-dry the digested peptides. Plasmid encoding C-terminally FLAG tagged Cul4B (aa1-590) was obtained from Addgene, TurboFect transfection reagent was from Thermo Scientific, ANTI-FLAG M2 Affinity Gel was from Sigma-Aldrich. N_3_-TAMRA was purchased from Life Technologies.

### Enzymatic assay

Kinetic studies and IC_50_ values determinations were carried out using a 7-diethylamine-3-(4′maleimidylphenyl)-4-methylcoumarin fluorescence assay, as described by Goncalves *et al.*^14^ The full-length NMT1 and NMT2 enzymes were produced as described previously and used at a final concentration of 300 ng ml^−1^ (ref. 14). For kinetic studies, the initial rates are the mean of three initial rates determined with Wallac EnVison Manager Software. The data were fitted to the enzyme kinetics function in GraFit 7.0 (Erithacus Software Ltd, UK). IC_50_ values are the mean value of two or more determinations, and IC_50_ values were determined using GraFit 7.0 (Erithacus Software Ltd) by non-linear regression fitting.

### Protein production and X-ray crystallography

The catalytic domains of HsNMT isoforms 1 and 2 were produced as His-tagged proteins and purified as described previously^14^. Crystals of HsNMT1 (after removal of the purification tag with 3C protease) and HsNMT2 (with the N-terminal His-tag intact) were obtained by the vapour diffusion method. Binary complexes of HsNMT1 (6 mg ml^−1^ in 25 mM Tris–HCl pH 7.5, 100 mM NaCl, 1 mM dithiothreitol (DTT)) and Myr-CoA(5% volume added of 10 mM solution in 50% dimethyl sulphoxide (DMSO)) were crystallized by adding an equal volume of 22.5% polyethylene glycol (PEG) 4000, 5 mM NiCl_2_, 100 mM sodium citrate (pH 4.5) containing 2.5% glycerol as cryoprotectant. A ternary complex of HsNMT1 containing both co-factor and ligand **1** (added as 5% volume of a 10-mM solution in 50% DMSO) was crystallized using 25% PEG 2000 monomethyl ether, 0.2 M KBr, 100 mM sodium citrate (pH 4.5) containing 5% glycerol. HsNMT1 crystals belong to the orthorhombic space group P2_1_2_1_2 with two molecules of the complex in the asymmetric unit. HsNMT2 (7.5 mg ml^−1^ in 25 mM Tris pH 7.5, 100 mM NaCl, 1 mM DTT) was crystallized as a binary complex with the non-hydrolysable Myr-CoA analogue S-(2-oxo)pentadecyl-CoA (NHM)^54^ using 25% PEG 3350, 0.2 M KCl, sodium citrate (pH 5.5). HsNMT2 crystals belong to the orthorhombic space group P2_1_2_1_2_1_ with a single molecule of the complex in the asymmetric unit. For both crystal forms, the Matthews coefficient is 2.26 Å^3^/Da corresponding to a solvent content of 45%.

X-ray diffraction data were collected on synchrotron beam lines i02 (*λ*=0.9783 Å) and i03 (*λ*=0.9763 Å) at the diamond light source, Harwell and processed using XDS^55^ and SCALA^56^ implemented within *xia2*^57^. Data collection and refinement statistics are summarized in Table 1. For HsNMT2, data were used for molecular replacement calculations in the program MOLREP^58^ using a coordinate set corresponding to a single chain of HsNMT1 (PDB code 3IU1, 83% sequence identity) as a search model. For HsNMT1, the coordinates from PDB code 3IU1 were used directly for refinement using maximum likelihood methods implemented in REFMAC^59^. Cycles of refinement using anisotropic temperature factors were interspersed with model building and adjustment using COOT^60^.

Complete chains can be traced for both the proteins with the exception of N-terminal residues preceding Arg115 of NMT1 and Met109 of NMT2 (numbering as in full-length protein). These residues are not defined in the electron density maps and they are assumed to be disordered. The HsNMT1 protein structure models display good geometry, with 95% of the residues lying in the preferred regions of the Ramachandran plot and only a single residue (Phe422) lying outside of the allowed regions. Electron density maps in the vicinity of the bound ligand in the structure of HsNMT1-Myr-CoA-**1** are better defined for chain A, as reflected by the refined atomic B-factors (Table 1), and it is this active site that is displayed in the figures and referred to in the main text. For HsNMT2, the diffraction data showed a degree of anisotropy along the long axis of the crystal, contributing to the relatively high *R*-values during refinement. The lower-resolution maps are reflected in the poorer distribution of the modelled protein residues in a Ramachandran plot, with 92.9% in the preferred regions, a further 5.2% in the allowed region and seven residues calculated as outliers.

The coordinates and structure factor files have been deposited in the Protein Data Bank under the accession codes 4C2X (HsNMT2-NHM), 4C2Y (HsNMT1-Myr-CoA) and 4C2Z (HsNMT1-Myr-CoA-**1**).

### HeLa cell culture

HeLa cells (Cancer Research UK cell services core facility) were grown in DMEM (supplemented with 10% FBS and 1% penicillin/streptomycin) in a humidified 10% CO_2_-containing atmosphere at 37 °C. Cells were plated 24 h before treatments. For the spike-in SILAC and SILAC experiments, HeLa cells were grown in a R10K8 or R0K0 DMEM medium (Dundee Cell Products) supplemented with 10% dialysed FBS and 1% penicillin/streptomycin for >10 passages. The incorporation of the R10K8 label was determined to be >97%.

### YnMyr labelling and incubation with inhibitor in HeLa cells

Cells were incubated with inhibitor **1** (0, 0.08, 0.2, 0.4, 1 or 5 μM) for 30 min. The media of each plate was then removed and replaced by culture media containing the inhibitor and YnMyr (typically 20 μM). In each plate, the total amount of DMSO was normalized to the maximum amount of DMSO used. Cells were incubated for several hours (typically 24 h) before cell lysis. To study YnMyr labelling during apoptosis, cells were incubated for 30 min with STS (1 μM) and inhibitor **1** (0, 0.08, 0.2, 0.4 or 1 μM), and then YnMyr (20 μM) was added and the cells were incubated for a further 7 h before cell lysis. After treatment, cells were washed with PBS (2 × ) and lysed on ice using the following lysis buffer: PBS 1 × , 0.1% SDS, 1% Triton X-100, 1 × EDTA-free complete protease inhibitor (Roche Diagnostics). Lysates were kept on ice for 20 min and centrifuged at 17,000 *g* for 20 min to remove insoluble material. Supernatants were collected and stored at −80 °C. Protein concentration was determined using the Bio-Rad DC Protein Assay.

### Preparation of spike-in standards

To study co-translationally *N*-myristoylated proteins (CoTMyr), cells were grown in the heavy media (R10K8) and were treated for 24 h with 20 μM YnMyr (total amount of DMSO: 0.05%), and then lysed and treated as above. To prepare the Spike-in SILAC standard for the study of post-translationally *N-*myristoylated proteins (PTMyr), cells were grown in the heavy media (R10K8) as described above and were pre-treated for 30 min with STS (1 μM). Cells were then treated with YnMyr (20 μM) and STS (1 μM) for 7 h (total amount of DMSO: 0.05%).

### Preparation of YnMyr ±STS SILAC samples

To study YnMyr labelling during apoptosis, HeLa cells in R10K8 medium (heavy cells) were pre-incubated for 30 min with STS (1 μM), whereas cells in R0K0 medium (light cells) were treated for 30 min with DMSO. To both heavy and light cells was added YnMyr (20 μM) for 7 h.

### CuAAC and in-gel fluorescence

Lysates were thawed on ice. Proteins (100 μg) was taken and diluted to 1 mg ml^−1^ using the lysis buffer. A click mixture was prepared by adding reagents in the following order and by vortexing between the addition of each reagent: the capture reagent (1 μl, stock solution 10 mM in DMSO, final concentration 0.1 mM), CuSO_4_ (2 μl, stock solution 50 mM in DMSO, final concentration 1 mM), tris(2-carboxyethyl)phosphine (TCEP) (2 μl, stock solution 50 mM in DMSO, final concentration 1 mM), tris(benzyltriazolylmethyl)amine (TBTA) (1 μl, stock solution 10 mM in DMSO, final concentration 0.1 mM). Click mixture (6 μl) was added to each sample. The samples were vortexed at room temperature (RT) for 1 h. Next, 1 ml ice-cold MeOH and EDTA (final concentration 10 mM) were added to each sample. The samples were quickly vortexed and kept at −80 °C overnight. The samples were centrifuged at 17,000 *g* for 30 min to pellet precipitated proteins. The pellets were washed with 1 ml ice-cold MeOH and dried. Seventy-five microlitre 2% SDS in PBS, 10 mM EDTA was added and the samples were vortexed. Once the proteins were completely dissolved, 25 μl of 4 × SLB (sample-loading buffer prepared by mixing 4 × NuPAGE LDS sample buffer: β-mercaptoethanol 5:1) was added (final concentration of proteins 1 mg ml^−1^). The samples were boiled for 5 min, centrifuged at 1,000 *g* for 2 min and loaded on a SDS–polyacrylamide gel electrophoresis gel. Typically, 10 μg of proteins were loaded on the gel. After the run, the gel was washed with MilliQ (3 × ) and soaked in fixing solution (40% MeOH, 10% acetic acid, 50% water) for 20 min, washed with MilliQ (3 × ). The fluorescence on the gel was detected using an Ettan DIGE Imager (GE Healthcare) and the protein loading was checked by Coomassie. The quantification of fluorescent bands to calculate in-cell tagging IC_50_ was carried out using ImageJ. The signal was measured by integrating the area under each band of interest using the ‘gel analyzer’ function and normalized relative to no inhibition (YnMyr). The signal was normalized to the total protein loading by integrating the gel stained with Coomassie. In-cell tagging IC_50s_ (TC_50s_) were determined using GraFit 7.0 (Erithacus Software Ltd) by non-linear regression fitting.

### *N*-myristoylated protein enrichment and in-gel fluorescence

For enrichment of myristoylated proteins, 100 μl of lysate was captured by CuAAC as described above. After protein precipitation, the pellet was resuspended in 2% SDS in PBS, 10 mM EDTA (20 μl). Once the pellet was completely dissolved, 80 μl of PBS was added (final concentration 1 mg ml^−1^, 0.4% SDS). Twenty microlitre was reserved for the ‘before pull-down’ sample. The remaining 80 μl was diluted to 0.2% SDS and 0.5 mg ml^−1^ by the addition of 80 μl PBS. Dynabeads MyOne Streptavidin C1 (15 μl) were washed with 0.2% SDS in PBS (3 × 250 μl). The sample was added to the beads and the beads were gently vortexed for 90 min. The supernatant was removed and the beads were washed with 0.2% SDS (3 × 250 μl). Thirty microlitre of 2% SDS in PBS and 10 μl 4 × SLB were added to the beads and the beads were boiled for 10 min. SLB (7 μl) was added to the ‘before pull-down’ sample and 7 μl SLB was added to 20 μl of supernatant. Both samples were boiled for 5 min. Samples were centrifuged at 1,000 *g* for 2 min and loaded on a SDS–polyacrylamide gel electrophoresis gel (‘before pull-down’ sample: 10 μl (7.5 μg of proteins); supernatant: 10 μl (7.5 μg of proteins); pull-down: 15 μl (30 μg of proteins). Full size original images for all cropped gels can be found in Supplementary Fig. 12.

### Western Blot analysis

Proteins were transferred to polyvinylidene difluoride membranes using a wet transfer setup. Membranes were washed with TBS-T (1 × TBS, 0.1% Tween-20), blocked (5% dried skimmed milk in TBS-T), washed with TBS-T (3 × ), incubated with the appropriate primary antibody (c-SRC (Cell signaling technology, 2123, 1/500 dilution); PMSC1 (Atlas, HPA000872, 1/500 dilution); PKA (Cell Signaling Technology, 5842S, 1/500 dilution), FLOT2 (Atlas antibodies, HPA002946, 1/500 dilution), MARC2 (Atlas antibodies, HPA015085, 1/150 dilution), HCCS (Atlas antibodies, HPA002946, 1/500), DYNC1I2 (Abcam, ab96288, 1/250 dilution), NMT1 (Atlas antibodies, HPA022963, 1/500 dilution), PARP (Santa Cruz, sc8007, 1/250 dilution), BID (Cell Signaling, 2002S, 1/250), FLAG (Abcam, ab1162, 1/1000 dilution) and Actin (Abcam, ab3280, 1/1,000 dilution) in blocking solution overnight, washed with TBS-T (4 × ), incubated with the appropriate secondary antibodies in blocking solution for 1 h, washed with TBS-T (4 × ) and developed with Luminata Crescendo Western HRP substrate (Millipore) according to the supplier’s protocol and on a Fujifilm LAS-3000 imager. Full size original images for all cropped blots can be found in Supplementary Fig. 12.

### CuAAC and sample preparation for MS-based proteomics

CuAAC reaction was carried out with 2 mg ml^−1^ of proteins (CoTMyr) and 0.6 mg ml^−1^ (PTMyr). The spike-in standard was mixed with the lysates treated with increasing amount of inhibitor and YnMyr in a 1:2 (CoTMyr) or 1:3 (PTMyr) ratio of lysate to spike-in standard, before the CuAAC reaction. The same spike-in standard was used for all the samples belonging to a specific set (CoTMyr or PTMyr) to allow the comparison between samples within this set. Proteins were captured by adding a mixture of the capture reagent AzTB (final concentration 0.1 mM), CuSO_4_ (final concentration 1 mM), TCEP (final concentration 1 mM) and TBTA (final concentration 0.1 mM). The samples were vortex-mixed for 1 h before the addition of EDTA (final concentration 10 mM), methanol (4 volumes), chloroform (1 volume) and water (3 volumes). The samples were vortex-mixed briefly and centrifuged at 10,000 *g* for 5 min, and the resulting pellets were washed twice with methanol (4 volumes) and dried. After protein precipitation, the pellet was resuspended in 2% SDS in PBS, 10 mM EDTA (10 mg ml^−1^ of proteins). Once the pellet was completely dissolved, PBS was added to dilute the sample (final concentration 1 mg ml^−1^, 0.2% SDS). NeutrAvidin agarose resin (Thermo Scientific) was washed with 0.2% SDS in PBS (3 × ). Typically 50 μl of bead slurry was used for 1 mg of lysate. The sample was added to the beads and the enrichment was carried out for 2 h at RT. The supernatant was removed and the beads were washed with 1% SDS in PBS (3 × ), 4 M urea in 50 mM ammonium bicarbonate, AMBIC (3 × ), 50 mM AMBIC (5 × ). For a 50 μl bed of beads, samples were reduced (2.5 μl of 100 mM DTT in 50 mM AMBIC) at 60 °C for 30 min and allowed to cool to RT. The beads were washed with 2 × 50 mM AMBIC. Cysteines were alkylated (2.5 μl of 100 mM iodoacetamide in 50 mM AMBIC) at RT for 30 min in the dark. The beads were washed with 2 × 50 mM AMBIC. Trypsin (2 μg Sequencing Grade Modified Trypsin (Promega) dissolved in 50 mM AMBIC) was added to the beads and the samples were placed on a shaker and digested overnight at 37 °C. The samples were centrifuged and the supernatant (SN1) was transferred into clean tubes. The beads were washed with 0.1% aqueous formic acid, and these washes (SN2) were combined with SN1 fractions. The solutions were stage-tipped according to a published protocol^61^. Elution from the sorbent (SDB-XC poly(styrenedivinylbenzene) copolymer, from 3 M) with 70% acetonitrile in water was followed by speed-vac-assisted solvent removal, reconstitution of peptides in 0.5% trifluoroacetic acid, 2% acetonitrile in water and transferred into LC-MS sample vials.

### LC-MS/MS analysis

The analysis was performed using an Acclaim PepMap RSLC column 50 cm × 75 μm inner diameter (Thermo Fisher Scientific) using a 2-h acetonitrile gradient in 0.1% aqueous formic acid at a flow rate of 250 nl min^−1^. Easy nLC-1000 was coupled to a Q Exactive mass spectrometer via an easy-spray source (all Thermo Fisher Scientific). The Q Exactive was operated in data-dependent mode with survey scans acquired at a resolution of 75,000 at *m*/*z* 200 (transient time 256 ms). Up to 10 of the most abundant isotope patterns with charge +2 or higher from the survey scan were selected with an isolation window of 3.0 *m*/*z* and fragmented by higher-energy collision dissociation (HCD) with normalized collision energies of 25. The maximum ion injection times for the survey scan and the MS/MS scans (acquired with a resolution of 17,500 at *m*/*z* 200) were 20 and 120 ms, respectively. The ion target value for MS was set to 10^6^ and for MS/MS to 10^5^, and the intensity threshold was set to 8.3 × 10^2^.

### Proteomics data analysis

The data were processed with MaxQuant version 1.3.0.5 (ref. 62)^62^, and the peptides were identified from the MS/MS spectra searched against human Swissprot+Isoforms database (July 2013) using the Andromeda search engine. Cysteine carbamidomethylation was used as a fixed modification and methionine oxidation as a variable modification. For *in silico* digests of the reference proteome, the following peptide bond cleavages were allowed: arginine or lysine followed by any amino acid (a general setting referred to as trypsin/P); arginine or lysine followed by any amino acid as well as any amino acid followed by glycine (this setting was applied specifically for PTMyr-modified peptide searches). Up to two missed cleavages were allowed. The FDR was set to 0.01 for peptides, proteins and sites. Other parameters were used as pre-set in the software. ‘Unique and razor peptides’ mode was selected to allow for protein grouping; this calculates ratios from unique and razor peptides (razor peptides are uniquely assigned to protein groups and not to individual proteins). Data were analysed using Microsoft Office Excel 2007, Perseus version 1.3.0.4, Origin 8.5 and GraFit 7.0.

### Label-free quantification data analysis

LFQ experiments in MaxQuant were performed using the built-in LFQ algorithm^63^ enabling the ‘match between runs’ option (time window 2 min), and were processed with Perseus version 1.3.0.4. The experiment comprised four biological replicates treated with Myr and four biological replicates treated with YnMyr. The LFQ is based on intensities of proteins calculated by MaxQuant from peak intensities and based on the ion currents carried by several peptides whose sequences match a specific protein or a protein group to provide an approximation of abundance. The replicates were grouped together. The YnMyr protein group was filtered to obtain at least three valid values across the four replicates and in addition at least two peptides (unique and razor) per valid value. Label-free intensities were logarithmized (base 2) and empty values were imputed with random numbers from a normal distribution, whose mean and s.d. were chosen to best simulate low abundance values close to noise level (impute criteria: width 0.1 and down shift 1.8). A modified *t*‐test with permutation-based FDR statistics was applied (250 permutations; FDR=0.001; S0=1). The data were further filtered by keeping only the proteins with an LFQ enrichment higher than 4. The LFQ enrichment was determined by first calculating the mean value of the LFQ intensities of the Myr replicates and the mean value of the LFQ intensities of the YnMyr replicates. The mean of YnMyr intensities was then divided by the mean of Myr intensities.

### Spike-in SILAC (CoTMyr) data analysis

Light/heavy ratios, corresponding to the amount of protein in the lysate with increasing amount of inhibitor in relation to the spike-in standard, found for each protein, each condition (concentration of inhibitor) and each replicate (five biological replicates) were determined by MaxQuant as explained above. The data were filtered to keep only proteins with a N-terminal MG motif and three valid values per protein. For each replicate, the values were normalized to the sample with 0 μM inhibitor **1**. To compare the six conditions (six concentrations of inhibitor), an analysis of variance was performed with Benjamini Hochberg correction at an FDR of 0.05. In addition, hierarchical clustering was performed in Perseus, where proteins sensitive to NMT inhibition were clustered together while the insensitive proteins were in different clusters. The data were then manually inspected. Proteins that were dose-dependently sensitive to NMT inhibition, with a minimal ratio <0.3, were defined as ‘*N*-myristoylated with high confidence’. In-cell IC_50_ values were determined using the mean values of the ratios between the five replicates and determined using GraFit 7.0 (Erithacus Software Ltd) by non-linear regression fitting (IC_50_ back-corrected function). When proteins had a low number of unique peptide (0 or 1 in the highest concentrations of inhibitors), they were classified as low-confidence hits. Proteins with an ambiguous dose response (lowest ratios do not drop <0.3 but drop <0.6) were classified as ‘*N*-myristoylated with low confidence’. Other proteins were classified as ‘not *N*-myristoylated with high/low confidence’.

### SILAC experiment (YnMyr ±STS) data analysis

The heavy samples originated from cells treated with YnMyr+STS and the light samples originated from cells treated with YnMyr. Ratios H/L (heavy/light) were determined for each protein in three biological replicates (cells harvested from separate plates) with MaxQuant 1.3.0.5 following LC-MS/MS analyses. During the analysis in Perseus, only proteins quantified in at least two biological replicates (two valid values out of three) were kept. The ratios H/L were divided by 3, as the samples were mixed after cell lysis in a 3:1 ratio heavy to light. The Log2 values of the ratios were determined and a one-sample test (*t*-test, FRD=0.05, side: left), where the differences from the median H/L distributions were analysed, was performed.

### Identification of the modified peptide with AzKTB or AzRTB

MaxQuant utilizes a scoring algorithm when matching experimental MS/MS spectra with a library of theoretical spectra generated from *in silico* digestion of proteins within databases selected for the search^62^,^64^,^65^. The algorithm is used to evaluate the quality of peptide–spectrum matches (PSMs). A number of factors, such as the number of product ions detected or their intensities, contribute to the score. To each PSM, MaxQuant also attributes a Δscore, which is a difference between scores associated with the match to the best peptide candidate and the second best match within the database; the higher the score and the Δscore, the more reliable the identification. MaxQuant utilizes a predefined FDR value (0.01 for all searches) to generate score thresholds separately for different populations of peptides, for example, different peptide length, nature and number of modifications and so on. To reduce a possibility for a false-peptide sequence assignment even further, Δscore thresholds were additionally applied (20 for AzRTB and 40 for AzKTB) in our analysis. See Supplementary Data 2 and 4 for the per-protein Δscore, and Supplementary Figs 7 and 10 for examples of sequence analysis by MS/MS assignments.

### Spike-in SILAC (PTMyr) data analysis

Ratios light/heavy (corresponding to amount of protein in the lysate with increasing amount of inhibitor/spike-in standard) found for each protein, each condition (concentration of inhibitor) and each replicate (*n*=3 biological replicates) were determined by MaxQuant 1.3.0.5 following LC-MS/MS analyses. The data were filtered to obtain at least three valid values per protein. For each replicate, the values were normalized to the sample with 0 μM of inhibitor, thus the amount of protein in the 0 μM of inhibitor samples was set as 1. To compare the five conditions (five concentrations of inhibitor), an analysis of variance was performed with Benjamini Hochberg correction (FDR=0.05). Co-translationally *N*-myristoylated proteins were also tagged with YnMyr, but they were excluded (according to the identified substrates in Supplementary Data 2). The data were then manually inspected. Proteins were defined as substrates when their mean ratio was <0.5 in the sample with the highest concentration of inhibitor, for which quantification was available. Proteins that were not enriched in the STS-treated sample (mean ratio ~1.0, see Supplementary Data 4), but were sensitive to the presence of inhibitor 1, were classified as post-translationally myristoylated-low confidence. The unique peptides detected for each protein were mapped to the protein sequences (see Supplementary Fig. 5). The cleavage site was predicted using the CASVM server (see Bioinformatics).

### Whole-lysate ± inhibitor data analysis

The experiment comprised three biological replicates for each sample (0 day treatment, 1 day treatment) spiked-in with heavy HeLa cells not treated with the inhibitor. Ratios H/L (corresponding to the amount of protein in the spike-in standard/in the lysate treated with the inhibitor for 0–1 day) found for each protein, each condition and each replicate (three biological replicates) were determined by MaxQuant as explained above. The replicates were grouped together. The group were filtered to obtain at least two valid values. Ratios were logarithmized (base 2). Modified *t*-tests with permutation-based FDR statistics were applied (250 permutation, 5%, two tailed, s0=0) between the two groups. Data output can be found in Supplementary Data 5.

### Bioinformatics

Myristoylation predictions were carried out using the two online predictors (Myr predictor^19^ and Myristoylator^18^). The Myr predictor divides proteins into groups with three levels of confidence: R=reliable; T=twilight, N=not myristoylated, and the myristoylator uses four levels of confidence: H=high probability, M=medium probability, L=low probability, N=not myristoylated. Four levels of myristoylation confidence were defined by combining the results of the two predictors: N=not myristoylated, P=probable, L=low, M=medium, H=high. Proteins with probability *R* (Myr predictor) and *H* (Myristoylator), were reported to be myristoylated with a high probability (*H*). Proteins with probability *R* (Myr predictor) and *M* (Myristoylator) were reported to be myristoylated with a medium probability (*M*).

In the present study, proteins were described as palmitoylated when they were identified as palmitoylated in at least one proteomic experiment, as reported by Wilson *et al.*^12^ (+: found in one study, ++: found in two studies; +++: found in three studies; ++++: found in four studies), or reported as palmitoylated in the UniProtKB/Swiss-Prot database (indicated as u in the data set). GPI-anchored proteins are reported as found in the UniProtKB/Swiss-Prot database.

To predict proteolysis cleavage sites, the CASVM server was used ( http://www.casbase.org/casvm/server/index.html) with the following options: scanning window size of P14-P10′ and P1 residue aspartic or glutamic acid. The cleavage site was correlated with the PROTOMAP database ( http://www.scripps.edu/cravatt/protomap/) and the CASBAH database^66^ ( http://bioinf.gen.tcd.ie/casbah/).

### MTS assay

HeLa cells were seeded in a 96-well plate 24 h before treatment. Cell suspensions were prepared in the appropriate growth media using the following concentrations: 100,000 cells ml^−1^ for 24 h treatment, 50,000 cells ml^−1^ for 48 h treatment, 32,000 cells ml^−1^ for 72 h or 10,000 cells ml^−1^ for 1 week treatment. Fifty microlitre of cell suspension was transferred to wells B–G in columns 2–11 of a 96-well plate. Growth media (100 μl) was added to the outer wells. Twenty-four hours after seeding, 50 μl of growth media containing DMSO (positive control; maximum amount of DMSO used for the inhibitor dilutions, 0.04%) or puromycin (negative control; 4 μg ml^−1^, 50 μl, final concentration in the plate 2 μg ml^−1^) or different concentrations of inhibitor were prepared (same final percentage of DMSO; dilution factor=2; typically triple dilutions starting from 20 μM; final concentration in the plate starting from 10 μM) were added to the cells. Twenty-four, 48, 72 or 164 h later, MTS reagent (Promega) was added according to the supplier’s protocol. Absorbance was measured at 490 nm after 2 h. The average of absorbance values of the negative control was subtracted from every value. The metabolic activity was calculated as a percentage relative to the positive control. EC_50_ values were calculated by fitting the data to the IC_50_ function using GraFit 7.0 (Erithacus Software Ltd).

### Overexpression and analysis of Cul4B[1-590]-FLAG

HeLa cells (4 × 10^5^) were reverse transfected with plasmid encoding for Cul4B (aa 1–590) utilizing TurboFect transfection reagent (17 h) according to the manufacturer’s instructions. Next, the media was changed followed by treatment with STS (1 μM), NMT inhibitor **1** (1 μM) or vehicle (DMSO). After 1 h incubation, the cells were treated with YnC12 (20 μM, 6 h) and then lysed (PBS, 1% Triton, protein inhibitor). Total proteins (50 μg) was applied onto the washed Anti-FLAG affinity resin (40 μl of 50% slurry) and incubated on a rotation wheel at 4 °C for 2 h. Following the IP step, the supernatant was removed and beads washed (3 × , lysis buffer). Next, click reaction with N_3_-TAMRA was performed on beads utilizing the standard conditions (as above) and excess fluorophore was removed by washing (as above). The beads were then boiled in 2% SDS/PBS (25 μl) at 95 °C for 10 min. The supernatant was removed and 15 μl was loaded on a gel. Pull-downed proteins were visualized by in-gel fluorescence followed by WB (α-FLAG). The supernatant from the IP step was utilized as loading control for WB. Proteins were precipitated (MeOH/CHCl_3_) and resuspended in 2% SDS/PBS (50 μl, 1 mg ml^−1^). Total proteins (10 μg) were loaded on a gel and analysed by α-actin WB.

## Author contributions

E.T., R.A.S., M.B., M.H.W., D.J.M. and E.W.T. designed the experiments. E.T. performed experiments related to co- and post-translational myristoylation and analysed data. R.A.S., M.B. and M.H.W. performed experiments for post-translational myristoylation and analysed data. M.B. synthesized the reagents AzKTB and AzRTB and R.A.S. and M.B. performed proteomic identification of modified peptides. U.B. and W.P.H. contributed to initial tool and methodology development. J.A.B. and A.J.W. performed X-ray crystallographic analyses and generated recombinant proteins. E.W.T. conceived and directed the study, and E.T., R.A.S. and E.W.T. wrote the manuscript with input from all authors.

## Additional information

**Accession codes.** Primary accessions (Protein Data Bank): 4C2Y (NMT1-NHMCoA); 4C2X (NMT2-NHMCoA); 4C2Z (NMT1-Myr-CoA-1).

**How to cite this article:** Thinon, E. *et al.* Global profiling of co- and post-translationally *N*-myristoylated proteomes in human cells. *Nat. Commun.* 5:4919 doi: 10.1038/ncomms5919 (2014).

## Supplementary Material

Supplementary Figures, Tables, Methods and References Supplementary Figures 1-12, Supplementary Tables 1-2, Supplementary Methods and Supplementary References

Supplementary Data 1 Label-free quantification of YnMyr-tagged proteins in HeLa

Supplementary Data 2 Spike-in experiment with increasing amount of inhibitor 1

Supplementary Data 3 SILAC YnMyr-tagged proteins in HeLa during apoptosis

Supplementary Data 4 Spike-in experiment with increasing amount of inhibitor 1 in HeLa cells undergoing apoptosis

Supplementary Data 5 Whole lysate quantification after 1 day inhibition

## Figures and Tables

**Table 1:** X-ray data collection and refinement statistics.

	**HsNMT2-NHM**	**HsNMT1-Myr-CoA**	**HsNMT1-Myr-CoA-1**
PDB accession code	4C2X	4C2Y	4C2Z
*Data collection*			
Cell dimensions (Å) *a*, *b*, *c*	49.70, 72.32, 114.69	78.74, 178.48, 58.55	78.63, 179.12, 58.59
Space group	*P*2_1_2_1_2_1_	*P*2_1_2_1_2	*P*2_1_2_1_2
Resolution (Å)*	57–2.33 (2.41–2.33)*	72–1.64 (1.67–1.64)	89–2.08 (2.14–2.08)
*R*_sym_ (%)	6.1 (64.4)	7.0 (70.6)	15.0 (68.6)
*I*/*σI*	15.2 (2.8)	17.7 (2.4)	12.9 (3.4)
Completeness (%)	99.9 (99.9)	98.2 (85.0)	100 (100)
Redundancy	6.3 (6.6)	7.1 (6.0)	7.1 (7.1)
CC_1/2_†	1.0 (0.84)	1.0 (0.75)	1.0 (0.85)
			
*Refinement*			
No. of unique reflections	18,414	98,990	50,468
*R*_work_/*R*_free_	21.8/28.8	17.0/22.1	16.9/23.8
No. of atoms	3,345	7,569	7,221
Protein	3,212	6,571	6,434
Ligand	NA	NA	64
Co-factor	64	126	126
Water	68	802	537
B-factors (Å^2^)			
All atoms	54.4	22.0	25.9
Protein	54.5	20.9	25.3
Ligand	NA	NA	41.2/78.6‡
Co-factor	45.2	15.7	18.7
Water	49.5	30.0	29.9
r.m.s.d.§			
Bond lengths (Å)	0.013	0.021	0.023
Bond angles (°)	1.672	2.230	2.283

NA, not applicable; PDB, protein database.

^*^Highest resolution shell is shown in parentheses.

^†^Pearson correlation coefficient between two random half-data sets^68^.

^‡^B-factor for ligand in chain A/chain B.

^§^r.m.s.d. of bond lengths or bond angles from ideal geometry.
