# Supplementary material for: Global profiling of co- and post-translationally N-myristoylated proteomes in human cells
Source: Nat Commun. 2014 Sep 26;5:4919. doi: 10.1038/ncomms5919 (PMC4200515; doi:10.1038/ncomms5919)
Supplement: Supplementary Figures, Tables, Methods and References — Supplementary Figures 1-12, Supplementary Tables 1-2, Supplementary Methods and Supplementary References [file ncomms5919-s1.pdf]

a

	*K*m c-Src 8mer peptide		*K*m Myr-CoA	*K*m YnMyr-CoA
	With Myr-CoA	With YnMyr-CoA		
NMT1	3.8 ± 0.8 (V_{max} = 26389 ± 1725)	9.8 ± 1.6 (V_{max} = 22953 ± 1480)	4.1 ± 1.1 (V_{max} = 30795 ± 2537)	0.7 ± 0.3 (V_{max} = 17383 ± 1210)
NMT2	1.1 ± 0.2 (V_{max} =15591 ± 650)	2.0 ± 0.6 (V_{max} = 17586 ± 1319)	2.9 ± 0.9 (V_{max} = 19487± 1735)	1.2 ± 0.1 (V_{max} = 15888 ± 373)

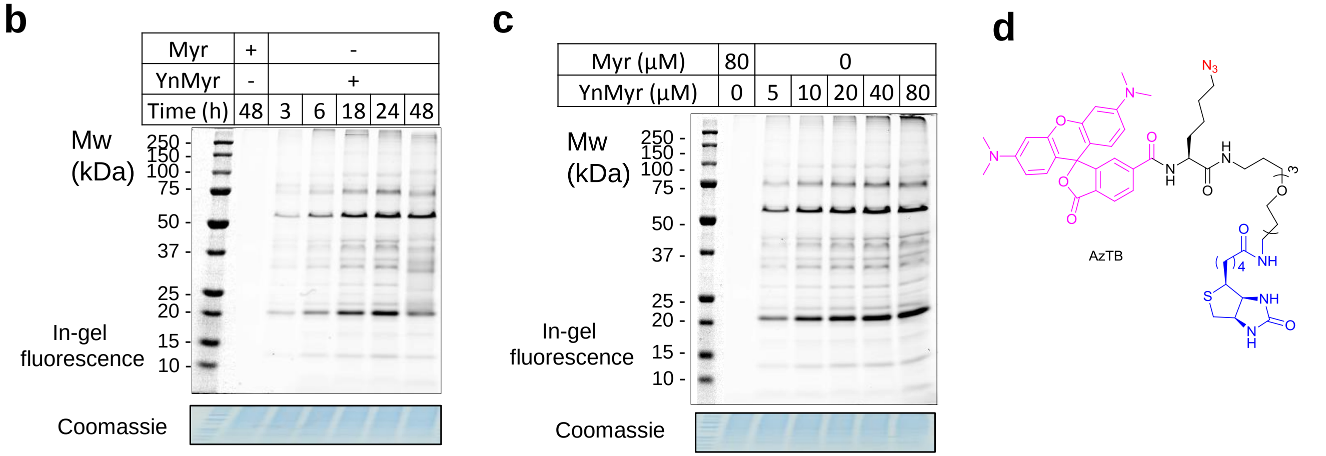

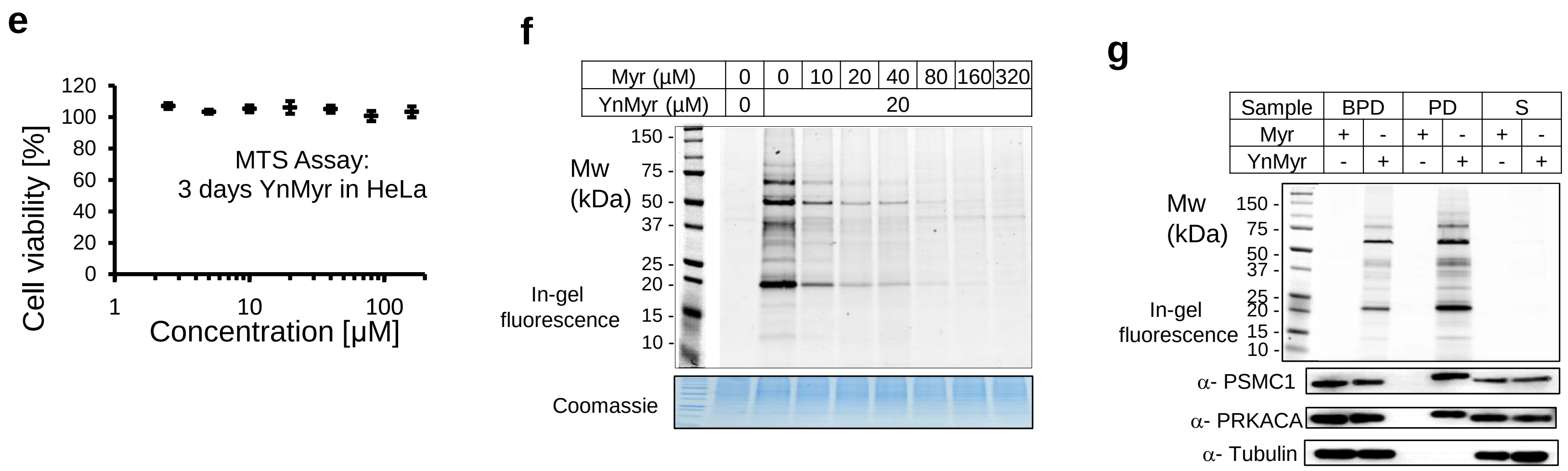

Supplementary Figure 1: YnMyr labeling in mammalian cells. a) K_m of 8AA N-terminal c-SRC was determined in the presence of NMT1 or NMT2 and in the presence of Myr-CoA or YnMyr-CoA using a fluorogenic assay [1]. The data was fitted to the Michaelis–Menten equation using GraFit (7.0). The K_m of Myr-CoA and YnMyr-CoA were also determined in presence of 8AA N-terminal c-Src and NMT1/2; errors, s.e.m. *(n*= 3) b) Time-course labeling with YnMyr (20 µM) in HeLa. c) Concentration series of YnMyr in HeLa. Cells were labeled for 24 h. d) Structure of the capture reagent AzTB (azido-tamra-biotin) [2]. e) Cell viability assay (MTS assay (Promega)) for 72 h in HeLa cells treated with increasing amount of YnMyr; errors s.d. (n = 3). f) Competition experiment: YnMyr was shown to compete with the natural lipid substrate (Myr). g) Samples were analyzed by in-gel fluorescence (top) or enriched by pull-down on streptavidin beads and analyzed by Western blot (WB; bottom). The sample before pull-down (BPD), pull-down sample (PD) and the supernatant from the pull-down (S) were analyzed. PSMC1 and PRKACA were enriched in the pull-down samples. Tubulin: loading control.

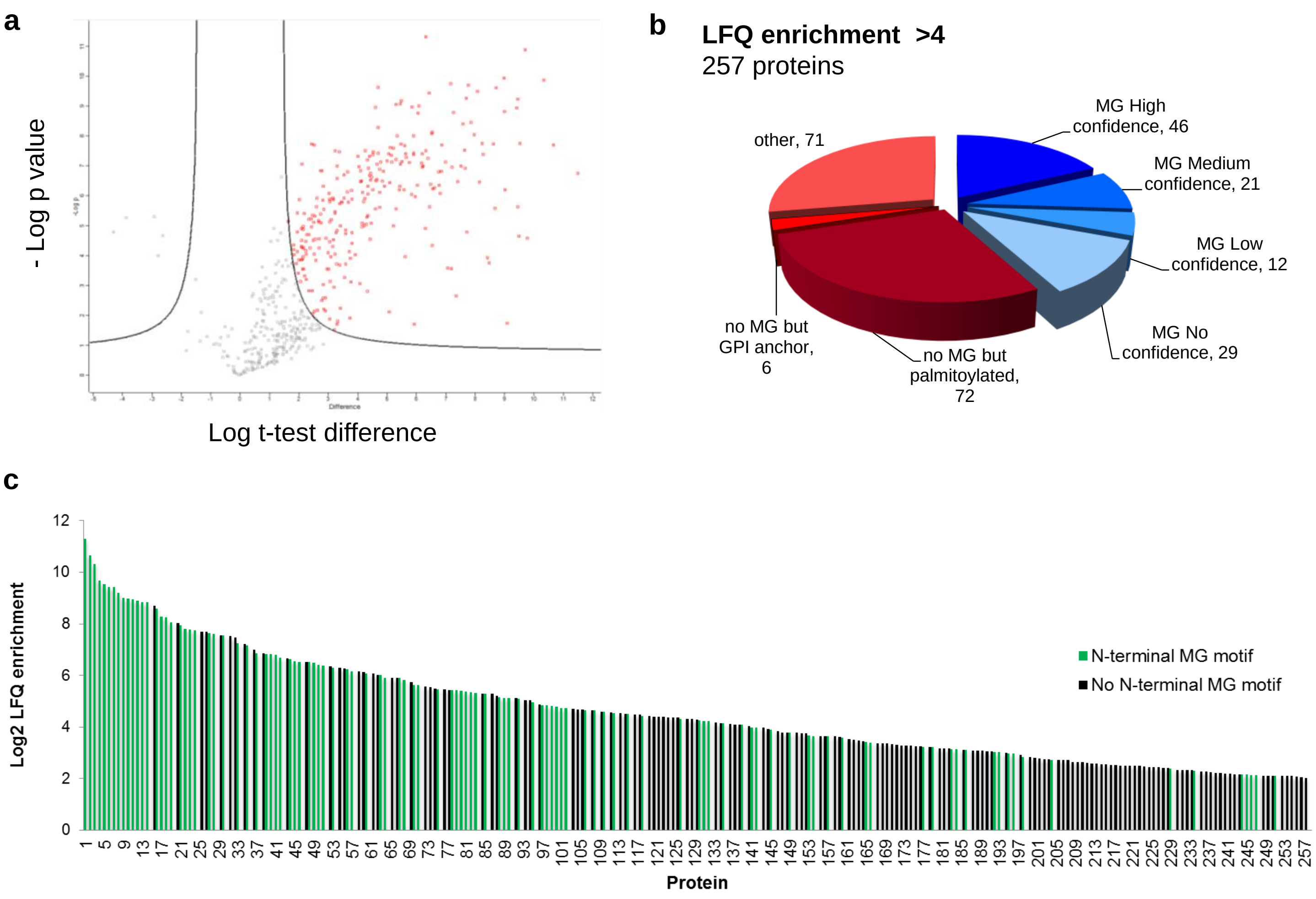

Supplementary Figure 2: Label-free quantification (LFQ) of enrichment of YnMyr-labeled proteins in mammalian cells. a) Two-sample test. To select the significantly enriched proteins in the YnMyr biological replicates (n=4), a two-sample test was carried out in Perseus (modified t-test (permutation-based FDR)) using the following criteria: 250 permutations; FDR 0.001; S0 = 1. The –log p value was plotted against the difference of mean of the Myr biological replicates (n=4) and YnMyr biological replicates (n=4) (log_2 values). The proteins shown in red were significantly enriched in the YnMyr samples. b) 257 substrates were identified by label-free quantification (LFQ) in HeLa cells. 108 proteins had an N-terminal MG motif, as required for *N*-myristoylation. Prediction of *N*-myristoylation was done by two online bioinformatic tools (the MYR predictor and the Myristoylator). Some proteins with no N-terminal MG motif are known to be palmitoylated or incorporate a GPI anchor. c) Log_2 LFQ enrichment was plotted for each protein significantly enriched in the YnMyr samples. Proteins shown in green have an N-terminal MG motif and proteins shown in black do not have an N-terminal MG motif.

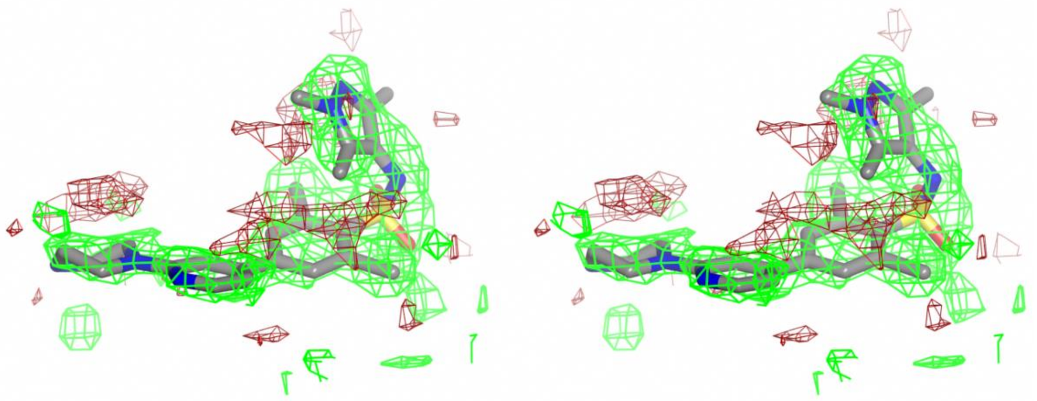

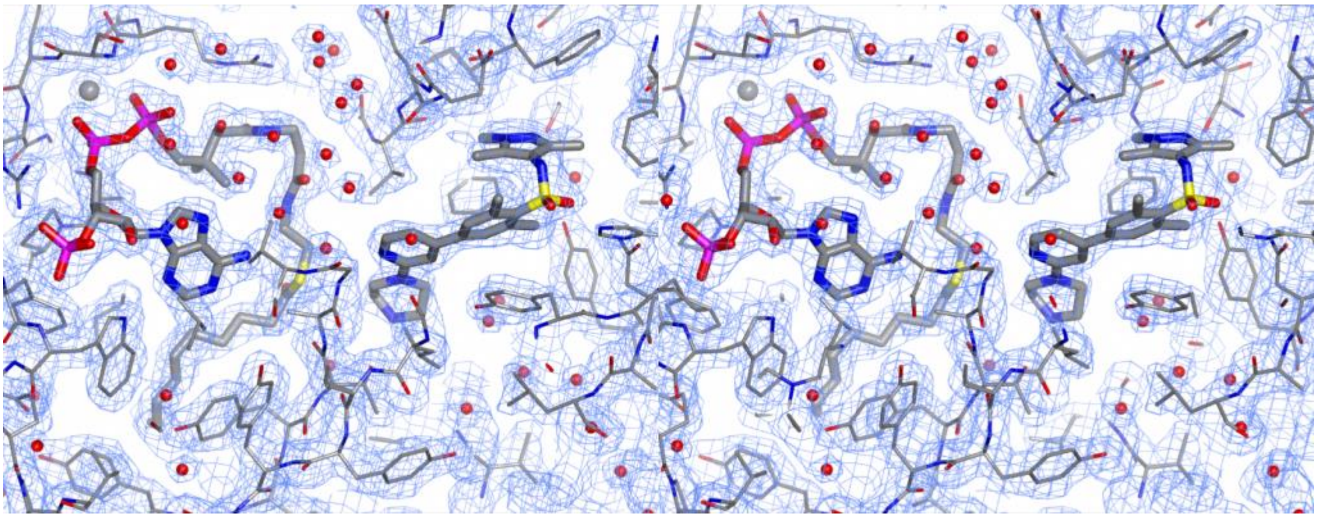

Supplementary Figure 3: Crystal structure of inhibitor 1 bound to HsNMT1. Top panel: Stereo view of initial electron density maps calculated at preliminary stages of refinement (in the absence of ligand) contoured at 2.5σ reveal regions of positive (green) and negative (red) density in the difference map (mF_O-dF_C). The ligand is shown in cylinder representation, coloured by atom; carbon (gray), oxygen (red), nitrogen (blue), sulphur (yellow) and chlorine (silver). **Lower panel:** Stereo view of the final, refined electron density map (2mF_O-dF_C) contoured at a level of 1 σ. The inhibitor and myristoyl-CoA is shown in thick cylinder representation, protein in thin cylinder and colored by atom, as above. The red spheres represent water molecules and the grey sphere represents a Mg atom.

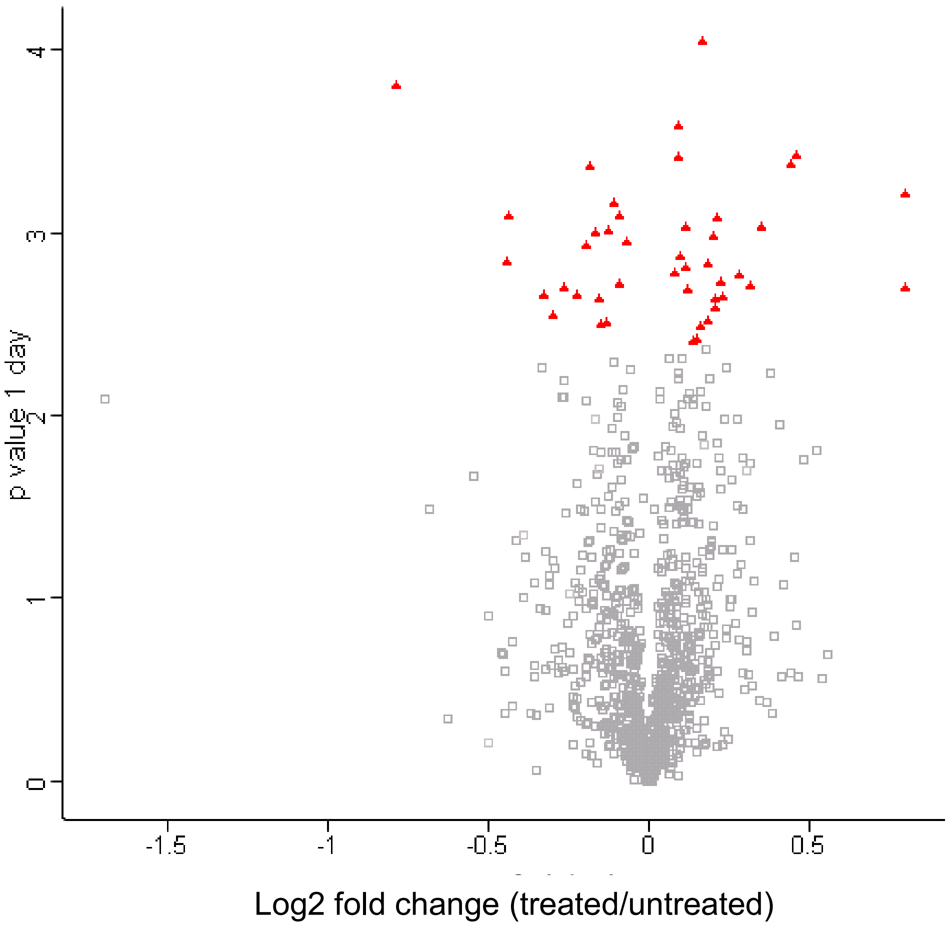

Supplementary Figure 4: quantitative analysis of whole proteome protein abundance following treatment with 1. The HeLa proteome was analyzed by 'spike-in' SILAC (see main text) following 24 hours treatment with 5 µM **1** relative to an untreated control. 1069 proteins were quantified with high confidence and fold change calculated in Perseus. Proteins with a statistically significant change in abundance are highlighted in red; see supplementary methods for experimental details and **Supplementary Data 5** for full dataset.

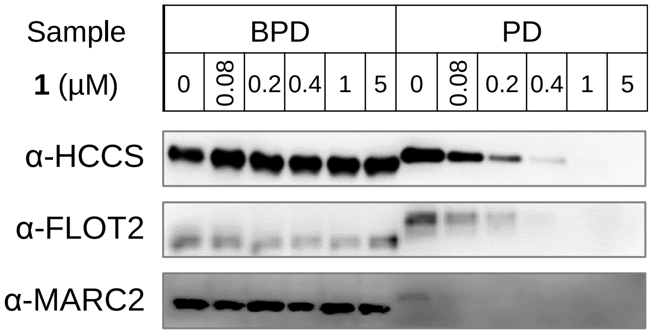

Supplementary Figure 5: Western blot analysis of novel NMT substrates. Proteins were analyzed by Western blot under the same conditions as in Fig. 2d (see main text). Proteins show a band shift to a higher apparent molecular weight on labeling consistent with their modification by CuAAC.

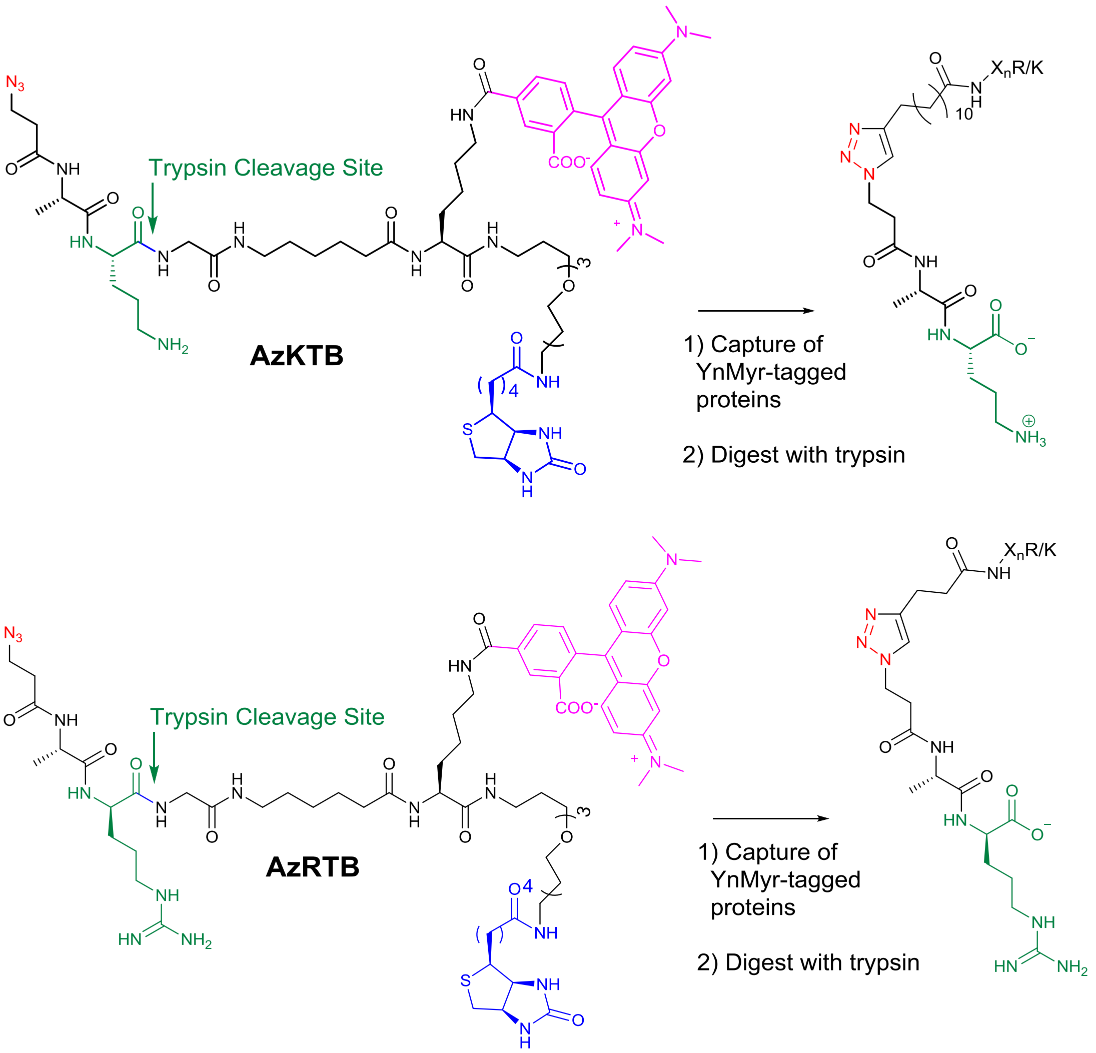

Supplementary Figure 6: **Generation of a tagged solution-phase peptide through on-bead tryptic digest using a) AzKTB or b) AzRTB.** X represents any amino acid except R and K, and n is the number of amino acid in the sequence (n>6 as the the minimum peptide length allowed for the MaxQuant search was seven amino acids)

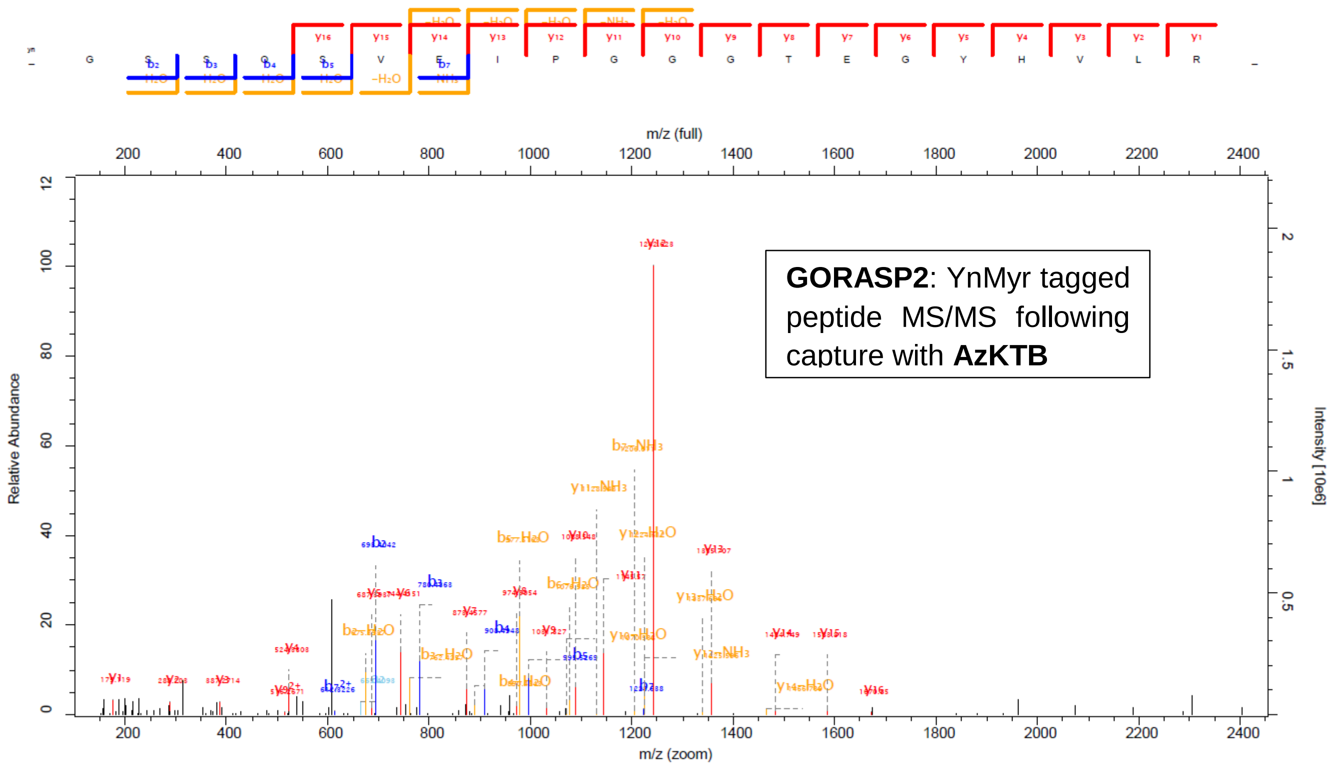

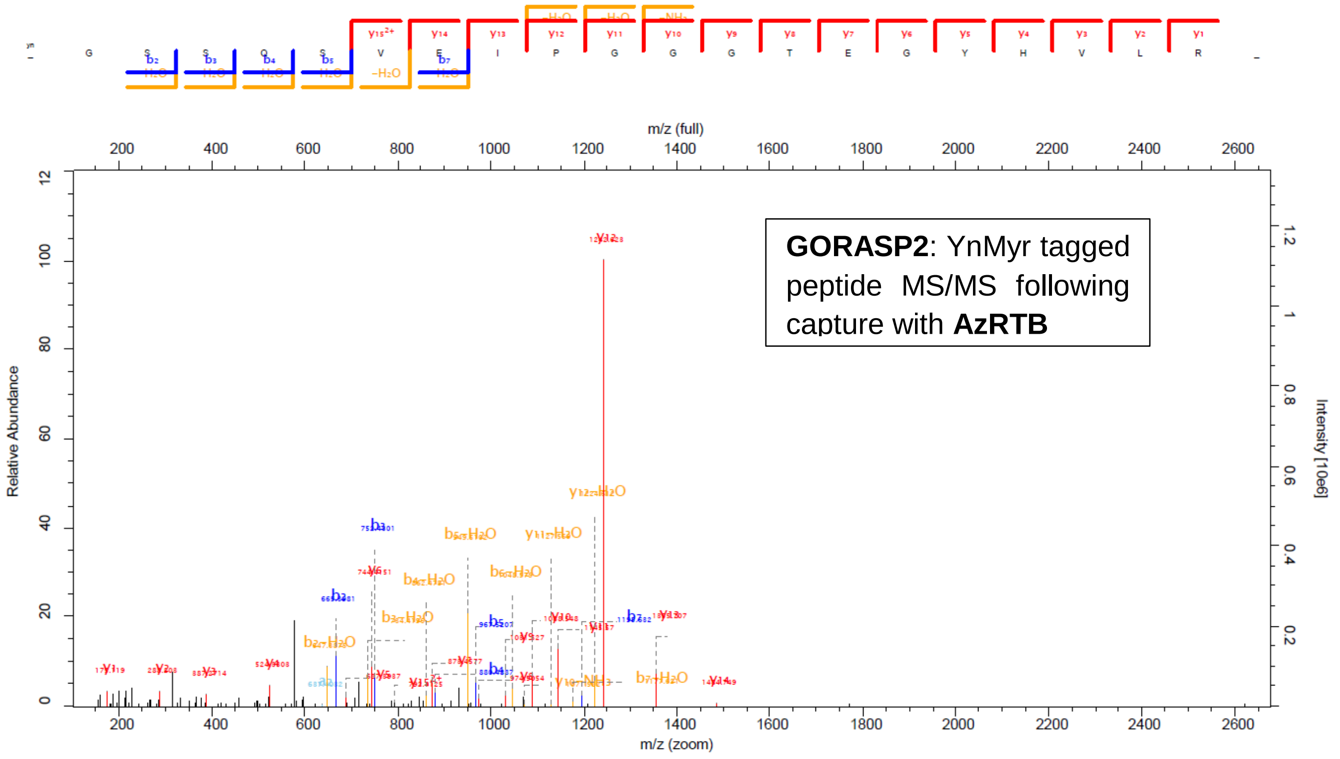

Supplementary Figure 7: Example of an MS/MS spectrum resulting from YnMyr tagging of the N-terminus of a co-translational NMT substrate. MS/MS spectra for the N-terminal **YnMyr**-tagged tryptic peptide derived from Golgi reassembly-stacking protein 2 (GORASP2) captured with **AzKTB** (top) or **AzRTB** (bottom). N-terminal tryptic peptide sequence is GSSQSVEIPGGGTEGYHVLR. Spectra were extracted from standard whole-proteome chemical proteomic analyses using **YnMyr** tagging of HeLa cells, ligation of proteins (CuAAC) to the appropriate capture reagent, affinity enrichment, on-bead digest and nanoLC-MS/MS analysis.

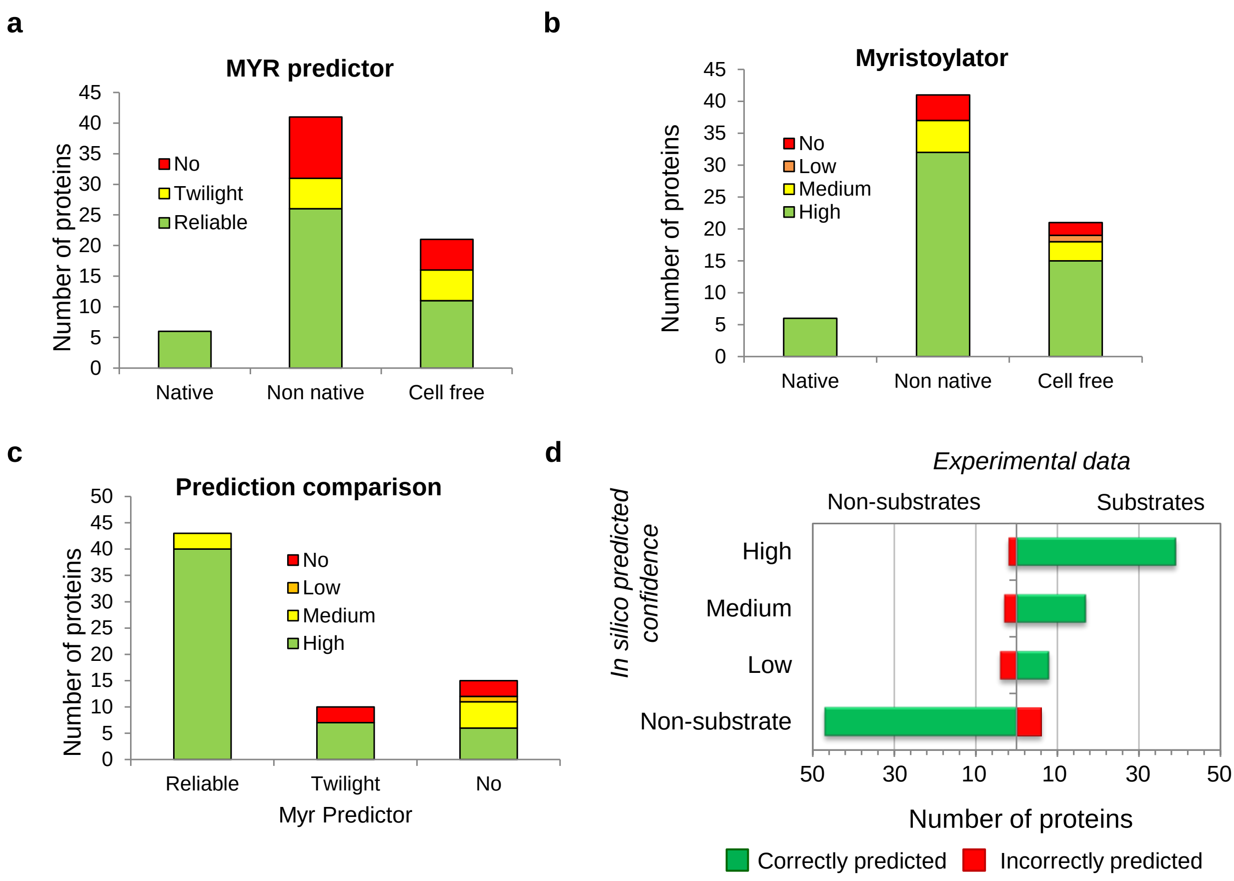

Supplementary Figure 8: Prediction of *N*-myristoylation for known NMT substrates (see Supplementary Table 1). Predictions by a) 'MYR predictor' or b) 'Myristoylator' are shown for proteins identified prior to the present study at native or non-native levels, or in a cell-free system (see Supplementary Table 1). 15 and 6 of these proteins could not be correctly predicted as NMT substrates by the MYR predictor and Myristoylator, respectively. c) The predictions were compared between predictors. 40 proteins predicted as reliable by MYR predictor were also predicted with the highest confidence by Myristoylator. 3 proteins were predicted not to be *N*-myristoylated by both predictors. The predictors disagree on more than 25 predictions. Low and medium predictions made by Myristoylator were considered to be equivalent to twilight prediction by MYR predictor. d) Proportion of bioinformatic predictions (high-medium-low confidence substrates, and non-substrates) validated experimentally in the present study (green) against proportion predicted incorrectly (red).

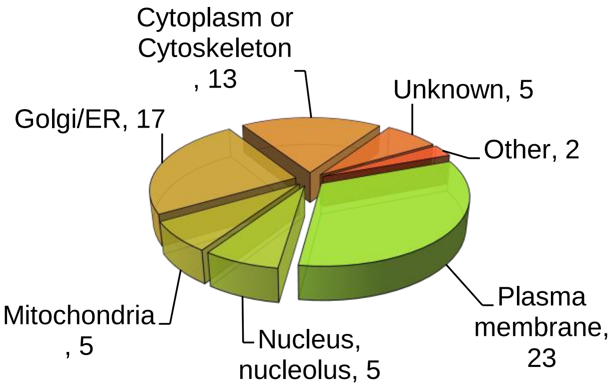

Supplementary Figure 9: Cellular localization for 70 NMT substrates identified in this study (Gene Ontology annotations).

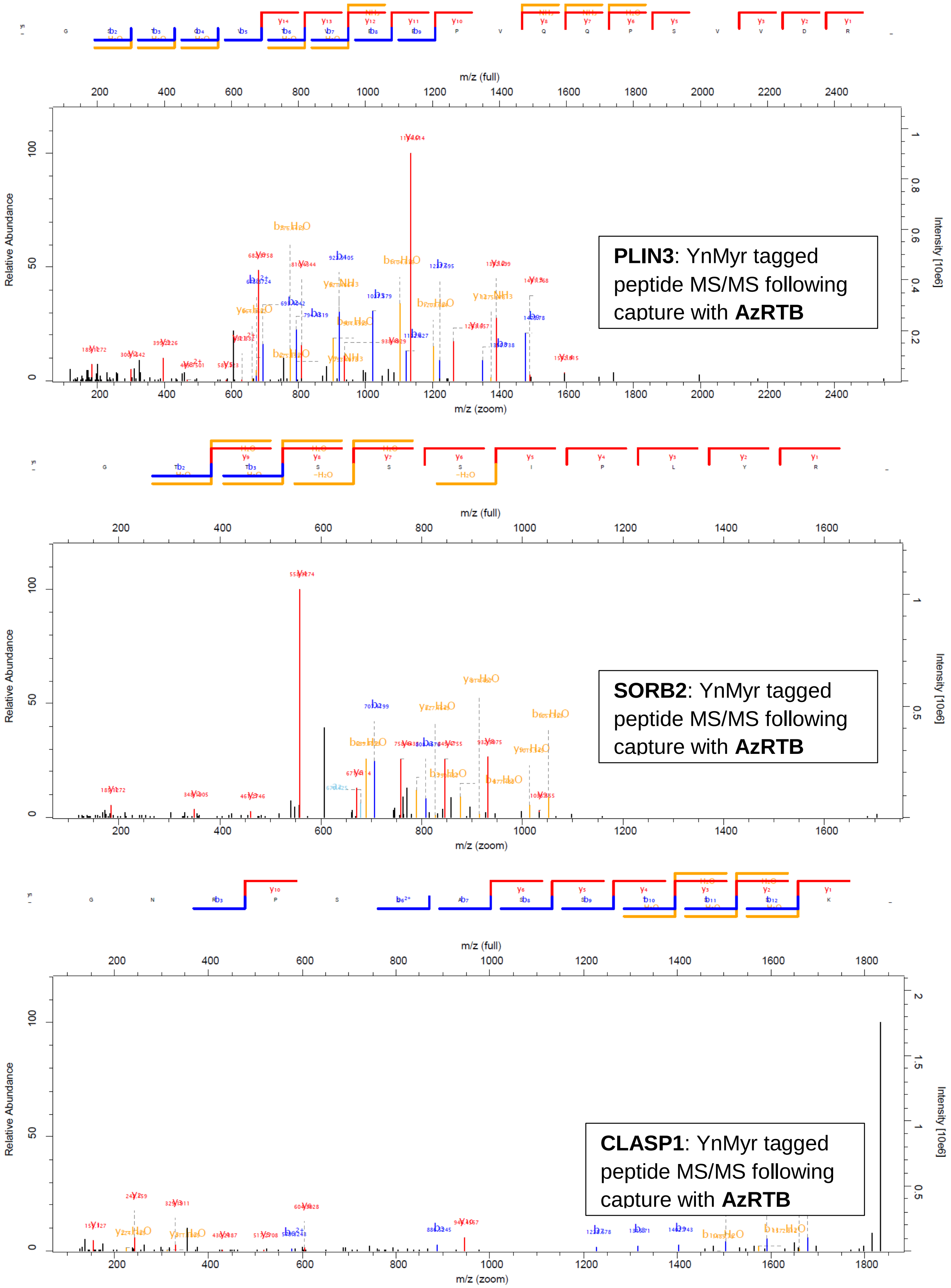

Supplementary Figure 10: Example of MS/MS spectrum from YnMyr tagging of the N-terminus of post-translational NMT substrates. MS/MS spectra for the N-terminal **YnMyr**-tagged tryptic peptide derived from Perilipin-3 (PLIN3), Sorbin and SH3 domain-containing protein 2 (SORB2) and CLIP-associating protein 1 (CLASP1), labeled with **AzRTB** as described in Supplementary Fig. 7.

ADCY9	O60503

```
        10         20         30         40         50         60
MASPPHQQLL HHHSTEVSCD SSGDSNSVRV KINPKQLSSN SHPKHCKYSI SSSCSSSGDS
        70         80         90        100        110        120
GGVPRRVGGG GRLRRQKKLP QLFERASSRW WDPKFDSVNL EEACLERCFP QTQRRFRYAL
       130        140        150        160        170        180
FYIGFACLLW SIYFAVHMRS RLIVMVAPAL CFLLVCVGFF LFTFTKLYAR HYAWTSLALT
       190        200        210        220        230        240
LLVFALTLAA QFQVLTPVSG RGDSSNLTAT ARPTDTCLSQ VGSFSMCIEV LFLLYTVMHL
       250        260        270        280        290        300
PLYLSLCLGV AYSVLFETFG YHFRDEACFP SPGAGALHWE LLSRGLLHGC IHAIGVHLFV
       310        320        330        340        350        360
MSQVRSRSTF LKVGQSIMHG KDLEVEKALK ERMIHSVMPR IIADDLMKQG DEESENSVKR
       370        380        390        400        410        420
HATSSPKNRK KKSSIQKAPI AFRPFKMQQI EEVSILFADI VGFTKMSANK SAHALVGLLN
       430        440        450        460        470        480
DLFGRFDRLC EETKCEKIST LGDCYYCVAG CPEPRADHAY CCIEMGLGMI KAIEQFCQEK
       490        500        510        520        530        540
KEMVNMRVGV HTGTVLCGIL GMRRFKFDVW SNDVNLANLM EQLGVAGKVH ISEATAKYLD
       550        560        570        580        590        600
DRYEMEDGKV IERLGQSVVA DQLKGLKTYL ISGQRAKESR CSCAEALLSG FEVIDGSQVS
       610        620        630        640        650        660
SGPRGQGTAS SGNVSDLAQT VKTFDNLKTC PSCGITFAPK SEAGAEGGAP QNGCQDEHKN
       670        680        690        700        710        720
STKASGGPNP KTQNGLLSPP QEEKLTNSQT SLCEILQEKG RWAGVSLDQS ALLPLRFKNI
       730        740        750        760        770        780
REKTDAHFVD VIKEDSLMKD YFFKPPINQF SLNFLDQELE RSYRTSYQEE VIKNSPVKTF
       790        800        810        820        830        840
ASPTFSSLLD VFLSTTVFLT LSTTCFLKYE AATVPPPPAA LAVFSAALLL EVLSLAVSIR
       850        860        870        880        890        900
MVFFLEDVMA CTKRLLEWIA GWLPRHCIGA ILVSLPALAV YSHVTSEYET NIHFPVFTGS
       910        920        930        940        950        960
AALIAVVHYC NFCQLSSWMR SSLATVVGAG PLLLLYVSLC PDSSVLTSPL DAVQNFSSER
       970        980        990       1000       1010       1020
NPCNSSVPRD LRRPASLIGQ EVVLVFFLLL LLVWFLNREF EVSYRLHYHG DVEADLHRTK
      1030       1040       1050       1060       1070       1080
IQSMRDQADW LLRNIIPYHV AEQLKVSQTY SKNHDSGGVI FASIVNFSEF YEENYEGGKE
      1090       1100       1110       1120       1130       1140
CYRVLNELIG DFDELLSKPD YSSIEKIKTI GATYMAASGL NTAQAQDGSH PQEHLQILFE
      1150       1160       1170       1180       1190       1200
FAKEMMRVVD DFNNNMLWFN FKLRVGFNHG PLTAGVIGTT KLLYDIWGDT VNIASRMDTT
      1210       1220       1230       1240       1250       1260
GVECRIQVSE ESYRVLSKMG YDFDYRGTVN VKGKGQMKTY LYPKCTDHRV IPQHQLSISP
      1270       1280       1290       1300       1310       1320
DIRVQVDGSI GRSPTDEIAN LVPSVQYVDK TSLGSDSSTQ AKDAHLSPKR PWKEPVKAEE
      1330       1340       1350
RGRFGKAIEK DDCDETGIEE ANELTKLNVS KSV
```

AHNAK2	Q8IVF2;Q8IVF2-3

```
        10         20         30         40         50         60
MCDCFHMVLP TWPGTPGSVS GRQLQPGEPG AETEDDHSVT EGPADEGIRP RPQGSSPVYE
        70         80         90        100        110        120
YTTEAADFGL QEDAPGRQGS AGRRRSWWKR DSGDSRTFFR MSRPEAVQEA TEVTLKTEVE
       130        140        150        160        170        180
AGASGYSVTG GGDQGIFVKQ VLKDSSAAKL FNLREGDQLL STTVFFENIK YEDALKILQY
       190        200        210        220        230        240
SEPYKVQFKI RRQLPAPQDE EWASSDAQHG PQGKEKEDTD VADGCRETPT KTLEGDGDQE
       250        260        270        280        290        300
```

```
RLISKPRVGR GRQSQRERLS WPKFQSIKSK RGPGPQRSHS SSEAYEPRDA HDVSPTSTDT
       310        320        330        340        350        360
EAQLTVERQE QKAGPGSQRR RKFLNLRFRT GSGQGPSSTG QPGRGFQSGV GRAGVLEELG
       370        380        390        400        410        420
PWGDSLEETG AATGSRREER AEQDREVMPA QSMPLPTELG DPRLCEGTPQ EGGLRAARLH
       430        440        450        460        470        480
GKTLEGQAQE TAVAQRKPRA QPTPGMSREG EGEGLQSLEI GIARLSLRDT TEGGTQIGPP
       490        500        510        520        530        540
EIRVRVHDLK TPKFAFSTEK EPERERRLST PQRGKRQDAS SKAGTGLKGE EVEGAGWMPG
       550        560        570        580        590        600
REPTTHAEAQ GDEGDGEEGL QRTRITEEQD KGREDTEGQI RMPKFKIPSL GWSPSKHTKT
       610        620        630        640        650        660
GREKATEDTE QGREGEATAT ADRREQRRTE EGLKDKEDSD SMTNTTKIQL IHDEKRLKKE
       670        680        690        700        710        720
QILTEKEVAT KDSKFKMPKF KMPLFGASAP GKSMEASVDV SAPKVEADVS LLSMQGDLKT
       730        740        750        760        770        780
TDLSVQTPSA DLEVQDGQVD VKLPEGPLPE GASLKGHLPK VQRPSLKMPK VDLKGPKLDL
       790        800        810        820        830        840
KGPKAEVTAP DVKMSLSSME VDVQAPRAKL DGARLEGDLS LADKEVTAKD SKFKMPKFKM
       850        860        870        880        890        900
PSFGVSAPGK SMEDSVDVSA PKVEADVSLS SMQGDLKATD LSIQPPSADL EVQAGQVDVK
       910        920        930        940        950        960
LPEGPVPEGA GPKVHLPKVE MPSFKMPKVD LKGPQIDVKG PKLDLKGPKA EVTAPDGEVS
       970        980        990       1000       1010       1020
LPSMEVDVQA QKAKLDGAWL EGDLSLADKD VTAKDSKFKM PKFKMPSFGV SAPGKSIKAL
      1030       1040       1050       1060       1070       1080
VDVSAPKVEA DLSLPSMQGD LKTTDLSIQP ASTDLKVQAD QVDVKLPEGH LPEGAGLKGH
      1090       1100       1110       1120       1130       1140
LPKVEMPSFK MPKVALKGPQ VDVKGPKLDL KSPKAEVTAP DVEVSLPSVE VDVEAPGAKL
      1150       1160       1170       1180       1190       1200
DSARLEGELS LADKDVTAKD SRFKMPKFKM PSFGASAPGK SIEASVDVSA PKVEADVSLP
      1210       1220       1230       1240       1250       1260
SMQGDLKTTD LSIQPPSADL EVHAGQVDVK LLEGHVPEGA GFKGHLPKVQ MPSLKMPKVD
      1270       1280       1290       1300       1310       1320
LKGPQVEVRG PKLDLKGHKA EVTAHEVAVS LPSVEVDMQA PGAKLDGAQL DGDLSLADKD
      1330       1340       1350       1360       1370       1380
VTAKDSKFKM PKFKMPSFGV SAPGKSIEAS VDLSAPKVEA DMSLPSMQGD LKTTDLSIQP
      1390       1400       1410       1420       1430       1440
PSTDLELQAG QLDVKLPEGP VPEGAGLKGH LPKLQMPSFK VPKVDLKGPE IDIKGPKLDL
      1450       1460       1470       1480       1490       1500
KDPKVEVTAP DVEVSLPSVE VDVEAPGAKL DGGRLEEDMS LADKDLTTKD SKFKMPKFKM
      1510       1520       1530       1540       1550       1560
PSFGVSAPGK SIEASVDVSA PKVEADVSLP SMQGDLKATD LSIQPPSADL EVQAGQVDVK
      1570       1580       1590       1600       1610       1620
LPEGPVSEGA GLKGHLPKVQ MPSFKMPKVD LKGPQIDVKG PKLDLKGPKV EVTAPDVKMS
      1630       1640       1650       1660       1670       1680
LSSMEVDVQA PRAKLDGAQL EGDLSLADKA VTAKDSKFKM PKFKMPSFGV SAPGKSIEAS
      1690       1700       1710       1720       1730       1740
VDVSEPKVEA DVSLPSMQGD LKTTDLSIQS PSADLEVQAG QVNVKLPEGP LPEGAGFKGH
      1750       1760       1770       1780       1790       1800
LPKVQMPSLK MPKVALKGPQ MDVKGPKLDL KGPKAEVMAP DVEVSLPSVE VDVEAPGAKL
      1810       1820       1830       1840       1850       1860
DSVRLEGDLS LADKDVTAKD SKFKMPKFKM PSFGVSAPGK SIEASVDVSA PKVEAEVSLP
      1870       1880       1890       1900       1910       1920
SMQGDLKTTD LCIPLPSADL VVQAGQVDMK LPEGQVPEGA GLKGHLPKVD MPSFKMPKVD
      1930       1940       1950       1960       1970       1980
LKGPQTDVKG AKLDLKGPKA EVTAPDVEVS LPSMEVDVQA QKAKLDGARL EGDLSLADKD
      1990       2000       2010       2020       2030       2040
MTAKDSKFKM PKFKMPSFGV SAPGRSIEAS VDVPAPKVEA DVSLPSMQGD LKTTDLSIQP
      2050       2060       2070       2080       2090       2100
PSADLKVQTG QVDVKLPEGH VPEGAGLKGH LPKVEMPSLK MPKVDLKGPQ VDIKGPKLDL
      2110       2120       2130       2140       2150       2160
KDPKVEMRVP DVEVSLPSME VDVQAPRAKL DSAHLQGDLT LANKDLTTKD SKFKMPKFKM
      2170       2180       2190       2200       2210       2220
PSFGVSAPGK SIEASVDVSP PKVEADMSLP SMQGDLKTTD LSIQPLSADV KVQAGQVDVK
```

```
      2230       2240       2250       2260       2270       2280
LLEGPVPEEV GLKGHLPKLQ MPSFKVPKVD LKGPEIDIKG PKLDLKDPKV EVTAPDVEVS
      2290       2300       2310       2320       2330       2340
LPSVEVDVKA PGAKLDGARL EGDMSLADKD VTAKDSKFKM PKFKMLSFGV SALGKSIEAS
      2350       2360       2370       2380       2390       2400
ADVSALKVEA DVSLPSMQGD LKTTDLSVQP PSADLEVQAG QVDVKLPEGP VPEGAGLKGH
      2410       2420       2430       2440       2450       2460
LPKLQMPSFK MPKVDLKGPQ IDVKGPKLDL KGPKTDVMAP DVEVSQPSVE VDVEAPGAKL
      2470       2480       2490       2500       2510       2520
DGAWLEGDLS VADKDVTTKD SRFKIPKFKM PSFGVSAPGK SIEASVDVSA PKVEADGSLS
      2530       2540       2550       2560       2570       2580
SMQGDLKATD LSIQPPSADL EVQAGQVDVK LPEGPVPEGA GLKGHLPKVQ MPSFKMPEMD
      2590       2600       2610       2620       2630       2640
LKGPQLDVKG PKLDLKGPKA EVTAPDVEMS LSSMEVDVQA PRAKLDGARL EGDLSLADKG
      2650       2660       2670       2680       2690       2700
VTAKDSKFKM PKFKMPSFRV SAPGESIEAL VDVSELKVEA DMSLPSMQGD LKTTDISIQP
      2710       2720       2730       2740       2750       2760
PSAQLEVQAG QVDVKLPEGH VPEGAGLKGH LPKLQMPSFK MPEVDLKGPQ IDVKGPNVDL
      2770       2780       2790       2800       2810       2820
KGPKAEVTAP DVKMSLSSME VDVQAPRAKL DGARLEGDLS LADKGMTAKD SKFKMPKFKM
      2830       2840       2850       2860       2870       2880
PSFGVSAPGK SIEASVDVSE LKVEADGSFP SMQGDLKTTD IRIQPPSAQL EVQAGQVDVK
      2890       2900       2910       2920       2930       2940
LPEGHVPEGA GLKGHLPKVQ MPSFKMPKVD LKGPQIDVKG PKLDLKGPKA EVTAPDVEVS
      2950       2960       2970       2980       2990       3000
LPSVEVDVEA PRAKLDGARL EGDLSLADKD VTAKDSKFKM PKFKMPSFGV SAPGKSIEVS
      3010       3020       3030       3040       3050       3060
VDVSAPKVEA EVSLPSMQGD LKTTDISIEP PSAQLEVQAG QVDLKLPEGH VPEGAGLKGH
      3070       3080       3090       3100       3110       3120
LPKLQMPSFK MPKVDRKGPQ IDVKGPKLDL KGPKTDVTAP DVEVSQPGME VDVEAPGAKL
      3130       3140       3150       3160       3170       3180
DGARLEGDLS LADKDVTAKD SKFKMPKFKM PSFGVSAPGK SIEVLVDVSA PKVEADLSLP
      3190       3200       3210       3220       3230       3240
SMQGDLKNTD ISIEPPSAQL EVQAGQVDVK LPEGHVLEGA GLKGHLPKLQ MPSFKMPKVD
      3250       3260       3270       3280       3290       3300
RKGPQIDIKG PKLDLKGPKM DVTAPDVEVS QPSMEVDVEA PGAKLDGARL EGDLSLADKD
      3310       3320       3330       3340       3350       3360
VTAKDSKFKM PKFKMPSYRA SAPGKSIQAS VDVSAPKAEA DVSLPSMQGD LKTTDLSIQL
      3370       3380       3390       3400       3410       3420
PSVDLEVQAG QVDVKLPEGH VPEGAGLKGH LPKVEMPSFK MPKVDLKSPQ VDIKGPKLDL
      3430       3440       3450       3460       3470       3480
KVPKAEVTVP DVEVSLPSVE VDVQAPRAKL DGARLEGDLS LAEKDVTAKD SKFKMPKFKM
      3490       3500       3510       3520       3530       3540
PSFGVSAPGR SIEASLDVSA PKVEADVSLS SMQGDLKATD LSIQPPSADL EVQAVQVDVE
      3550       3560       3570       3580       3590       3600
LLEGPVPEGA GLKGHLPKVE MPSLKTPKVD LKGPQIDVKG PKLDLKGPKA EVRVPDVEVS
      3610       3620       3630       3640       3650       3660
LPSVEVDVQA PKAKLDAGRL EGDLSLADKD VTAKDSKFKM PKFKMPSFRV SAPGKSMEAS
      3670       3680       3690       3700       3710       3720
VDVSAPKVEA DVSLPSMQGD LKTTDLSIQP PSADLKVQAG QMDVKLPEGQ VPEGAGLKEH
      3730       3740       3750       3760       3770       3780
LPKVEMPSLK MPKVDLKGPQ VDIKGPKLDL KVSKAEVTAP DVEVSLPSVE VDVQAPRAKL
      3790       3800       3810       3820       3830       3840
DSAQLEGDLS LADKDVTAKD SKFKMPKFKM PSFGVSAPGK SIEASVHVSA PKVEADVSLP
      3850       3860       3870       3880       3890       3900
SMQGDLKTTD LSIQPHSADL TVQARQVDMK LLEGHVPEEA GLKGHLPKVQ MPSFKMPKVD
      3910       3920       3930       3940       3950       3960
LKGPEIDIKG PKLDLKDPKV EVTAPDVEVS LPSVEVDVEA PGAKLDGARL EGDLSLADKD
      3970       3980       3990       4000       4010       4020
MTAKDSKFKM PKFKMPSFGV SAPGKSMEAS VDVTAPKVEA DVSLPSMQGD LKATDLSVQP
      4030       4040       4050       4060       4070       4080
PSADLEVQAG QVDVKLPEGP VPEGASLKGH LPKVQMPSFK MPKVDLKGPQ IDVKGPKLDL
      4090       4100       4110       4120       4130       4140
KGPKAEVTAP DVKMSLSSME VDVQAPRAKL DGVQLEGDLS LADKDVTAKD SKFKMPKFKM
      4150       4160       4170       4180       4190       4200
```

PSFGVSAPGK SMEASVDVSE LKAKADVSLP SMQGDLKTTD LSIQSPSADL EVQAGQVDVK
4210 4220 4230 4240 4250 4260
LPEGPLPKGA GLKGHLPKVQ MPCLKMPKVA LKGPQVDVKG PKLDLKGPKA DVMTPVVEVS
4270 4280 4290 4300 4310 4320
LPSMEVDVEA PGAKLDSVRL EGDLSLADKD MTAKDSKFKM PKFKMPSFGV SAPGKSIEAS
4330 4340 4350 4360 4370 4380
LDVSALKVEA DVSLPSMQGD LKTTHLSIQP PSADLEVQAG QEDVKLPEGP VHEGAGLKGH
4390 4400 4410 4420 4430 4440
LPKLQMPSFK VPKVDLKGPQ IDVNVPKLDL KGPKVEVTSP NLDVSLPSME VDIQAPGAKL
4450 4460 4470 4480 4490 4500
DSTRLEGDLS LADKDVTAKD SKFKMPKFKM PSFGMLSPGK SIEVSVDVSA PKMEADMSIP
4510 4520 4530 4540 4550 4560
SMQGDLKTTD LRIQAPSADL EVQAGQVDLK LPEGHMPEVA GLKGHLPKVE MPSFKMPKVD
4570 4580 4590 4600 4610 4620
LKGPQVDVKG PKLDLKGPKA EVMAPDVEVS LPSVETDVQA PGSMLDGARL EGDLSLAHED
4630 4640 4650 4660 4670 4680
VAGKDSKFQG PKLSTSGFEW SSKKVSMSSS EIEGNVTFHE KTSTFPIVES VVHEGDLHDP
4690 4700 4710 4720 4730 4740
SRDGNLGLAV GEVGMDSKFK KLHFKVPKVS FSSTKTPKDS LVPGAKSSIG LSTIPLSSSE
4750 4760 4770 4780 4790 4800
CSSFELQQVS ACSEPSMQMP KVGFAGFPSS RLDLTGPHFE SSILSPCEDV TLTKYQVTVP
4810 4820 4830 4840 4850 4860
RAALAPELAL EIPSGSQADI PLPKTECSTD LQPPEGVPTS QAESHSGPLN SMIPVSLGQV
4870 4880 4890 4900 4910 4920
SFPKFYKPKF VFSVPQMAVP EGDLHAAVGA PVMSPLSPGE RVQCPLPSTQ LPSPGTCVSQ
4930 4940 4950 4960 4970 4980
GPEELVASLQ TSVVAPGEAP SEDADHEGKG SPLKMPKIKL PSFRWSPKKE TGPKVDPECS
4990 5000 5010 5020 5030 5040
VEDSKLSLVL DKDEVAPQSA IHMDLPPERD GEKGRSTKPG FAMPKLALPK MKASKSGVSL
5050 5060 5070 5080 5090 5100
PQRDVDPSLS SATAGGSFQD TEKASSDGGR GGLGATASAT GSEGVNLHRP QVHIPSLGFA
5110 5120 5130 5140 5150 5160
KPDLRSSKAK VEVSQPEADL PLPKHDLSTE GDSRGCGLGD VPVSQPCGEG IAPTPEDPLQ
5170 5180 5190 5200 5210 5220
PSCRKPDAEV LTVESPEEEA MTKYSQESWF KMPKFRMPSL RRSFRDRGGA GKLEVAQTQA
5230 5240 5250 5260 5270 5280
PAATGGEAAA KVKEFLVSGS NVEAAMSLQL PEADAEVTAS ESKSSTDILR CDLDSTGLKL
5290 5300 5310 5320 5330 5340
HLSTAGMTGD ELSTSEVRIH PSKGPLPFQM PGMRLPETQV LPGEIDETPL SKPGHDLASM
5350 5360 5370 5380 5390 5400
EDKTEKWSSQ PEGPLKLKAS STDMPSQISV VNVDQLWEDS VLTVKFPKLM VPRFSFPAPS
5410 5420 5430 5440 5450 5460
SEDDVFIPTV REVQCPEANI DTALCKESPG LWGASILKAG AGVPGEQPVD LNLPLEAPPI
5470 5480 5490 5500 5510 5520
SKVRVHIQGA QVESQEVTIH SIVTPEFVDL SVPRTFSTQI VRESEIPTSE IQTPSYGFSL
5530 5540 5550 5560 5570 5580
LKVKIPEPHT QARVYTTMTQ HSRTQEGTEE APIQATPGVD SISGDLQPDT GEPFEMISSS
5590 5600 5610 5620 5630 5640
VNVLGQQTLT FEVPSGHQLA DSCSDEEPAE ILEFPPDDSQ EATTPLADEG RAPKDKPESK
5650 5660 5670 5680 5690 5700
KSGLLWFWLP NIGFSSSVDE TGVDSKNDVQ RSAPIQTQPE ARPEAELPKK QEKAGWFRFP
5710 5720 5730 5740 5750 5760
KLGFSSSPTK KSKSTEDGAE LEEQKLQEET ITFFDARESF SPEEKEEGEL IGPVGTGLDS
5770 5780 5790
RVMVTSAART ELILPEQDRK ADDESKGSGL GPNEG

C2orf49	Q9BVC5

10 20 30 40 50 60
MAGDVGGRSC TDSELLLHPE LLSQEFLLLT LEQKNIAVET DVRVNKDSLT DLYVQHAIPL
70 80 90 100 110 120
PQRDLPKNRW GKMMEKKREQ HEIKNETKRS STVDGLRKRP LIVFDGSSTS TSIKVKKTEN
130 140 150 160 170 180
GDNDRLKPPP QASFTSNAFR KLSNSSSSVS PLILSSNLPV NNKTEHNNND AKQNHDLTHR
190 200 210 220 230
KSPSGPVKSP PLSPVGTTPV KLKRAAPKEE AEAMNNLKPP QAKRKIQHVT WP

CLASP1	Q7Z460;Q7Z460-3;Q7Z460-2

```
        10         20         30         40         50         60
MEPRMESCLA QVLQKDVGKR LQVGQELIDY FSDKQKSADL EHDQTMLDKL VDGLATSWVN
        70         80         90        100        110        120
SSNYKVVLLG MDILSALVTR LQDRFKAQIG TVLPSLIDRL GDAKDSVREQ DQTLLLKIMD
       130        140        150        160        170        180
QAANPQYVWD RMLGGFKHKN FRTREGICLC LIATLNASGA QTLTLSKIVP HICNLLGDPN
       190        200        210        220        230        240
SQVRDAAINS LVEIYRHVGE RVRADLSKKG LPQSRLNVIF TKFDEVQKSG NMIQSANDKN
       250        260        270        280        290        300
FDDEDSVDGN RPSSASSTSS KAPPSSRRNV GMGTTRRLGS STLGSKSSAA KEGAGAVDEE
       310        320        330        340        350        360
DFIKAFDDVP VVQIYSSRDL EESINKIREI LSDDKHDWEQ RVNALKKIRS LLLAGAAEYD
       370        380        390        400        410        420
NFFQHLRLLD GAFKLSAKDL RSQVVREACI TLGHLSSVLG NKFDHGAEAI MPTIFNLIPN
       430        440        450        460        470        480
SAKIMATSGV VAVRLIIRHT HIPRLIPVIT SNCTSKSVAV RRRCFEFLDL LLQEWQTHSL
       490        500        510        520        530        540
ERHISVLAET IKKGIHDADS EARIEARKCY WGFHSHFSRE AEHLYHTLES SYQKALQSHL
       550        560        570        580        590        600
KNSDSIVSLP QSDRSSSSSQ ESLNRPLSAK RSPTGSTTSR ASTVSTKSVS TTGSLQRSRS
       610        620        630        640        650        660
DIDVNAAASA KSKVSSSSGT TPFSSAAALP PGSYASLGRI RTRRQSSGSA TNVASTPDNR
       670        680        690        700        710        720
GRSRAKVVSQ SQRSRSANPA GAGSRSSSPG KLLGSGYGGL TGGSSRGPPV TPSSEKRSKI
       730        740        750        760        770        780
PRSQGCSRET SPNRIGLARS SRIPRPSMSQ GCSRDTSRES SRDTSPARGF PPLDRFGLGQ
       790        800        810        820        830        840
PGRIPGSVNA MRVLSTSTDL EAAVADALKK PVRRRYEPYG MYSDDDANSD ASSVCSERSY
       850        860        870        880        890        900
GSRNGGIPHY LRQTEDVAEV LNHCASSNWS ERKEGLLGLQ NLLKSQRTLS RVELKRLCEI
       910        920        930        940        950        960
FTRMFADPHS KRVFSMFLET LVDFIIIHKD DLQDWLFVLL TQLLKKMGAD LLGSVQAKVQ
       970        980        990       1000       1010       1020
KALDVTRDSF PFDQQFNILM RFIVDQTQTP NLKVKVAILK YIESLARQMD PTDFVNSSET
      1030       1040       1050       1060       1070       1080
RLAVSRIITW TTEPKSSDVR KAAQIVLISL FELNTPEFTM LLGALPKTFQ DGATKLLHNH
      1090       1100       1110       1120       1130       1140
LKNSSNTSVG SPSNTIGRTP SRHTSSRTSP LTSPTNCSHG GLSPSRLWGW SADGLAKHPP
      1150       1160       1170       1180       1190       1200
PFSQPNSIPT APSHKALRRS YSPSMLDYDT ENLNSEEIYS SLRGVTEAIE KFSFRSQEDL
      1210       1220       1230       1240       1250       1260
NEPIKRDGKK ECDIVSRDGG AASPATEGRG GSEVEGGRTA LDNKTSLLNT QPPRAFPGPR
      1270       1280       1290       1300       1310       1320
ARDYNPYPYS DAINTYDKTA LKEAVFDDDM EQLRDVPIDH SDLVADLLKE LSNHNERVEE
      1330       1340       1350       1360       1370       1380
RKGALLELLK ITREDSLGVW EEHFKTILLL LLETLGDKDH SIRALALRVL REILRNQPAR
      1390       1400       1410       1420       1430       1440
FKNYAELTIM KTLEAHKDSH KEVVRAAEEA ASTLASSIHP EQCIKVLCPI IQTADYPINL
      1450       1460       1470       1480       1490       1500
AAIKMQTKVV ERIAKESLLQ LLVDIIPGLL QGYDNTESSV RKASVFCLVA IYSVIGEDLK
      1510       1520       1530
PHLAQLTGSK MKLLNLYIKR AQTTNSNSSS SSDVSTHS
```

CLASP2	O75122;O75122-2

```
        10         20         30         40         50         60
MAMGDDKSFD DEESVDGNRP SSAASAFKVP APKTSGNPAN SARKPGSAGG PKVGGASKEG
        70         80         90        100        110        120
GAGAVDEDDF IKAFTDVPSI QIYSSRELEE TLNKIREILS DDKHDWDQRA NALKKIRSLL
       130        140        150        160        170        180
VAGAAQYDCF FQHLRLLDGA LKLSAKDLRS QVVREACITV AHLSTVLGNK FDHGAEAIVP
       190        200        210        220        230        240
```

TLFNLVPNSA KVMATSGCAA IRFIIRHTHV PRLIPLITSN CTSKSVPVRR RSFEFLDLLL
250 260 270 280 290 300
QEWQTHSLER HAAVLVETIK KGIHDADAEA RVEARKTYMG LRNHFPGEAE TLYNSLEPSY
310 320 330 340 350 360
QKSLQTYLKS SGSVASLPQS DRSSSSSQES LNRPFSSKWS TANPSTVAGR VSAGSSKASS
370 380 390 400 410 420
LPGSLQRSRS DIDVNAAAGA KAHHAAGQSV RRGRLGAGAL NAGSYASLED TSDKLDGTAS
430 440 450 460 470 480
EDGRVRAKLS APLAGMGNAK ADSRGRSRTK MVSQSQPGSR SGSPGRVLTT TALSTVSSGV
490 500 510 520 530 540
QRVLVNSASA QKRSKIPRSQ GCSREASPSR LSVARSSRIP RPSVSQGCSR EASRESSRDT
550 560 570 580 590 600
SPVRSFQPLA SRHHSRSTGA LYAPEVYGAS GPGYGISQSS RLSSSVSAMR VLNTGSDVEE
610 620 630 640 650 660
AVADALKKPA RRRYESYGMH SDDDANSDAS SACSERSYSS RNGSIPTYMR QTEDVAEVLN
670 680 690 700 710 720
RCASSNWSER KEGLLGLQNL LKNQRTLSRV ELKRLCEIFT RMFADPHGKR VFSMFLETLV
730 740 750 760 770 780
DFIQVHKDDL QDWLFVLLTQ LLKKMGADLL GSVQAKVQKA LDVTRESFPN DLQFNILMRF
790 800 810 820 830 840
TVDQTQTPSL KVKVAILKYI ETLAKQMDPG DFINSSETRL AVSRVITWTT EPKSSDVRKA
850 860 870 880 890 900
AQSVLISLFE LNTPEFTMLL GALPKTFQDG ATKLLHNHLR NTGNGTQSSM GSPLTRPTPR
910 920 930 940 950 960
SPANWSSPLT SPTNTSQNTL SPSAFDYDTE NMNSEDIYSS LRGVTEAIQN FSFRSQEDMN
970 980 990 1000 1010 1020
EPLKRDSKKD DGDSMCGGPG MSDPRAGGDA TDSSQTALDN KASLLHSMPT HSSPRSRDYN
1030 1040 1050 1060 1070 1080
PYNYSDSISP FNKSALKEAM FDDDADQFPD DLSLDHSDLV AELLKELSNH NERVEERKIA
1090 1100 1110 1120 1130 1140
LYELMKLTQE ESFSVWDEHF KTILLLLLET LGDKEPTIRA LALKVLREIL RHQPARFKNY
1150 1160 1170 1180 1190 1200
AELTVMKTLE AHKDPHKEVV RSAEEAASVL ATSISPEQCI KVLCPIIQTA DYPINLAAIK
1210 1220 1230 1240 1250 1260
MQTKVIERVS KETLNLLLPE IMPGLIQGYD NSESSVRKAC VFCLVAVHAV IGDELKPHLS
1270 1280 1290
QLTGSKMKLL NLYIKRAQTG SGGADPTTDV SGQS

CUL4B	Q13620;Q13620-1;Q13620-3;Q13619;Q13619-2

10 20 30 40 50 60
MMSQSSGSGD GNDDEATTSK DGGFSSPSPS AAAAAQEVRS ATDGNTSTTP PTSAKKRKLN
70 80 90 100 110 120
SSSSSSSNSS NEREDFDSTS SSSSTPPLQP RDSASPSTSS FCLGVSVAAS SHVPIQKKLR
130 140 150 160 170 180
FEDTLEFVGF DAKMAEESSS SSSSSSPTAA TSQQQQLKNK SILISSVASV HHANGLAKSS
190 200 210 220 230 240
TTVSSFANSK PGSAKKLVIK NFKDKPKLPE NYTDETWQKL KEAVEAIQNS TSIKYNLEEL
250 260 270 280 290 300
YQAVENLCSY KISANLYKQL RQICEDHIKA QIHQFREDSL DSVLFLKKID RCWQNHCRQM
310 320 330 340 350 360
IMIRSIFLFL DRTYVLQNSM LPSIWDMGLE LFRAHIISDQ KVQNKTIDGI LLLIERERNG
370 380 390 400 410 420
EAIDRSLLRS LLSMLSDLQI YQDSFEQRFL EETNRLYAAE GQKLMQEREV PEYLHHVNKR
430 440 450 460 470 480
LEEEADRLIT YLDQTTQKSL IATVEKQLLG EHLTAILQKG LNNLLDENRI QDLSLLYQLF
490 500 510 520 530 540
SRVRGGVQVL LQQWIEYIKA FGSTIVINPE KDKTMVQELL DFKDKVDHII DICFLKNEKF
550 560 570 580 590 600
INAMKEAFET FINKRPNKPA ELIAKYVDSK LRAGNKEATD EELEKMLDKI MIIFRFIYGK
610 620 630 640 650 660
DVFEAFYKKD LAKRLLVGKS ASVDAEKSML SKLKHECGAA FTSKLEGMFK DMELSKDIMI
670 680 690 700 710 720
QFKQYMQNQN VPGNIELTVN ILTMGYWPTY VPMEVHLPPE MVKLQEIFKT FYLGKHSGRK

```
       730        740        750        760        770        780
LQWQSTLGHC VLKAEFKEGK KELQVSLFQT LVLLMFNEGE EFSLEEIKQA TGIEDGELRR
       790        800        810        820        830        840
TLQSLACGKA RVLAKNPKGK DIEDGDKFIC NDDFKHKLFR IKINQIQMKE TVEEQASTTE
       850        860        870        880        890        900
RVFQDRQYQI DAAIVRIMKM RKTLSHNLLV SEVYNQLKFP VKPADLKKRI ESLIDRDYME
       910
RDKENPNQYN YIA
```

GSN	P06396;P06396-3;P06396-2;CON__Q3SX14

```
        10         20         30         40         50         60
MAPHRPAPAL LCALSLALCA LSLPVRAATA SRGASQAGAP QGRVPEARPN SMVVEHPEFL
        70         80         90        100        110        120
KAGKEPGLQI WRVEKFDLVP VPTNLYGDFF TGDAYVILKT VQLRNGNLQY DLHYWLGNEC
       130        140        150        160        170        180
SQDESGAAAI FTVQLDDYLN GRAVQHREVQ GFESATFLGY FKSGLKYKKG GVASGFKHVV
       190        200        210        220        230        240
PNEVVVQRLF QVKGRRVVRA TEVPVSWESF NNGDCFILDL GNNIHQWCGS NSNRYERLKA
       250        260        270        280        290        300
TQVSKGIRDN ERSGRARVHV SEEGTEPEAM LQVLGPKPAL PAGTEDTAKE DAANRKLAKL
       310        320        330        340        350        360
YKVSNGAGTM SVSLVADENP FAQGALKSED CFILDHGKDG KIFVWKGKQA NTEERKAALK
       370        380        390        400        410        420
TASDFITKMD YPKQTQVSVL PEGGETPLFK QFFKNWRDPD QTDGLGLSYL SSHIANVERV
       430        440        450        460        470        480
PFDAATLHTS TAMAAQHGMD DDGTGQKQIW RIEGSNKVPV DPATYGQFYG GDSYIILYNY
       490        500        510        520        530        540
RHGGRQGQII YNWQGAQSTQ DEVAASAILT AQLDEELGGT PVQSRVVQGK EPAHLMSLFG
       550        560        570        580        590        600
GKPMIIYKGG TSREGGQTAP ASTRLFQVRA NSAGATRAVE VLPKAGALNS NDAFVLKTPS
       610        620        630        640        650        660
AAYLWVGTGA SEAEKTGAQE LLRVLRAQPV QVAEGSEPDG FWEALGGKAA YRTSPRLKDK
       670        680        690        700        710        720
KMDAHPPRLF ACSNKIGRFV IEEVPGELMQ EDLATDDVML LDTWDQVFVW VGKDSQEEEK
       730        740        750        760        770        780
TEALTSAKRY IETDPANRDR RTPITVVKQG FEPPSFVGWF LGWDDDYWSV DPLDRAMAEL
AA
```

ILF3	Q12906;Q12906-3;Q12906-2;Q12906-5;Q12906-7;Q12906-6;Q12906-4;Q96SI9;Q96SI9-2

```
        10         20         30         40         50         60
MRPMRIFVND DRHVMAKHSS VYPTQEELEA VQNMVSHTER ALKAVSDWID EQEKGSSEQA
        70         80         90        100        110        120
ESDNMDVPPE DDSKEGAGEQ KTEHMTRTLR GVMRVGLVAK GLLLKGDLDL ELVLLCKEKP
       130        140        150        160        170        180
TTALLDKVAD NLAIQLAAVT EDKYEILQSV DDAAIVIKNT KEPPLSLTIH LTSPVVREEM
       190        200        210        220        230        240
EKVLAGETLS VNDPPDVLDR QKCLAALASL RHAKWFQARA NGLKSCVIVI RVLRDLCTRV
       250        260        270        280        290        300
PTWGPLRGWP LELLCEKSIG TANRPMGAGE ALRRVLECLA SGIVMPDGSG IYDPCEKEAT
       310        320        330        340        350        360
DAIGHLDRQQ REDITQSAQH ALRLAAFGQL HKVLGMDPLP SKMPKKPKNE NPVDYTVQIP
       370        380        390        400        410        420
PSTTYAITPM KRPMEEDGEE KSPSKKKKKI QKKEEKAEPP QAMNALMRLN QLKPGLQYKL
       430        440        450        460        470        480
VSQTGPVHAP IFTMSVEVDG NSFEASGPSK KTAKLHVAVK VLQDMGLPTG AEGRDSSKGE
       490        500        510        520        530        540
DSAEETEAKP AVVAPAPVVE AVSTPSAAFP SDATAEQGPI LTKHGKNPVM ELNEKRRGLK
       550        560        570        580        590        600
YELISETGGS HDKRFVMEVE VDGQKFQGAG SNKKVAKAYA ALAALEKLFP DTPLALDANK
       610        620        630        640        650        660
KKRAPVPVRG GPKFAAKPHN PGFGMGGPMH NEVPPPPNLR GRGRGGSIRG RGRGRGFGGA
```

```
       670        680        690        700        710        720
NHGGYMNAGA GYGSYGYGGN SATAGYSQFY SNGGHSGNAS GGGGGGGGGS SGYGSYYQGD
       730        740        750        760        770        780
NYNSPVPPKH AGKKQPHGGQ QKPSYGSGYQ SHQGQQQSYN QSPYSNYGPP QGKQKGYNHG
       790        800        810        820        830        840
QGSYSYSNSY NSPGGGGGSD YNYESKFNYS GSGGRSGGNS YGSGGASYNP GSHGGYGGGS
       850        860        870        880        890
GGGSSYQGKQ GGYSQSNYNS PGSGQNYSGP PSSYQSSQGG YGRNADHSMN YQYR
```

LRRFIP1	Q32MZ4;Q32MZ4-2;Q32MZ4-3

```
        10         20         30         40         50         60
MTSPAAAQSR EIDCLSPEAQ KLAEARLAAK RAARAEAREI RMKELERQQK EEDSERYSRR
        70         80         90        100        110        120
SRRNTSASDE DERMSVGSRG SLRVEERPEK DFTEKGSRNM PGLSAATLAS LGGTSSRRGS
       130        140        150        160        170        180
GDTSISIDTE ASIREIKELN ELKDQIQDVE GKYMQGLKEM KDSLAEVEEK YKKAMVSNAQ
       190        200        210        220        230        240
LDNEKTNFMY QVDTLKDMLL ELEEQLAESR RQYEEKNKEF EREKHAHSIL QFQFAEVKEA
       250        260        270        280        290        300
LKQREEMLEK HGIILNSEIA TNGETSDTLN NVGYQGPTKM TKEELNALKS TGDGTLGRAS
       310        320        330        340        350        360
EVEVKNEIVA NVGKREILHN TEKEQHTEDT VKDCVDIEVF PAGENTEDQK SSEDTAPFLG
       370        380        390        400        410        420
TLAGATYEEQ VQSQILESSS LPENTVQVES NEVMGAPDDR TRTPLEPSNC WSDLDGGNHT
       430        440        450        460        470        480
ENVGEAAVTQ VEEQAGTVAS CPLGHSDDTV YHDDKCMVEV PQELETSTGH SLEKEFTNQE
       490        500        510        520        530        540
AAEPKEVPAH STEVGRDHNE EEGEETGLRD EKPIKTEVPG SPAGTEGNCQ EATGPSTVDT
       550        560        570        580        590        600
QNEPLDMKEP DEEKSDQQGE ALDSSQKKTK NKKKKNKKKK SPVPVETLKD VKKELTYQNT
       610        620        630        640        650        660
DLSEIKEEEQ VKSTDRKSAV EAQNEVTENP KQKIAAESSE NVDCPENPKI KLDGKLDQEG
       670        680        690        700        710        720
DDVQTAAEEV LADGDTLDFE DDTVQSSGPR AGGEELDEGV AKDNAKIDGA TQSSPAEPKS
       730        740        750        760        770        780
EDADRCTLPE HESPSQDISD ACEAESTERC EMSEHPSQTV RKALDSNSLE NDDLSAPGRE
       790        800
PGHFNPESRE DTRGGNEKGK SKEDCTMS
```

PLIN3	O60664;O60664-4;O60664-3;O60664-2

```
        10         20         30         40         50         60
MSADGAEADG STQVTVEEPV QQPSVVDRVA SMPLISSTCD MVSAAYASTK ESYPHIKTVC
        70         80         90        100        110        120
DAAEKGVRTL TAAAVSGAQP ILSKLEPQIA SASEYAHRGL DKLEENLPIL QQPTEKVLAD
       130        140        150        160        170        180
TKELVSSKVS GAQEMVSSAK DTVATQLSEA VDATRGAVQS GVDKTKSVVT GGVQSVMGSR
       190        200        210        220        230        240
LGQMVLSGVD TVLGKSEEWA DNHLPLTDAE LARIATSLDG FDVASVQQQR QEQSYFVRLG
       250        260        270        280        290        300
SLSERLRQHA YEHSLGKLRA TKQRAQEALL QLSQVLSLME TVKQGVDQKL VEGQEKLHQM
       310        320        330        340        350        360
WLSWNQKQLQ GPEKEPPKPE QVESRALTMF RDIAQQLQAT CTSLGSSIQG LPTNVKDQVQ
       370        380        390        400        410        420
QARRQVEDLQ ATFSSIHSFQ DLSSSILAQS RERVASAREA LDHMVEYVAQ NTPVTWLVGP
       430
FAPGITEKAP EEKK
```

RBM15	Q96T37;Q96T37-3;Q96T37-2;Q8NDT2

```
        10         20         30         40         50         60
MRTAGRDPVP RRSPRWRRAV PLCETSAGRR VTQLRGDDLR RPATMKGKER SPVKAKRSRG
```

70 80 90 100 110 120

GEDSTSRGER SKKLGGSGGS NGSSSGKTDS GGGSRRSLHL DKSSSRGGSR EYDTGGGSSS

130 140 150 160 170 180

SRLHSYSSPS TKNSSGGGES RSSSRGGGGE SRSSGAASSA PGGGDGAEYK TLKISELGSQ

190 200 210 220 230 240

LSDEAVEDGL FHEFKRFGDV SVKISHLSGS GSGDERVAFV NFRRPEDARA AKHARGRLVL

250 260 270 280 290 300

YDRPLKIEAV YVSRRRSRSP LDKDTYPPSA SVVGASVGGH RHPPGGGGGQ RSLSPGGAAL

310 320 330 340 350 360

GYRDYRLQQL ALGRLPPPPP PPLPRDLERE RDYPFYERVR PAYSLEPRVG AGAGAAPFRE

370 380 390 400 410 420

VDEISPEDDQ RANRTLFLGN LDITVTESDL RRAFDRFGVI TEVDIKRPSR GQTSTYGFLK

430 440 450 460 470 480

FENLDMSHRA KLAMSGKIII RNPIKIGYGK ATPTTRLWVG GLGPWVPLAA LAREFDRFGT

490 500 510 520 530 540

IRTIDYRKGD SWAYIQYESL DAAHAAWTHM RGFPLGGPDR RLRVDFADTE HRYQQQYLQP

550 560 570 580 590 600

LPLTHYELVT DAFGHRAPDP LRGARDRTPP LLYRDRDRDL YPDSDWVPPP PPVRERSTRT

610 620 630 640 650 660

AATSVPAYEP LDSLDRRRDG WSLDRDRGDR DLPSSRDQPR KRRLPEESGG RHLDRSPESD

670 680 690 700 710 720

RPRKRHCAPS PDRSPELSSS RDRYNSDNDR SSRLLLERPS PIRDRRGSLE KSQGDKRDRK

730 740 750 760 770 780

NSASAERDRK HRTTAPTEGK SPLKKEDRSD GSAPSTSTAS SKLKSPSQKQ DGGTAPVASA

790 800 810 820 830 840

SPKLCLAWQG MLLLKNSNFP SNMHLLQGDL QVASSLLVEG STGGKVAQLK ITQRLRLDQP

850 860 870 880 890 900

KLDEVTRRIK VAGPNGYAIL LAVPGSSDSR SSSSSAASDT ATSTQRPLRN LVSYLKQKQA

910 920 930 940 950 960

AGVISLPVGG NKDKENTGVL HAFPPCEFSQ QFLDSPAKAL AKSEEDYLVM IIVRGFGFQI

970

GVRYENKKRE NLALTLL

SORBS2	O94875-10;O94875-12;O94875-4;O94875-3;O94875-5;O94875-11;O94875;O94875-7;O94875-2;O94875-8;O94875-9;O94875-6

10 20 30 40 50 60

MSYYQRPFSP SAYSLPASLN SSIVMQHGTS LDSTDTYPQH AQSLDGTTSS SIPLYRSSEE

70 80 90 100 110 120

EKRVTVIKAP HYPGIGPVDE SGIPTAIRTT VDRPKDWYKT MFKQIHMVHK PDDDTDMYNT

130 140 150 160 170 180

PYTYNAGLYN PPYSAQSHPA AKTQTYRPLS KSHSDNSPNA FKDASSPVPP PHVPPPVPPL

190 200 210 220 230 240

RPRDRSSTEK HDWDPPDRKV DTRKFRSEPR SIFEYEPGKS SILQHERPAS LYQSSIDRSL

250 260 270 280 290 300

ERPMSSASMA SDFRKRRKSE PAVGPPRGLG DQSASRTSPG RVDLPGSSTT LTKSFTSSSP

310 320 330 340 350 360

SSPSRAKGGD DSKICPSLCS YSGLNGNPSS ELDYCSTYRQ HLDVPRDSPR AISFKNGWQM

370 380 390 400 410 420

ARQNAEIWSS TEETVSPKIK SRSCDDLLND DCDSFPDPKV KSESMGSLLC EEDSKESCPM

430 440 450 460 470 480

AWGSPYVPEV RSNGRSRIRH RSARNAPGFL KMYKKMHRIN RKDLMNSEVI CSVKSRILQY

490 500 510 520 530 540

ESEQQHKDLL RAWSQCSTEE VPRDMVPTRI SEFEKLIQKS KSMPNLGDDM LSPVTLEPPQ

550 560 570 580 590 600

NGLCPKRRFS IEYLLEEENQ SGPPARGRRG CQSNALVPIH IEVTSDEQPR AHVEFSDSDQ

610 620 630 640 650 660

DGVVSDHSDY IHLEGSSFCS ESDFDHFSFT SSESFYGSSH HHHHHHHHHH RHLISSCKGR

670 680 690 700 710 720

CPASYTRFTT MLKHERARHE NTEEPRRQEM DPGLSKLAFL VSPVPFRRKK NSAPKKQTEK

730 740 750 760 770 780

AKCKASVFEA LDSALKDICD QIKAEKKRGS LPDNSILHRL ISELLPDVPE RNSSLRALRR

790 800 810 820 830 840

SPLHQPLHPL PPDGAIHCPP YQNDCGRMPR SASFQDVDTA NSSCHHQDRG GALQDRESPR

850 860 870 880 890 900

SYSSTLTDMG RSAPRERRGT PEKEKLPAKA VYDFKAQTSK ELSFKKGDTV YILRKIDQNW
910 920 930 940 950 960
YEGEHHGRVG IFPISYVEKL TPPEKAQPAR PPPPAQPGEI GEAIAKYNFN ADTNVELSLR
970 980 990 1000 1010 1020
KGDRVILLKR VDQNWYEGKI PGTNRQGIFP VSYVEVVKKN TKGAEDYPDP PIPHSYSSDR
1030 1040 1050 1060 1070 1080
IHSLSSNKPQ RPVFTHENIQ GGGEPFQALY NYTPRNEDEL ELRESDVIDV MEKCDDGWFV
1090 1100
GTSRRTKFFG TFPGNYVKRL

STAMBPL1	Q96FJ0;Q96FJ0-2

10 20 30 40 50 60
MDQPFTVNSL KKLAAMPDHT DVSLSPEERV RALSKLGCNI TISEDITPRR YFRSGVEMER
70 80 90 100 110 120
MASVYLEEGN LENAFVLYNK FITLFVEKLP NHRDYQQCAV PEKQDIMKKL KEIAFPRTDE
130 140 150 160 170 180
LKNDLLKKYN VEYQEYLQSK NKYKAEILKK LEHQRLIEAE RKRIAQMRQQ QLESEQFLFF
190 200 210 220 230 240
EDQLKKQELA RGQMRSQQTS GLSEQIDGSA LSCFSTHQNN SLLNVFADQP NKSDATNYAS
250 260 270 280 290 300
HSPPVNRALT PAATLSAVQN LVVEGLRCVV LPEDLCHKFL QLAESNTVRG IETCGILCGK
310 320 330 340 350 360
LTHNEFTITH VIVPKQSAGP DYCDMENVEE LFNVQDQHDL LTLGWIHTHP TQTAFLSSVD
370 380 390 400 410 420
LHTHCSYQLM LPEAIAIVCS PKHKDTGIFR LTNAGMLEVS ACKKKGFHPH TKEPRLFSIC
430
KHVLVKDIKI IVLDLR

Supplementary Figure 11: Protein sequences of post-translationally *N*-myristoylated proteins, including putative sites of modification. Below are listed the sequences (isoform 1, unless indicated otherwise) of proteins bearing PTMyr sites identified in this study (please refer to Supplementary Data 4), as reported in the UniProtKB/Swiss-Prot database. The sequences of the unique peptides detected in this study by LC-MS/MS are highlighted in yellow and grey (the two colors are used as needed to allow distinction between adjacent peptides), missed cleavage sites are underlined. The sequences of the YnMyr-modified peptides detected upon **AzKTB**/**AzRTB**-based enrichment are highlighted in cyan. Proteins are ordered by gene names and the Uniprot accession number is reported.

Fig. 2d

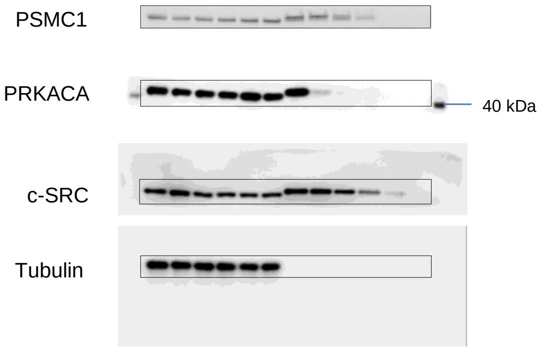

Fig. 4a

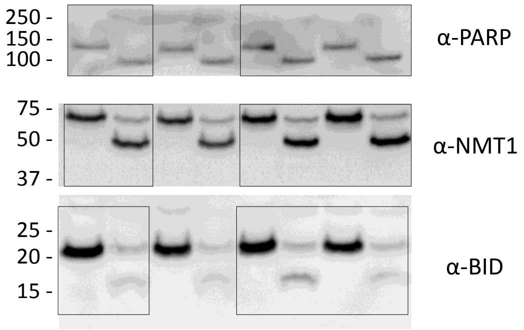

Fig. 4c

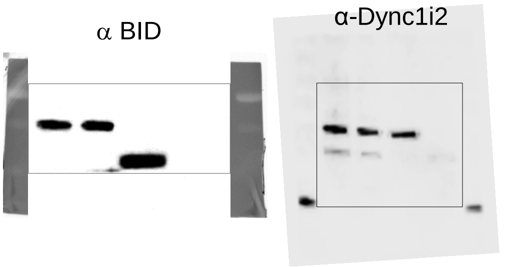

Fig. 4d

Cul4B[1-590]-FLAG (α-FLAG IP)

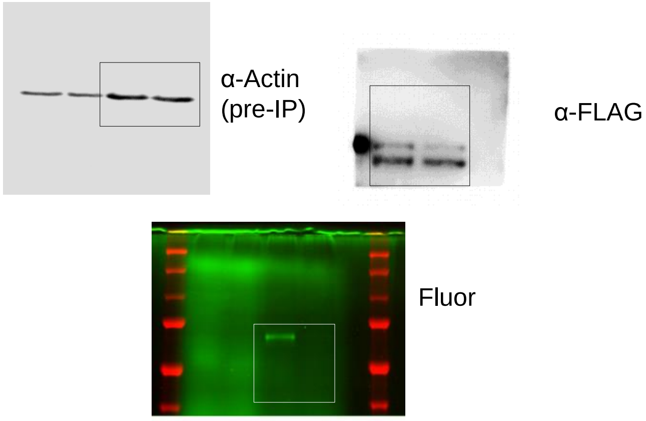

Supplementary Fig. 1g

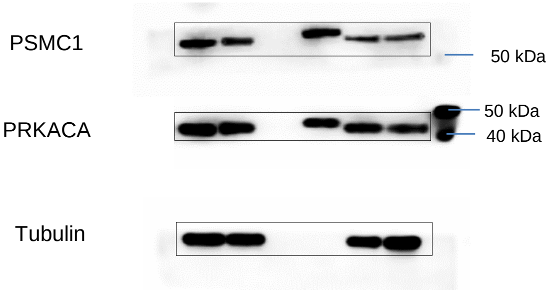

Supplementary Fig. 5

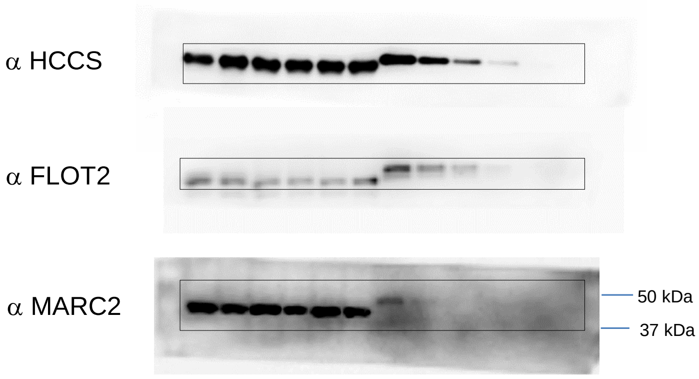

Supplementary Figure 12: Full blots and gels relating to cropped sections shown in figures. Only cropped blots and gels are shown; in some cases, membranes were cut between specific molecular weight markers prior to blotting to maximize information recovered from an experiment, resulting in smaller sections shown in some figures.

Supplementary Table 1: Previous studies of *N*-myristoylated proteins expressed in HeLa.

Proteins with an N-terminal MG motif and shown to be *N*-myristoylated in a cell-free system or *E. coli* expression system (CF), a non-native protein construct in eukaryotic cells (NN) or native proteins in eukaryotic cells (N), typically via immunoprecipitation of a radiolabeled protein. Only proteins expressed in HeLa cells are reported.[3] A column indicates whether proteins have been predicted to be *N*-myristoylated by online predictors (**Myr predictor (MP)**: R= Reliable; T= twilight, N= No. **Myristoylator (Myr)**: H= high probability; M= medium probability; L= low probability; N= not myristoylated). A column indicates if the protein is palmitoylated (+: found in 1 study, ++: found in 2 studies; +++: found in 3 studies; ++++: found in 4 studies;[4] u: reported as palmitoylated in the UniProtKB/Swiss-Prot database).

Method	Protein name	Gene name	Protein IDs	Reference	Palm	MP	Myr
N	Annexin XIII	ANXA13	P27216	5		R	H
	Brain acid soluble protein 1	BASP1	P80723	6		R	H
	Golgi-associated plant pathogenesis-related protein 1	GLIPR2	Q9H4G4	7		R	H
	MARCKS	MARCKS	P29966	8		R	H
	cAMP dependent protein kinase alpha subunit	PRKACA	P17612	9		R	H
	Tyrosine protein kinase Src	SRC	P12931	10	+	R	H
NN	ABL1	ABL1	P00519	11		T	N
	A-kinase anchor protein 12	AKAP12	Q02952	12		R	H
	A-kinase anchor protein 7	AKAP7	O43687	13	u	R	H
	ADP ribosylation factor 1	ARF1	P84077	14	++	T	H
	ADP ribosylation factor 6	ARF6	P62330	15		R	H
	ADP-ribosylation factor-like 5B	ARL5B	Q96KC2	16		N	M
	Cyclin-Y	CCNY	Q8ND76	17	+++	R	H
	Coiled-coil-helix-coiled-coil-helix domain-containing protein 3, mitochondrial	CHCHD3	Q9NX63	18	+	R	H
	Charged multivesicular body protein 6	CHMP6	Q96FZ7	19		N	H
	Calcium and integrin-binding protein 1	CIB1	Q99828	20		R	H
	NADH cytochrome b5 reductase 3	CYB5R3	P00387	21	++	R	H
	Sphingolipid delta(4)-desaturase DES1	DEGS1	O15121	22		T	H
	Dual specificity protein phosphatase 22	DUSP22	Q9NRW4	23		N	H
	Formin-like protein 1	FMNL1	O95466	24		T	N
	Fibroblast growth factor receptor substrate 2 FRS2	FRS2	Q8WU20	25		R	H
	Fibroblast growth factor receptor substrate 3 FRS3	FRS3	O43559	25		R	H
	Tyrosine protein kinase Fyn	FYN	P06241	26	u; ++	R	H
	Guanine nucleotide-binding protein G(i) subunit alpha-1	GNAI1	P63096	27	u; ++	R	H
	Guanine nucleotide-binding protein G(i) subunit alpha-2	GNAI2	P04899	27	u; ++++	R	H
	Guanine nucleotide-binding protein G(k) subunit alpha	GNAI3	P08754	27	u; ++++	R	H
	Guanine nucleotide-binding protein G(o) subunit alpha	GNAO1	P09471	27	u; +++	R	H
	Guanine nucleotide-binding protein G(z) subunit alpha	GNAZ	P19086	27	u; +	R	H
	Neuron-specific calcium-binding protein hippocalcin	HPCA	P84074	28		R	H
	Hippocalcin-like protein 1	HPCAL1	P37235	29		R	H
	LanC-like protein 2	LANCL2	Q9NS86	30		N	H
	Tyrosine protein kinase Lyn	LYN	P07948	31	u; +	R	H
	Mitochondrial peptide methionine sulfoxide reductase	MSRA	Q9UJ68	32		R	H
	Neuronal calcium sensor 1	NCS1	P62166	28		R	H
	Nephrocystin-3	NPHP3	Q7Z494	33		T	H
	cGMP-dependent 3',5'-cyclic	PDE2A	O00408	34	u	N	N

	phosphodiesterase						
	Protein phosphatase 1A	PPM1A	P35813	[35]		N	H
	Protein phosphatase 1B	PPM1B	O75688	[35]		N	M
	Calcineurin B type 1	PPP3R1	P63098	[36]		R	H
	5'-AMP-activated protein kinase subunit beta-1	PRKAB1	Q9Y478	[37]		N	N
	5'-AMP-activated protein kinase subunit beta-2	PRKAB2	O43741	[38]		N	M
	26S protease regulatory subunit 4	PSMC1	P62191	[39]	++	R	H
	Raftlin	RFTN1	Q14699	[40]	u	N	H
	Protein-associating with the carboxyl-terminal domain of ezrin (PACE-1)	SCYL3	Q8IZE3	[41]		R	H
	TIR domain-containing adapter molecule 2	TICAM2	Q86XR7	[42]		R	M
	Tyrosine protein kinase Yes	YES1	P07946	[43]	u; ++	R	H
	E3 ubiquitin-protein ligase ZNRF2	ZNRF2	Q8NHG8	[44]		R	M
	Apoptosis-inducing factor 2	AIFM2	Q9BRQ8	[45]		R	H
	Ankyrin repeat and IBR domain-containing protein 1	ANKIB1	Q9P2G1	[45]		R	H
	ADP ribosylation factor-like protein 1	ARL1	P40616	[46]		R	H
	BTB/POZ domain-containing protein 7	BTBD7	Q9P203	[45]		R	M
	Calcineurin B homologous protein 1	CHP1	Q99653	[47]		R	H
	Calcineurin B homologous protein 3	CHP3	Q96BS2	[48]		R	H
	Dixin	DIXDC1	Q155Q3	[45]		T	N
	Dymeclin	DYM	Q7RTS9	[49]		R	H
	Formin-like protein 2	FMNL2	Q96PY5	[45]		T	H
	Formin-like protein 3 (FMNL3)	FMNL3	Q8IVF7	[45]		N	M
	Golgi reassembly-stacking protein 1 (GRASP65)	GORASP1	Q9BQQ3	[50]		R	H
CF	Uncharacterized protein KIAA1522	KIAA1522	Q9P206	[45]		N	H
	TLD domain-containing protein KIAA1609	KIAA1609	Q6P9B6	[45]		T	H
	Protein Lunapark	LNP	Q9C0E8	[45]		T	H
	E3 Ubiquitin-protein ligase MGRN1	MGRN1	O60291	[45]		R	H
	NADH dehydrogenase [ubiquinone] 1 beta subcomplex subunit 7	NDUFB7	P17568	[51]		N	L
	Phosphoinositide 3-kinase regulatory subunit 4 (PIK3R4)	PIK3R4	Q99570	[52]		T	H
	RING finger protein 11	RNF11	Q9Y3C5	[45]		N	M
	Serine incorporator 1	SERINC1	Q9NRX5	[45]		N	N
	Tescalin	TESC	Q96BS2	[48]		R	H
	Zinc finger ZZ-type and EF-hand domain-containing protein 1	ZZEF1	O43149	[45]		R	H

Supplementary Table 2: Known post-translationally *N*-myristoylated proteins.

Proteins shown to be *N*-myristoylated in systems where proteins were overexpressed in eukaryotic cells (NN) or were present at native expression levels (N). A column indicates if the new N-terminal sequences have been predicted to be *N*-myristoylated by online predictors (**Myr predictor**: R= Reliable; T= twilight. **Myristoylator**: H= high probability; M= medium probability; L= low probability; N= not myristoylated).

Method	Protein name	Gene name	Protein IDs	Ref.	New N-terminal sequence	Cleavage site	MP	Myr
N	BH3-interacting domain death agonist	BID	P55957	[53]	GNRSSHSRLGRIEADSE SQEDIIRNIARHL	G60	R	H
NN	Actin, cytoplasmic 1 (β-actin)	ACTB	P60709	[54]	GQVITIGNERFRCPEAL FQPSFLGMESCGI	G245	T	N
	Cell division control protein 6 homolog	CDC6	Q99741	[55]	GNRMTLSQEGAQDSFP LQQKILVCSLMLLI	G443	R	H
	Gelsolin	GSN	P06396	[54]	GLGLSYLSSHIANVERV PFDAATLHTSTAM	G404	R	M
	Huntingtin	HTT	P42858	[55]	GTQASSPISDSSQTTTE GPDSAVTPSDSSE	G551	R	H
	Microtubule-actin cross-linking factor 1, isoforms 1/2/3/5	MACF1	Q9UPN3	[55]	GSDASQLLHQAEVAQQ EFLEVKQRVNSGCV	G5087	R	H
	Induced myeloid leukemia cell differentiation protein	MCL1	Q07820	[55]	GSLPSTPPPAEEEEDEL YRQSLEIISRYLR	G158	R	H
	p21-activated protein kinase-2	PAK2	Q13177	[56]	GAAKSLDKQKKKTKMT DEEIMEKLRTIVSI	G213	T	H
	YTH domain family protein 2	YTHDF2	Q9Y5A9	[55]	GNGVGQSQAGSGSTP SEPHPVLEKLRSINN	G367	R	M

Supplementary Methods

1.1. General methods: Synthetic procedures

All reagents and solvents were purchased from Sigma-Aldrich, NovaBiochem UK or AGTC Bioproducts and used without further purification. Ultrapure water was obtained from MilliQ® Millipore water purification system. Moisture sensitive reactions were performed under nitrogen atmosphere using dried glassware and standard syringe/septa techniques.
Thin Layer Chromatography was performed on Merck pre-coated Silica plates (Aluminum oxide 60 F254, Merck). Spots were visualized by UV light (operating at 254 nm), and using the appropriate stain. Silica gel column chromatography was carried either by hand-made columns with Merck Silica 60Å, or using an Isolera (Biotage, UK) automated apparatus with fraction collector equipped with SNAP cartridges columns (Biotage, UK).
NMR spectra were recorded on 400MHz Bruker instruments and were referenced to residual solvent signals. Data are presented as follows: chemical shift, multiplicity (s = singlet, d = doublet, t = triplet, m = multiplet), coupling constant(s) in Hz and integration. High resolution mass spectrometry (HRMS) was performed on Waters LCT Premier Spectrometer.
Analytical and semi-preparative RP-HPLC was carried out on a Waters 2767 system equipped with a photodiode array, a mass spectrometer and an X-Bridge C18 column (5 µM, 4.6 mM × 100 mM). The flow rate of 1.2 mL/min was used for the analytical mode and 20 mL/min were used for the preparative mode.
Freeze-drying was carried out using a freeze dryer Alpha 2-4 LD plus, Christ (Germany).
The syntheses of the peptide c-Src (N-terminal 8 amino acids from Gly2), Azidopropionic acid, the capture reagent AzTB (azido-tamra-biotin), the capture reagent AzKTB (azido-lysine-tamra-biotin) and inhibitor **1** have been described elsewhere. [1, 2, 57, 58]

1.2. Synthetic procedures

1.2.1. YnMyr synthesis

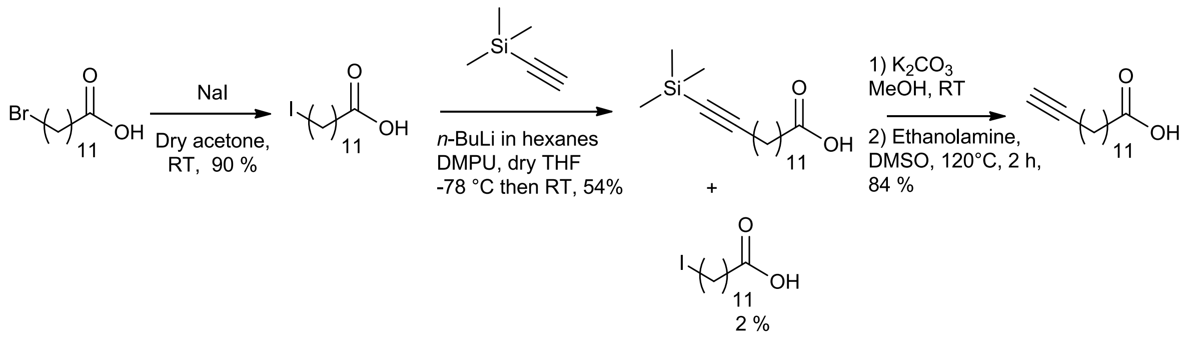

12-Iodododecanoic acid 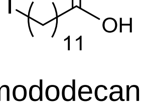

To a solution of 12-bromododecanoic acid (1.00 g, 3.58 mmol, 1 eq) in dry acetone (20 mL) was added NaI (1.61 g, 10.74 mmol, 3eq) in one portion. The mixture was stirred at room temperature overnight. Completion of the reaction was checked by ^{1}H NMR. Water (200 mL) and DCM (100 mL) were added. The layers were separated and the aqueous layer was extracted with DCM (2 x 70 mL). The combined organic layers were washed with saturated aqueous sodium thiosulfate (100 mL), brine (100 mL), dried over $MgSO_4$ and concentrated under reduced pressure to yield a white solid (1.05 g,

90%). The crude product was used without further purification for the next reaction. **^{1}H NMR** (400 MHz, $CDCl_3$) δ = 3.17 (t, *J*=7.1, 2H), 2.33 (t, *J*=7.5, 2H), 1.84 – 1.75 (m, 2H), 1.60 (m, 2H), 1.27 (m, 14H).

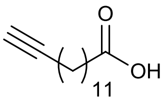

Tetradec-13-ynoic acid (YnMyr)

Under nitrogen atmosphere, a solution of TMS-acetylene (1.12 mL, 7.82 mmol, 2.5 eq) in dry THF (8 mL) was cooled down to -78 °C using a bath of acetone and dry ice. A solution of *n*-BuLi in hexanes (3.8 mL, 2.5 M solution, 9.39 mmol, 3.0 eq) was added drop wise. The clear reaction mixture was allowed to warm to room temperature for 10 minutes and then cooled to -78 °C. DMPU (7.9 mL, 21 eq) and a solution of 12-iodododecanoic acid (1.02 g, 3.13 mmol, 1.0 eq) in dry THF (8 mL) were added drop wise. The yellow reaction mixture was allowed to warm to room temperature and stirred overnight. The reaction mixture was cooled to -78 °C and quenched by the drop wise addition of saturated NH_4Cl (240 mL). Et_2O (100 mL) and water (50 mL) were added to the brown solution. The layers were separated and the aqueous layer was extracted with Et_2O (2 x 50 mL). The combined organic layers were washed with water (100 mL), brine (100 mL), dried over Na_2SO_4, filtered and concentrated under reduced pressure. The brown residue was purified by silica-gel chromatography (gradient: EtOAc: AcOH 99:0:1 to 95:4:1) to yield a white solid (500 mg, 54%). NMR analysis of the product showed that the product contained < 2% of 12-Iodododecanoic acid.

To a suspension of TMS-protected alkyne (490 mg, 1 eq, 1.65 mmol) in MeOH (10 mL) was added K_2CO_3 (457 mg, 3.31 mmol, 2 eq). The reaction mixture was stirred at room temperature overnight to give a clear reaction mixture. The mixture was concentrated under reduced pressure. The residue was taken up in 2N HCl (50 mL) and Et_2O (50 mL). The layers were separated and the aqueous layer was extracted with Et_2O (2 x 50 mL). The combined organic layers were washed with water (50 mL), brine (50 mL), dried over Na_2SO_4, filtered and concentrated under reduced pressure to yield an off-white solid (359 mg, 96%). NMR analysis of the product showed that the product contained < 2% of 12-Iodododecanoic acid.

The white solid (YnMyr + impurity (< 2%; 12-iodododecanoic acid); 340 mg, 1.52 mmol) was dissolved in DMSO (500 µL) and ethanolamine (25 eq regarding 2% impurity, 0.76 mmol, 46 µL) was added in one portion. The reaction mixture was heated to 120 °C for 2 h and then allowed to cool down to room temperature. Et_2O (100 mL) and 2N HCl (100 mL) were added. The layers were separated and the aqueous layer was extracted with Et_2O (50 mL). The combined organic layers were washed with 0.5 HCl (50 mL), brine (50 mL), dried over $MgSO_4$ and concentrated under reduced pressure to yield a white solid (296 mg, 84%; overall yield = 37%). **^{1}H NMR** (400 MHz, $CDCl_3$) δ 2.33 (t, *J* = 7.5, 2H, C*H_2*CO_2H), 2.16 (dt, *J* = 2.6, 7.1, 2H, C*H_2*C≡CH), 1.92 (t, *J* = 2.6, 1H, C≡C*H*), 1.65 – 1.55 (m, 2H, C*H_2*CH_2CO_2H), 1.54 – 1.45 (m, 2H, C*H_2*CH_2C≡CH), 1.41 – 1.19 (m, 14H, 7xC*H_2*); **^{13}C NMR** (101 MHz, $CDCl_3$) δ 179.69 (C=O), 85.03(*C*≡CH), 68.02 (C≡*C*H), 34.14, 29.5, 29.45, 29.40, 29.2, 29.1, 29.0, 28.7, 28.5, 24.87, 18.60; **HRMS** (ESI, negative mode) found 223.1698 ($[M - H]^-$ requires 223.1699).

1.2.2. **YnMyrCoA synthesis**

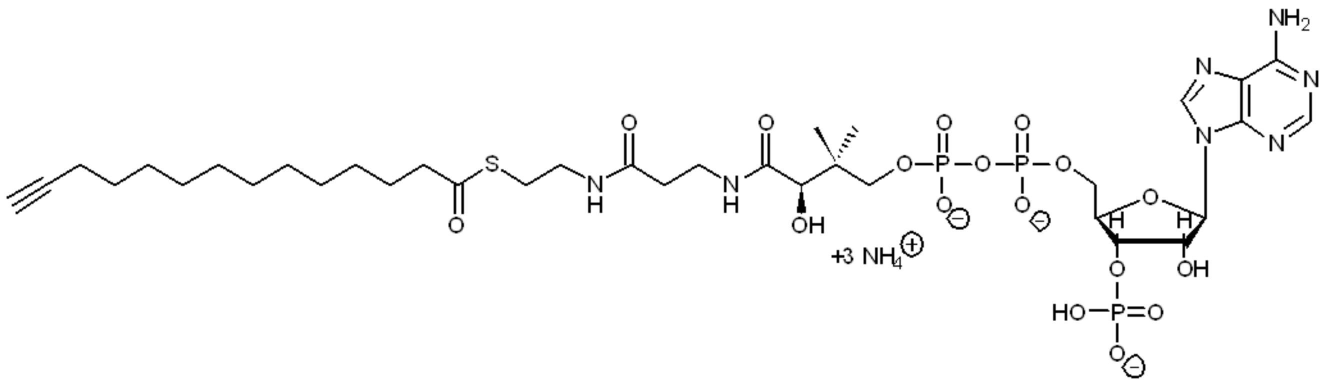

To a suspension of tetradec-13-ynoic acid (YnMyr, 14.8 mg, 65 µmol, 2 eq) in dry THF (1.0 mL) was added a solution of 1,1’-carbonyl-diimidazole (12.7 mg, 78 µmol, 2.4 eq) in DCM (1.0 mL), under nitrogen atmosphere. The clear reaction mixture was stirred for 45 min at room temperature. The reaction mixture was concentrated under reduced pressure. The yellow residue was dissolved in dry THF (1.0 mL). Co-enzyme A hydrate from yeast (25 mg, 32 µmol, 1 eq) was dissolved in an aqueous solution of $NaHCO_3$ (0.5 M, 3.4 mL) and added to the solution of activated acid. The reaction mixture was stirred at room temperature for 3 h under nitrogen atmosphere. THF was removed under reduced pressure. The product was precipitated by adding 20% perchloric acid drop wise (1 mL). The white solid was pelleted by centrifugation and washed with 1% perchloric acid and acetone. The product was purified by semi preparative LC-MS over a gradient of MeOH in 25 mM ammonium bicarbonate pH 8 (50-98 %, 20 min), YnMyrCoA was obtained as a white lyophilized solid (22.2 mg, 66% yield). Analysis was consistent with NMR and MS analysis reported in the literature.[59]

1.2.3. **MyrCoA synthesis**

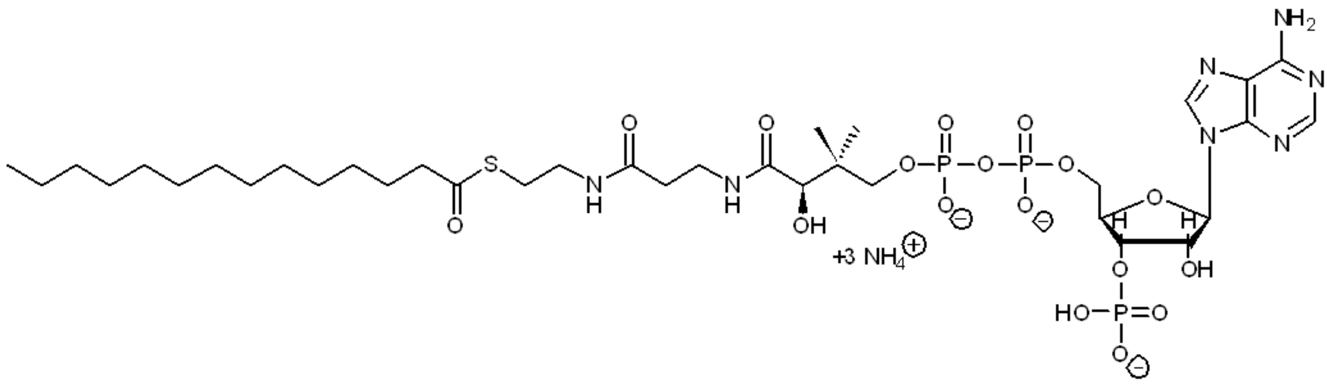

A similar procedure as YnMyrCoA was used to prepare MyrCoA. MyrCoA was obtained as a white lyophilized solid (10 mg, 32% yield). Analysis was consistent with NMR and MS analysis reported in the literature.[59]

1.2.4. **Capture reagents: AzKTB and AzRTB**

Biotin-PEG Novatag™ resin (0.47 mmol/g loading, 106.4 mg, 50.0 µmol, 1 eq) was swollen in DMF (2 mL, 30 min), Fmoc deprotected with 20% v/v piperidine in DMF (2 mL, 10 min × 3) and washed with DMF, DCM and DMF sequentially. Fmoc-Lysine(Mmt)-OH (96.1 mg, 3 eq), HATU (57 mg, 3 eq) and DIPEA (52.3 µL, 6 eq) were dissolved in DMF (1 mL), added to the deprotected resin and the reaction was shaken for 2h after which the procedure was repeated. All subsequent couplings, i.e. Fmoc-6-Ahx-OH, Fmoc-Gly-OH, Fmoc-Lys(Boc)-OH (Az-KTB) or Fmoc-Arg(Pbf)-OH (Az-RTB) , Fmoc-Ala-OH,

and Azidopropionic acid (all 5 eq) were performed using DIC/HOBt activation (5 eq each, 30 min x 2). Following the removal of Mmt protecting group with 1 % TFA in DCM (10 min x 4) and wash (DCM, DMF), TAMRA (43 mg, 2 eq) was activated for 10 min in DMF (1 mL) with DIC (15.7 mg, 2 eq) and HOAt (13.6 mg, 2 eq) and coupled to the peptidyl resin (2 h x 2). The crude product was then cleaved from the resin with 95 % TFA, 2.5 % water and 2.5 % triisopropylsilane (3 h) and precipitated with cold TBME. The solids were pelleted by centrifugation (15 min, 4300 rpm, 4 °C) and washed three times with TBME. The pelleted product was dried and purified by semi preparative LC-MS over a gradient of MeOH (0.1% FA) in water (0.1% FA) (2-98 %, 15 min), with detection over 100–600 nm. The product was obtained by lyophilisation as a bright pink amorphous solid (Az-KTB: 12 mg, 17 % yield; Az-RTB: 15 mg, 20 %). HRMS m/z (ESI), calculated for Az-KTB, $C_{71}H_{104}N_{16}O_{15}S$ ($[M + 2H]^{2+}$) 727.3873, found 727.3892; calculated for Az-RTB, $C_{71}H_{104}N_{18}O_{15}S$ ($[M + 2H]^{2+}$) 741.3904, found 741.3847.

Supplementary References

1. Goncalves, V. et al. A fluorescence-based assay for N-myristoyltransferase activity. *Analytical Biochemistry* **421**, 342-344 (2012).
2. Heal, W.P., Wright, M.H., Thinon, E. & Tate, E.W. Multifunctional protein labeling via enzymatic N-terminal tagging and elaboration by click chemistry. *Nat. Protocols* **7**, 105-117 (2012).
3. Nagaraj, N. et al. Deep proteome and transcriptome mapping of a human cancer cell line. *Molecular Systems Biology* **7** (2011).
4. Wilson, J.P., Raghavan, A.S., Yang, Y.-Y., Charron, G. & Hang, H.C. Proteomic Analysis of Fatty-acylated Proteins in Mammalian Cells with Chemical Reporters Reveals S-Acylation of Histone H3 Variants. *Molecular & Cellular Proteomics* **10** (2011).
5. Wice, B.M. & Gordon, J.I. A strategy for isolation of cDNAs encoding proteins affecting human intestinal epithelial cell growth and differentiation: characterization of a novel gut-specific N-myristoylated annexin. *The Journal of Cell Biology* **116**, 405-422 (1992).
6. Mosevitsky, M.I. et al. The BASP1 family of myristoylated proteins abundant in axonal termini. Primary structure analysis and physico-chemical properties. *Biochimie* **79**, 373-384 (1997).
7. Eberle, H.B. et al. Identification and characterization of a novel human plant pathogenesis-related protein that localizes to lipid-enriched microdomains in the Golgi complex. *Journal of Cell Science* **115**, 827-838 (2002).
8. McIlhinney, R.A. & McGlone, K. Evidence for a non-myristoylated pool of the 80 kDa protein kinase C substrate of rat brain. *Biochemical Journal* **271**, 681-685 (1990).
9. Carr, S.A., Biemann, K., Shoji, S., Parmelee, D.C. & Titani, K. n-Tetradecanoyl is the NH2-terminal blocking group of the catalytic subunit of cyclic AMP-dependent protein kinase from bovine cardiac muscle. *Proceedings of the National Academy of Sciences* **79**, 6128-6131 (1982).
10. Buss, J.E. & Sefton, B.M. Myristic acid, a rare fatty acid, is the lipid attached to the transforming protein of Rous sarcoma virus and its cellular homolog. *Journal of Virology* **53**, 7-12 (1985).
11. Jackson, P. & Baltimore, D. N-terminal mutations activate the leukemogenic potential of the myristoylated form of c-abl. *EMBO Journal* **8**, 449-456 (1989).
12. Streb, J.W., Kitchen, C.M., Gelman, I.H. & Miano, J.M. Multiple Promoters Direct Expression of Three AKAP12 Isoforms with Distinct Subcellular and Tissue Distribution Profiles. *Journal of Biological Chemistry* **279**, 56014-56023 (2004).
13. Fraser, I.D.C. et al. A novel lipid-anchored A-kinase Anchoring Protein facilitates cAMP-responsive membrane events. *EMBO Journal* **17**, 2261-2272 (1998).
14. Kahn, R.A., Goddard, C. & Newkirk, M. Chemical and immunological characterization of the 21-kDa ADP-ribosylation factor of adenylate cyclase. *Journal of Biological Chemistry* **263**, 8282-8287 (1988).
15. D'Souza-Schorey, C. & Stahl, P.D. Myristoylation is required for the intracellular localization and endocytic function of ARF6. *Experimental Cell Research* **221**, 153-159 (1995).
16. Lin, C.-Y., Li, C.-C., Huang, P.-H. & Lee, F.-J.S. A developmentally regulated ARF-like 5 protein (ARL5), localized to nuclei and nucleoli, interacts with heterochromatin protein 1. *Journal of Cell Science* **115**, 4433-4445 (2002).
17. Jiang, M., Gao, Y., Yang, T., Zhu, X. & Chen, J. Cyclin Y, a novel membrane-associated cyclin, interacts with PFTK1. *FEBS Letters* **583**, 2171-2178 (2009).

18. Darshi, M. et al. ChChd3, an inner mitochondrial membrane protein, is essential for maintaining crista integrity and mitochondrial function. *Journal of Biological Chemistry* **286**, 2918-2932 (2011).
19. Yorikawa, C. et al. Human CHMP6, a myristoylated ESCRT-III protein, interacts directly with an ESCRT-II component EAP20 and regulates endosomal cargo sorting. *Biochemical Journal* **387**, 17-26 (2005).
20. Stabler, S.M., Ostrowski, L.L., Janicki, S.M. & Monteiro, M.J. A Myristoylated Calcium-binding Protein that Preferentially Interacts with the Alzheimer's Disease Presenilin 2 Protein. *The Journal of Cell Biology* **145**, 1277-1292 (1999).
21. Borgese, N., Aggujaro, D., Carrera, P., Pietrini, G. & Bassetti, M. A role for N-myristoylation in protein targeting: NADH-cytochrome b5 reductase requires myristic acid for association with outer mitochondrial but not ER membranes. *The Journal of Cell Biology* **135**, 1501-1513 (1996).
22. Beauchamp, E. et al. Myristic acid increases the activity of dihydroceramide Δ4-desaturase 1 through its N-terminal myristoylation. *Biochimie* **89**, 1553-1561 (2007).
23. Schwertassek, U. et al. Myristoylation of the dual-specificity phosphatase c-JUN N-terminal kinase (JNK) stimulatory phosphatase 1 is necessary for its activation of JNK signaling and apoptosis. *FEBS Journal* **277**, 2463-2473 (2010).
24. Han, Y. et al. Formin-like 1 (FMNL1) Is Regulated by N-terminal Myristoylation and Induces Polarized Membrane Blebbing. *Journal of Biological Chemistry* **284**, 33409-33417 (2009).
25. Xu, H., Lee, K.W. & Goldfarb, M. Novel Recognition Motif on Fibroblast Growth Factor Receptor Mediates Direct Association and Activation of SNT Adapter Proteins. *Journal of Biological Chemistry* **273**, 17987-17990 (1998).
26. Peters, D.J., McGrew, B.R., Perron, D.C., Liptak, L.M. & Laudano, A.P. In vivo phosphorylation and membrane association of the fyn proto-oncogene product in IM-9 human lymphoblasts. *Oncogene* **5**, 1313-1319 (1990).
27. Mumby, S.M., Heukeroth, R.O., Gordon, J.I. & Gilman, A.G. G-protein alpha-subunit expression, myristoylation, and membrane association in COS cells. *Proceedings of the National Academy of Sciences* **87**, 728-732 (1990).
28. O'Callaghan, D.W. et al. Differential Use of Myristoyl Groups on Neuronal Calcium Sensor Proteins as a Determinant of Spatio-temporal Aspects of Ca2+ Signal Transduction. *Journal of Biological Chemistry* **277**, 14227-14237 (2002).
29. Spilker, C. et al. The neuronal EF-hand calcium-binding protein visinin-like protein-3 is expressed in cerebellar Purkinje cells and shows a calcium-dependent membrane association. *Neuroscience* **96**, 121-129 (2000).
30. Landlinger, C., Salzer, U. & Prohaska, R. Myristoylation of human LanC-like Protein 2 (LANCL2) is essential for the interaction with the plasma membrane and the increase in cellular sensitivity to adriamycin. *Biochimica et Biophysica Acta (BBA) - Biomembranes* **1758**, 1759-1767 (2006).
31. Kovářová, M. et al. Structure-Function Analysis of Lyn Kinase Association with Lipid Rafts and Initiation of Early Signaling Events after Fcε Receptor I Aggregation. *Molecular and Cellular Biology* **21**, 8318-8328 (2001).
32. Kim, G., Cole, N.B., Lim, J.C., Zhao, H. & Levine, R.L. Dual Sites of Protein Initiation Control the Localization and Myristoylation of Methionine Sulfoxide Reductase A. *Journal of Biological Chemistry* **285**, 18085-18094 (2010).
33. Wright, K.J. et al. An ARL3–UNC119–RP2 GTPase cycle targets myristoylated NPHP3 to the primary cilium. *Genes & Development* **25**, 2347-2360 (2011).
34. Russwurm, C., Zoidl, G., Koesling, D. & Russwurm, M. Dual Acylation of PDE2A Splice Variant 3: TARGETING TO SYNAPTIC MEMBRANES. *Journal of Biological Chemistry* **284**, 25782-25790 (2009).
35. Chida, T. et al. N-Myristoylation is essential for protein phosphatases PPM1A and PPM1B to dephosphorylate their physiological substrates in cells. *Biochemical Journal* **449**, 741-749 (2013).
36. Aitken, A. et al. Identification of the NH2-terminal blocking group of calcineurin B as myristic acid. *FEBS Letters* **150**, 314-318 (1982).
37. Mitchelhill, K.I. et al. Posttranslational modifications of the 5'-AMP-activated protein kinase beta1 subunit. *Journal of Biological Chemistry* **272**, 24475-24479 (1997).
38. Oakhill, J.S. et al. beta-Subunit myristoylation is the gatekeeper for initiating metabolic stress sensing by AMP-activated protein kinase (AMPK). *Proceedings of the National Academy of Sciences of the United States of America* **107**, 19237-19241 (2010).

39. Wang, X. et al. Mass spectrometric characterization of the affinity-purified human 26S proteasome complex. *Biochemistry* **46**, 3553-3565 (2007).
40. Saeki, K., Miura, Y., Aki, D., Kurosaki, T. & Yoshimura, A. The B cell-specific major raft protein, Raftlin, is necessary for the integrity of lipid raft and BCR signal transduction. *EMBO Journal* **22**, 3015-3026 (2003).
41. Sullivan, A., Uff, C.R., Isacke, C.M. & Thorne, R.F. PACE-1, a novel protein that interacts with the C-terminal domain of ezrin. *Experimental Cell Research* **284**, 222-236 (2003).
42. Rowe, D.C. et al. The myristoylation of TRIF-related adaptor molecule is essential for Toll-like receptor 4 signal transduction. *Proceedings of the National Academy of Sciences* **103**, 6299-6304 (2006).
43. Martin, D.D.O. et al. Rapid detection, discovery, and identification of post-translationally myristoylated proteins during apoptosis using a bio-orthogonal azidomyristate analog. *The FASEB Journal* **22**, 797-806 (2008).
44. Hoxhaj, G. et al. ZNRF2 is released from membranes by growth factors and, together with ZNRF1, regulates the Na+/K+ATPase. *Journal of Cell Science* **125**, 4662-4675 (2012).
45. Suzuki, T. et al. Strategy for comprehensive identification of human N-myristoylated proteins using an insect cell-free protein synthesis system. *Proteomics* **10**, 1780-1793 (2010).
46. Lee, F.-J.S. et al. Characterization of an ADP-ribosylation Factor-like 1 Protein inSaccharomyces cerevisiae. *Journal of Biological Chemistry* **272**, 30998-31005 (1997).
47. Barroso, M.R. et al. A Novel Ca-binding Protein, p22, Is Required for Constitutive Membrane Traffic. *Journal of Biological Chemistry* **271**, 10183-10187 (1996).
48. Gutierrez-Ford, C. et al. Characterization of tescalcin, a novel EF-hand protein with a single Ca2+-binding site: metal-binding properties, localization in tissues and cells, and effect on calcineurin. *Biochemistry* **42**, 14553-14565 (2003).
49. Dimitrov, A. et al. The gene responsible for Dyggve-Melchior-Clausen syndrome encodes a novel peripheral membrane protein dynamically associated with the Golgi apparatus. *Human Molecular Genetics* **18**, 440-453 (2009).
50. Barr, F.A., Puype, M., Vandekerckhove, J. & Warren, G. GRASP65, a Protein Involved in the Stacking of Golgi Cisternae. *Cell* **91**, 253-262 (1997).
51. Walker, J.E. et al. Sequences of 20 subunits of NADH: Ubiquinone oxidoreductase from bovine heart mitochondria: Application of a novel strategy for sequencing proteins using the polymerase chain reaction. *Journal of Molecular Biology* **226**, 1051-1072 (1992).
52. Panaretou, C., Domin, J., Cockcroft, S. & Waterfield, M.D. Characterization of p150, an Adaptor Protein for the Human Phosphatidylinositol (PtdIns) 3-Kinase: SUBSTRATE PRESENTATION BY PHOSPHATIDYLINOSITOL TRANSFER PROTEIN TO THE p150·;PtdIns 3-KINASE COMPLEX. *Journal of Biological Chemistry* **272**, 2477-2485 (1997).
53. Zha, J., Weiler, S., Oh, K.J., Wei, M.C. & Korsmeyer, S.J. Posttranslational N-myristoylation of BID as a molecular switch for targeting mitochondria and apoptosis. *Science* **290**, 1761-1765 (2000).
54. Utsumi, T., Sakurai, N., Nakano, K. & Ishisaka, R. C-terminal 15 kDa fragment of cytoskeletal actin is posttranslationally N-myristoylated upon caspase-mediated cleavage and targeted to mitochondria. *FEBS Lett* **539**, 37-44 (2003).
55. Martin, D.D. et al. Tandem reporter assay for myristoylated proteins post-translationally (TRAMPP) identifies novel substrates for post-translational myristoylation: PKCepsilon, a case study. *FASEB J* **26**, 13-28 (2012).
56. Vilas, G.L. et al. Posttranslational myristoylation of caspase-activated p21-activated protein kinase 2 (PAK2) potentiates late apoptotic events. *Proc Natl Acad Sci U S A* **103**, 6542-6547 (2006).
57. Srinivasan, R. et al. High-throughput synthesis of azide libraries suitable for direct "click" chemistry and in situ screening. *Org. Biomol. Chem.* **7**, 1821-1828 (2009).
58. Wright, M.H. et al. Validation of N-myristoyltransferase as an antimalarial drug target using an integrated chemical biology approach. *Nature Chem.* **6**, 112-121 (2014).
59. Heal, W.P., Wickramasinghe, S.R., Leatherbarrow, R.J. & Tate, E.W. N-Myristoyl transferase-mediated protein labelling in vivo. *Organic & Biomolecular Chemistry* **6**, 2308-2315 (2008).
